# Developing Next‐Generation Protein‐Based Vaccines Using High‐Affinity Glycan Ligand‐Decorated Glyconanoparticles

**DOI:** 10.1002/advs.202204598

**Published:** 2022-11-18

**Authors:** Yanan Gao, Wei Wang, Yunru Yang, Qingyu Zhao, Chendong Yang, Xiaoying Jia, Yang Liu, Minmin Zhou, Weihong Zeng, Xuefei Huang, Sandra Chiu, Tengchuan Jin, Xuanjun Wu

**Affiliations:** ^1^ National Glycoengineering Research Center Shandong Key Laboratory of Carbohydrate Chemistry and Glycobiology NMPA Key Laboratory for Quality Research and Evaluation of Carbohydrate‐based Medicine Shandong University Qingdao Shandong 266237 China; ^2^ State Key Laboratory of Virology Wuhan Institute of Virology Center for Biosafety Mega‐Science Chinese Academy of Sciences Wuhan 430071 China; ^3^ University of the Chinese Academy of Sciences Beijing 100049 China; ^4^ Department of Basic Medical Sciences Division of Molecular Medicine Division of Life Sciences and Medicine University of Science and Technology of China Hefei Anhui 230001 China; ^5^ Departments of Chemistry and Biomedical Engineering Institute for Quantitative Health Science and Engineering Michigan State University East Lansing Michigan 48824 United States; ^6^ Division of Life Sciences and Medicine University of Science and Technology of China Hefei Anhui 230001 China; ^7^ Suzhou Research Institute Shandong University Suzhou Jiangsu 215123 China

**Keywords:** antibody, cancer, cytotoxic T lymphocyte, glyconanoparticle, SARS‐CoV‐2

## Abstract

Major diseases, such as cancer and COVID‐19, are frightening global health problems, and sustained action is necessary to develop vaccines. Here, for the first time, ethoxy acetalated dextran nanoparticles (Ace‐Dex‐NPs) are functionalized with 9‐*N*‐(4H‐thieno[3,2‐c]chromene‐2‐carbamoyl)‐Sia*α*2−3Gal*β*1−4GlcNAc (^TCC^Sia‐LacNAc) targeting macrophages as a universal vaccine design platform. First, azide‐containing oxidized Ace‐Dex‐NPs are synthesized. After the NPs are conjugated with ovalbumin (OVA) and resiquimod (Rd), they are coupled to ^TCC^Sia‐LacNAc‐DBCO to produce ^TCC^Sia‐Ace‐Dex‐OVA‐Rd, which induce a potent, long‐lasting OVA‐specific cytotoxic T‐lymphocyte (CTL) response and high anti‐OVA IgG, providing mice with superior protection against tumors. Next, this strategy is exploited to develop vaccines against infection by severe acute respiratory syndrome coronavirus‐2 (SARS‐CoV‐2). The receptor‐binding domain (RBD) of the SARS‐CoV‐2 spike protein is the main target for neutralizing antibodies. The ^TCC^Sia‐Ace‐Dex platform is preferentially used for designing an RBD‐based vaccine. Strikingly, the synthetic ^TCC^Sia‐Ace‐Dex‐RBD‐Rd elicited potent RBD‐neutralizing antibodies against live SARS‐CoV‐2 infected Vero E6 cells. To develop a universal SARS‐CoV‐2 vaccine, the ^TCC^Sia‐Ace‐Dex‐N‐Rd vaccine carrying SARS‐CoV‐2 nucleocapsid protein (N) is also prepared, which is highly conserved among SARS‐CoV‐2 and its variants of concern (VOCs), including Omicron (BA.1 to BA.5); this vaccine can trigger strong N‐specific CTL responses against target cells infected with SARS‐CoV‐2 and its VOCs.

## Graphical abstract

By sequentially conjugating a protein antigen (PA), resiquimod (Rd), and 9‐*N*‐(4H‐thieno[3,2‐c]chromene‐2‐carbamoyl)‐Sia*α*2‐3Gal*β*1‐4GlcNAc*β*Pro‐DBCO (^TCC^Sia‐LacNAc‐DBCO) with partially oxidized azide‐displaying ethoxy acetalated dextran (Oxi‐Ace‐Dex‐Az) nanoparticles, a versatile ^TCC^Sia‐Ace‐Dex‐PA‐Rd platform is developed. This novel platform has been successfully applied to develop next‐generation protein‐based vaccines against cancer, severe acute respiratory syndrome coronavirus‐2 (SARS‐CoV‐2) and its variants of concern (VOCs).

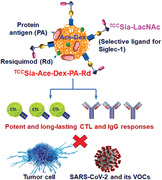

## 1 Introduction

Vaccines have eradicated smallpox, nearly eradicated polio, and prevented many infectious diseases that cause considerable morbidity and mortality each year.^[^ ^1^ ^]^ Nonetheless, past strategies of vaccine development have failed to treat most cancers. Furthermore, current vaccines do not provide complete protection against infection by severe acute respiratory syndrome coronavirus 2 (SARS‐CoV‐2), especially its variants of concern (VOCs), including Omicron (BA.1 to BA.5).^[^ ^2^ ^]^ Therefore, next‐generation vaccines against these diseases urgently need to be developed.

Epitope‐based vaccines have been widely developed, including peptide, carbohydrate, and glycopeptide vaccines.^[^ ^3^ ^]^ However, identifying critical epitopes is time‐consuming and challenging to design these vaccines. Developing protein‐based vaccines is a straightforward and effective strategy to address a sudden global health crisis, such as SARS‐CoV‐2 infection.^[^ ^4^ ^]^ The crucial problem is that directly administrating proteins often leads to insufficient immune responses, resulting in poor outcomes of disease treatment. To enhance the immunogenicity of proteins, they are usually immunized together with adjuvants, including aluminum hydroxide (Alum),^[^ ^5^ ^]^ Alum + oligodeoxynucleotides (CpG),^[^ ^6^ ^]^ and Matrix‐M.^[^ ^7^ ^]^ Compared with mixed adjuvants, using nanocarriers to deliver proteins and adjuvants is more promising for enhancing adaptive immune responses.

As a high‐potential carrier, acetalated dextran (Ac‐Dex) outperformed the commonly used poly(lactic‐*co*‐glycolic acid) (PLGA) in enhancing CTL responses.^[^ ^8^ ^]^ However, one of the degradation products of Ac‐Dex is methanol,^[^ ^8b^ ^]^ which can lead to toxicity issues. Compared with Ac‐Dex, ethoxy‐derivatized acetalated dextran (Ace‐Dex) is more biocompatible because Ace‐Dex produces ethanol.^[^ ^9^ ^]^ Accordingly, we investigated whether partially oxidized Ace‐Dex NPs can be used to deliver protein antigens (PAs). In the reported studies,^[^ ^8^, ^10^ ^]^ the uptake of Ac‐Dex NPs by immune cells occurred through passive transport; however, delivering antigens to antigen‐presenting cells (APCs) through an actively targeted delivery can be a more effective strategy to enhance adaptive immunity. Macrophages are an important class of APCs. Therefore, designing an Ace‐Dex NP vaccine targeting macrophages is an appealing strategy for developing the next‐generation vaccine.

Sialoadhesin (Siglec‐1, CD169), a sialic acid (Sia)‐binding immunoglobulin‐like lectin on macrophages, is an excellent target for developing targeting systems. While the liposomal display of a high‐affinity Siglec‐1 ligand, 9‐*N*‐(4H‐thieno[3,2‐c]chromene‐2‐carbamoyl)‐Sia*α*2−3Gal*β*1−4GlcNAc (^TCC^Sia‐LacNAc), can help selectively target CD169 for robust T‐cell activation,^[^ ^11^ ^]^ to the best of our knowledge, the impacts of ^TCC^Sia‐LacNAc on the humoral response have not been reported. Furthermore, there are no reports of ^TCC^Sia‐LacNAc targeted anti‐SARS‐CoV‐2 vaccines.

**Scheme 1:**
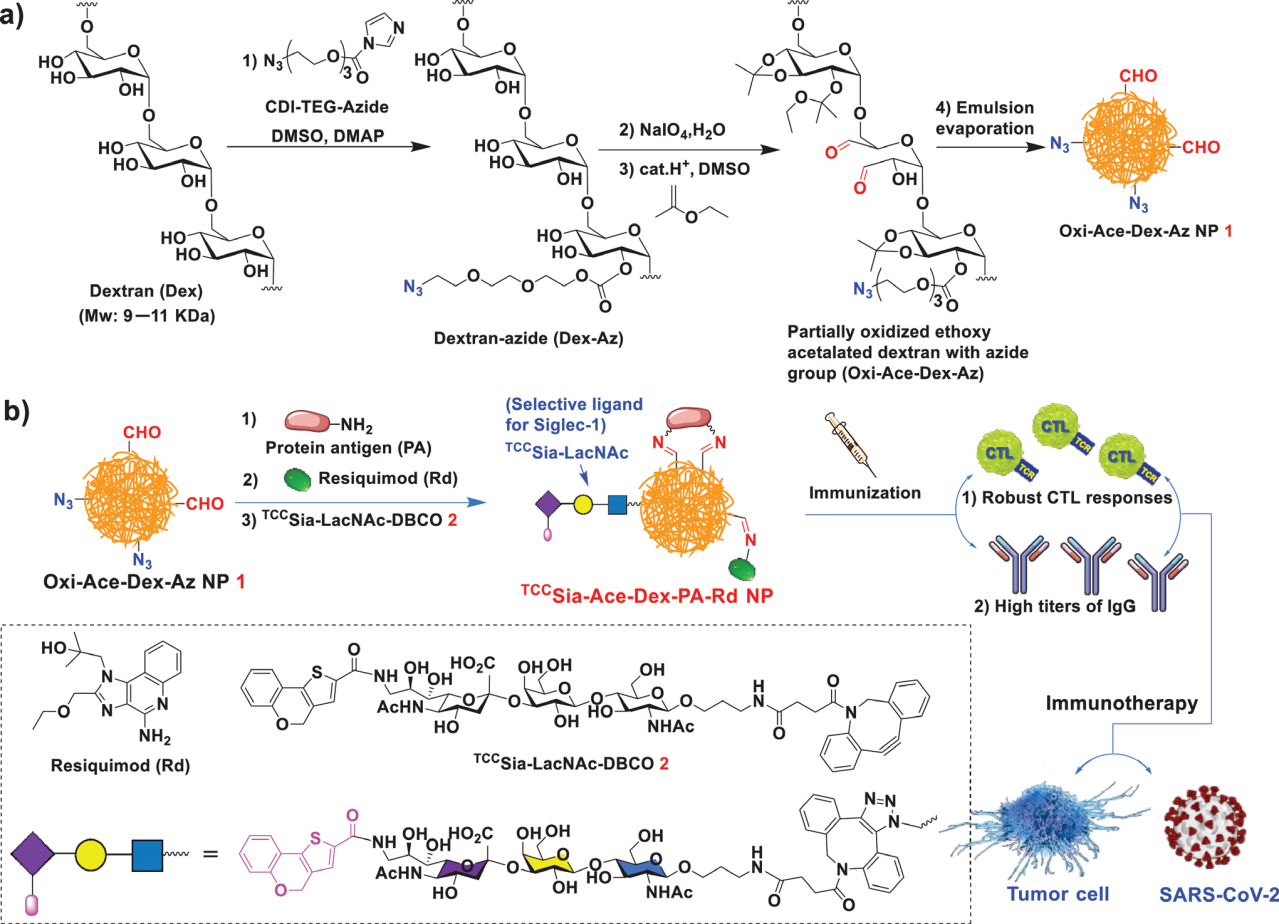
a) Synthesis of Oxi‐Ace‐Dex‐Az nanoparticles (NPs). b) Schematic diagram of the proposed ^TCC^Sia‐Ace‐Dex‐PA‐Rd NPs by conjugating protein antigen (PA) and resiquimod (Rd) with Oxi‐Ace‐Dex‐Az NPs via formation of imine bonds. The proposed ^TCC^Sia‐Ace‐Dex‐PA‐Rd NPs can enhance cytotoxic T‐lymphocyte (CTL) activities and IgG responses against tumor cells and SARS‐CoV‐2 infection.

Herein, we explored ^TCC^Sia‐LacNAc‐grafted Ace‐Dex NPs targeting macrophages, a novel antigen delivery platform, for developing next‐generation protein‐based vaccines. We first fabricated partially oxidized Ace‐Dex NPs with azide groups (Oxi‐Ace‐Dex‐Az NPs). Then, conjugation with PA and an immune‐enhancing adjuvant resiquimod (Rd) was performed via imine bond formation, and the NPs were coupled with 9‐*N*‐(4H‐thieno[3,2‐*c*]chromene‐2‐carbamoyl)‐Sia*α*2−3Gal*β*1−4GlcNAc*β*Pro‐DBCO (^TCC^Sia‐LacNAc‐DBCO) via strain‐promoted alkyne‐azide cycloaddition (SPAAC), affording ^TCC^Sia‐Ace‐Dex‐PA‐Rd platform (**Scheme** **1**a). This novel platform has been successfully applied to develop next‐generation protein‐based vaccines against cancer, SARS‐CoV‐2 and its VOCs.

## 2 Results and Discussions

### 2.1 Synthesis and Characterization of Oxi‐Ace‐Dex‐Az NPs (1)

The reaction of commonly used dextran (Dex) from *Leuconostoc mesenteroides* (molecular weight: 9−11 kDa)^[^ ^8^, ^9^, ^12^ ^]^ with imidazole‐1‐carboxylic acid 2‐[2‐(2‐azido‐ethoxy)‐ethoxy]‐ethyl ester (CDI‐TEG‐azide)^[^ ^13^ ^]^ in the presence of 4‐dimethylaminopyridine (DMAP) led to dextran‐azide (Dex‐Az). Oxidation of Dex‐Az with sodium periodate yielded partially oxidized azide‐bearing dextran (Oxi‐Dex‐Az). The aldehyde content in the Oxi‐Dex‐Az polymer was determined by a microplate bicinchoninic acid (BCA) assay. It was found that 8.2 moles of aldehyde groups were present per 100 moles of anhydrous glucose.

**Figure 1:**
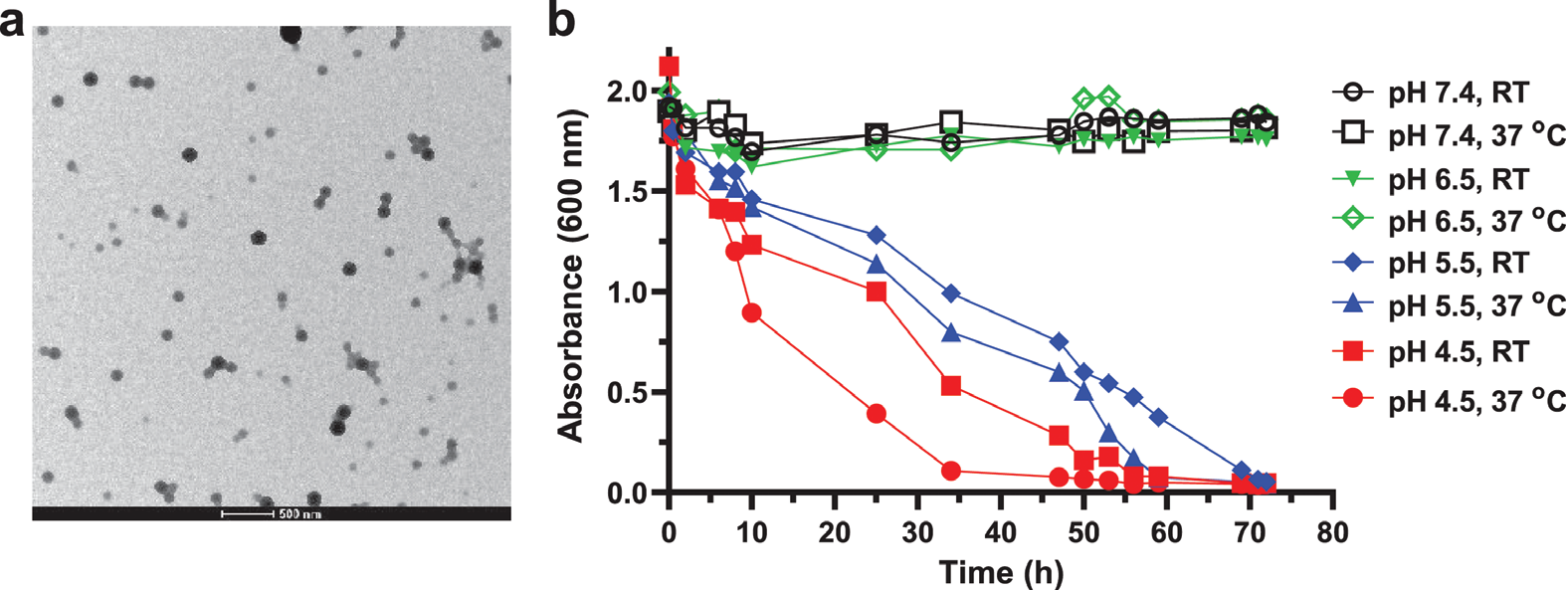
a) Transmission electron microscopy (TEM) image of Oxi‐Ace‐Dex‐Az nanoparticles (NPs) (**1**). Scale bar: 500 nm. b) Degradation of **1** in phosphate‐buffered saline (PBS) with pH 4.5, 5.5, 6.5, or 7.4 at room temperature (RT) or 37 °C as monitored by UV‐Vis absorbance at 600 nm.

Next, the Oxi‐Dex‐Az polymer was treated with 2‐ethoxypropene in the presence of pyridinium *p*‐toluenesulfonate to obtain a partially oxidized Ace‐Dex polymer with azide (Oxi‐Ace‐Dex‐Az) (Scheme 1a). By the double emulsion evaporation technique, Oxi‐Ace‐Dex‐Az NPs (**1**) were prepared, which can be used for protein and adjuvant conjugation. The obtained NPs were characterized by transmission electron microscopy (TEM) and exhibited an average size of 94 nm (**Figure** **1**a). Fourier transform infrared (FTIR) spectroscopy analysis revealed a signal at 1735 cm^−1^ in the Oxi‐Dex‐Az polymer and NP **1**, which was absent in Dex‐Az and Dex polymers without oxidation (Figure S1, Supporting Information), indicating the presence of aldehyde groups on NP **1**. To our knowledge, no studies have reported Oxi‐Ace‐Dex‐Az NPs.

Because acetal bonds are hydrolyzed by acid, Ace‐Dex NP can be degraded in acidic organelles, including late endosomes or lysosomes, bestowing its good biocompatibility. To confirm the pH responsiveness of Oxi‐Ace‐Dex‐Az NPs, they were added to phosphate‐buffered saline (PBS) at pH 7.4 and 4.5 at room temperature (RT) and 37 °C, respectively. UV‐Vis diffraction of the NP solution at 600 nm was recorded. As shown in Figure 1b, the NPs were gradually degraded in PBS at pH 5.5 and 4.5. The degradation occurred more quickly as the temperature increased and the acidity strengthened. In contrast, slight absorbance changes were observed in PBS at pH 7.4 and 6.5, indicating that the NPs were largely intact under these conditions.

### 2.2 Synthesis of ^TCC^Sia‐LacNAc‐DBCO (2)

**Scheme 2:**
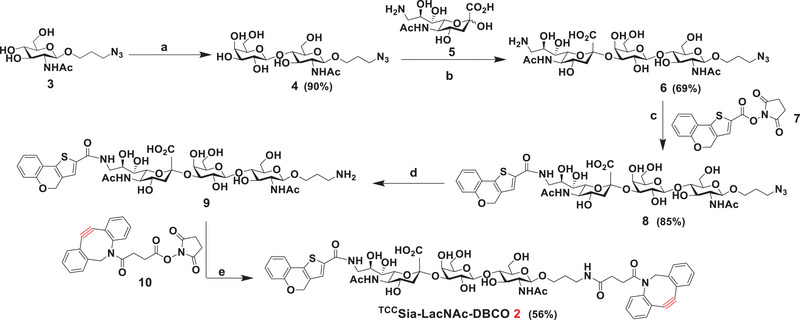
Synthesis of ^TCC^Sia‐LacNAc‐DBCO (**2**). Reagents and conditions: a) **3** (1.0 equiv), UDP‐Gal (1.3 equiv), MgCl_2_ (20 × 10^−3^ m), Tris‐HCl buffer (100 × 10^−3^ m, pH 7.5), LgtB, 37 °C, overnight; b) **4** (1.0 equiv), **5** (1.5 equiv), cytidine‐5′‐triphosphate (CTP; 1.5 equiv), MgCl_2_ (20 × 10^−3^ m), Tris‐HCl buffer (100 × 10^−3^ m, pH 8.5), *Neisseria meningitidis* CMP‐sialic acid synthetase (NmCSS), PmST1, 37 °C, overnight; c) **6** (1.0 equiv), **7** (1.5 equiv), THF/H_2_O, TEA; d) Pd/C, H_2_, CH_3_OH/H_2_O; e) **9** (1.0 equiv), **10** (1.5 equiv), THF/H_2_O, TEA.

The chemoenzymatic synthesis of ^TCC^Sia‐LacNAc‐DBCO (**2**) started from chemically prepared GlcNAc*β*ProN_3_ (**3**)^[^ ^14^ ^]^ (Scheme S1, Supporting Information), which was treated with *β*1,4‐galactosyltransferase (Lgtb) in the presence of UDP‐galactose (UDP‐Gal) at pH 7.5 forming Gal*β*1−4GlcNAc*β*ProN_3_ (**4**) in 90% yield. Disaccharide **4** was then treated with *Pasteurella multocida α*2,3‐sialyltransferase (PmST1),^[^ ^15^ ^]^ and *Neisseria meningitidis* CMP‐sialic acid synthetase (NmCSS)^[^ ^16^ ^]^ in the presence of cytidine‐5′‐triphosphate (CTP) and 9NH_2_‐Sia (**5**)^[^ ^17^ ^]^ at pH 8.5, forming 9NH_2_‐Sia*α*2,3‐LacNAc*β*ProN_3_ (**6**) in 69% yield. The reaction of trisaccharide **6** with TCC‐NHS (**7**) yielded ^TCC^Sia*α*2−3LacNAc*β*ProN_3_ (**8**) in 85% yield. The azide of **8** was reduced via catalytic hydrogenolysis, producing ^TCC^Sia*α*2−3LacNAc*β*ProNH_2_ (**9**), which was treated with DBCO‐NHS (**10**) in tetrahydrofuran/water/triethylamine, affording ^TCC^Sia*α*2−3LacNAc*β*Pro‐DBCO (^TCC^Sia‐LacNAc‐DBCO, **2**) in 56% yield (**Scheme** **2**).

### 2.3 Synthesis and Colocalization Studies of ^TCC^Sia‐Ace‐Dex‐OVA^FITC^‐Rd

**Scheme 3:**
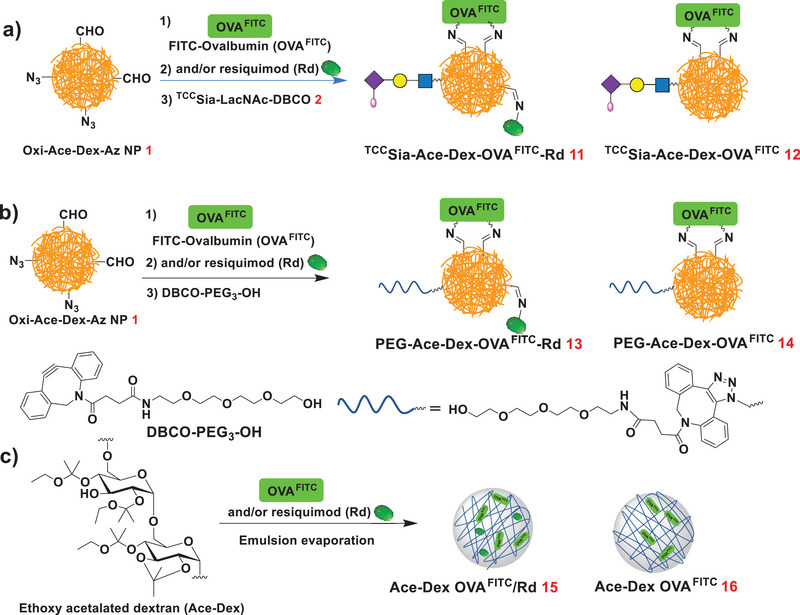
a) Synthesis of ^TCC^Sia‐Ace‐Dex‐OVA^FITC^‐Rd (**11**) and ^TCC^Sia‐Ace‐Dex‐OVA^FITC^ (**12**). b) Synthesis of PEG‐Ace‐Dex‐OVA^FITC^‐Rd (**13**) and PEG‐Ace‐Dex‐OVA^FITC^ (**14**). c) Synthesis of Ace‐Dex OVA^FITC^/Rd (**15**) and Ace‐Dex OVA^FITC^ (**16**).

The first step in CTL activation is the uptake of vaccines by APCs. We first need to understand the distribution of the ^TCC^Sia‐Ace‐Dex‐PA‐Rd in APCs. To visualize OVA in APCs, fluorescein isothiocyanate‐labeled OVA (OVA^FITC^) was used instead of OVA. OVA^FITC^ was added to a solution of NP **1** in PBS (pH 7.4). After 5 h of coupling, Rd and **2** were added to the solution overnight. The resulting NPs were washed with water and lyophilized to obtain ^TCC^Sia‐Ace‐Dex‐OVA^FITC^‐Rd (**11**, **Scheme** **3**a). To confirm the role of Rd in the Ace‐Dex system for CTL induction, ^TCC^Sia‐Ace‐Dex‐OVA^FITC^ (**12**) without Rd was also produced (Scheme 3a). In parallel, we synthesized PEG‐Ace‐Dex‐OVA^FITC^‐Rd (**13**) and PEG‐Ace‐Dex‐OVA^FITC^ (**14**) bearing polyethylene glycol (PEG_3_) (Scheme 3b), which cannot bind to CD169. To benchmark the Schiff‐base chemistry performance in the Ace‐Dex system for OVA^FITC^ delivery, we also synthesized Ace‐Dex OVA^FITC^/Rd (**15**) and Ace‐Dex OVA^FITC^ (**16**) by a noncovalent encapsulation method (Scheme 3c).

Next, colocalization studies were performed using CD169‐overexpressing mouse bone marrow‐derived macrophages (CD169^+^ BMMs), which is an important class of APCs. To prepare CD169^+^ BMMs, bone marrow cells were isolated from C57BL/6 mice and cocultured with macrophage‐colony stimulating factor (M‐CSF). After 7 days of culture, CD169 expression was induced by lipopolysaccharide stimulation. The levels of CD169 on BMMs were confirmed by staining with FITC‐labeled anti‐mouse CD169 antibody and PE‐labeled anti‐mouse F4/80 recombinant antibody.

**Figure 2:**
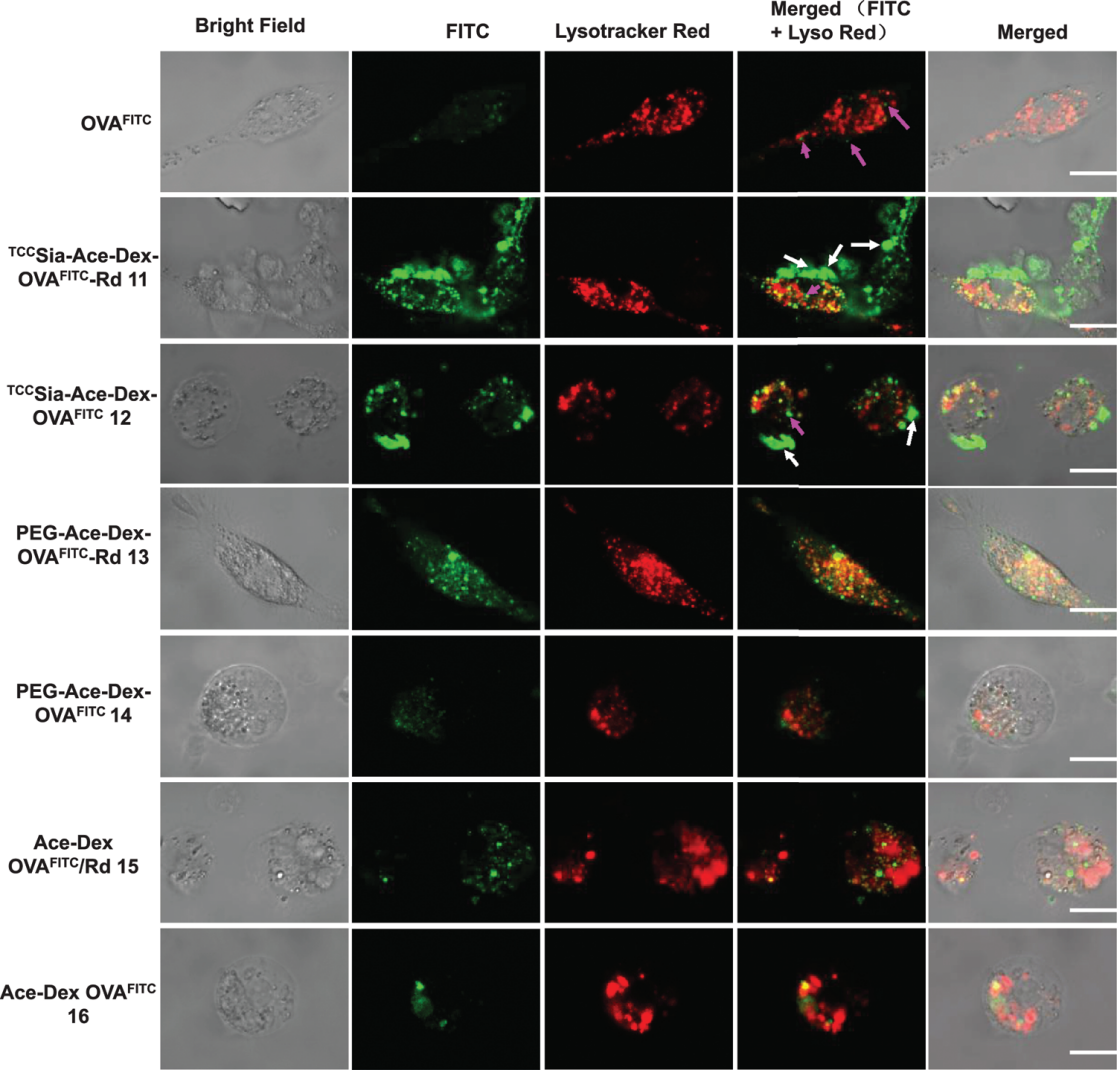
Colocalization of free OVA^FITC^, ^TCC^Sia‐Ace‐Dex‐OVA^FITC^‐Rd (**11**), ^TCC^Sia‐Ace‐Dex‐OVA^FITC^ (**12**), PEG‐Ace‐Dex‐OVA^FITC^‐Rd (**13**), PEG‐Ace‐Dex‐OVA^FITC^ (**14**), Ace‐Dex OVA^FITC^/Rd (**15**), and Ace‐Dex OVA^FITC^ (**16**) within CD169^+^ bone marrow‐derived macrophages (BMMs) upon incubation with the nanoparticles (NPs; containing the same amount of OVA^FITC^, 10 µg) for 6 h. The intracellular FITC signals were merged with Lysotracker (Lyso) Red, and the colocalization was shown in yellow. Scale bars: 10 µm.

With CD169^+^ BMMs in hand, colocalization studies were performed. The BMMs were incubated with OVA^FITC^ or **11**−**16** (respectively) and then imaged by a confocal fluorescence microscope. As shown in **Figure** **2**, when the BMMs were incubated with OVA^FITC^, only some faint green fluorescence (pink arrows) was seen in the BMMs, indicating that the uptake of OVA^FITC^ by BMMs was inefficient. No noticeable increase in green fluorescence was found in the BMMs incubated with **14**. In contrast, the cells treated with **12** showed apparent green fluorescence on the cell surface (white arrows), which was caused by the binding of ^TCC^Sia‐LacNAc on NPs to the CD169 on BMMs. In addition, the uptake efficiency of **13** by BMMs was significantly higher than that of **14** (Figure 2), indicating that Rd effectively activated macrophages. Notably, the uptake of **13** by BMMs was markedly greater than that of **15**, highlighting the importance of Schiff base chemistry for antigen and adjuvant delivery. The importance of Rd is also reflected in **11**, which exhibits a more efficient protein delivery than **12**. Interestingly, stronger green fluorescence was observed on the surface (white arrows) and interior of the BMMs incubated with **11**. This indicated that the ^TCC^Sia‐Ace‐Dex‐PA‐Rd platform could lead to an efficient display of antigens, highlighting the great potential of the platform to induce powerful CTLs.

### 2.4 Synthesis and Immunological Evaluation of ^TCC^Sia‐Ace‐Dex‐OVA‐Rd

**Scheme 4:**
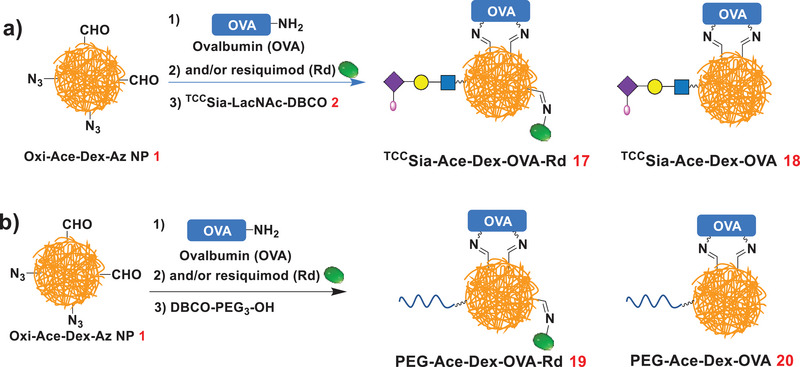
a) Synthesis of ^TCC^Sia‐Ace‐Dex‐OVA‐Rd (**17**) and ^TCC^Sia‐Ace‐Dex‐OVA (**18**). b) Synthesis of PEG‐Ace‐Dex‐OVA‐Rd (**19**) and PEG‐Ace‐Dex‐OVA (**20**).

After confirming that ^TCC^Sia‐Ace‐Dex‐OVA^FITC^‐Rd can efficiently enter APCs, we next prepared OVA‐based vaccines using this platform. ^TCC^Sia‐Ace‐Dex‐OVA‐Rd (**17**) was synthesized by coupling NP **1** sequentially with OVA, Rd, and **2** (**Scheme** **4**a). In parallel, ^TCC^Sia‐Ace‐Dex‐OVA (**18**), PEG‐Ace‐Dex‐OVA‐Rd (**19**), and PEG‐Ace‐Dex‐OVA (**20**) were also prepared (Scheme 4). As determined by a Bradford assay, the average amount of OVA in **17**−**20** was 76, 87, 67, and 79 µg of protein per mg NP, respectively (Table S1, Supporting Information). As determined by a UV absorption assay at 345 nm, the average content of ^TCC^Sia‐LacNAc in **17** and **18** was 67 and 70 µg of ^TCC^Sia‐LacNAc per mg NP, respectively. The average amounts of Rd by **17** and **19** were quantified to be 62 and 60 µg of Rd per mg NP (respectively) by UV absorption measurement at 321 nm (Table S1, Supporting Information).

**Figure 3:**
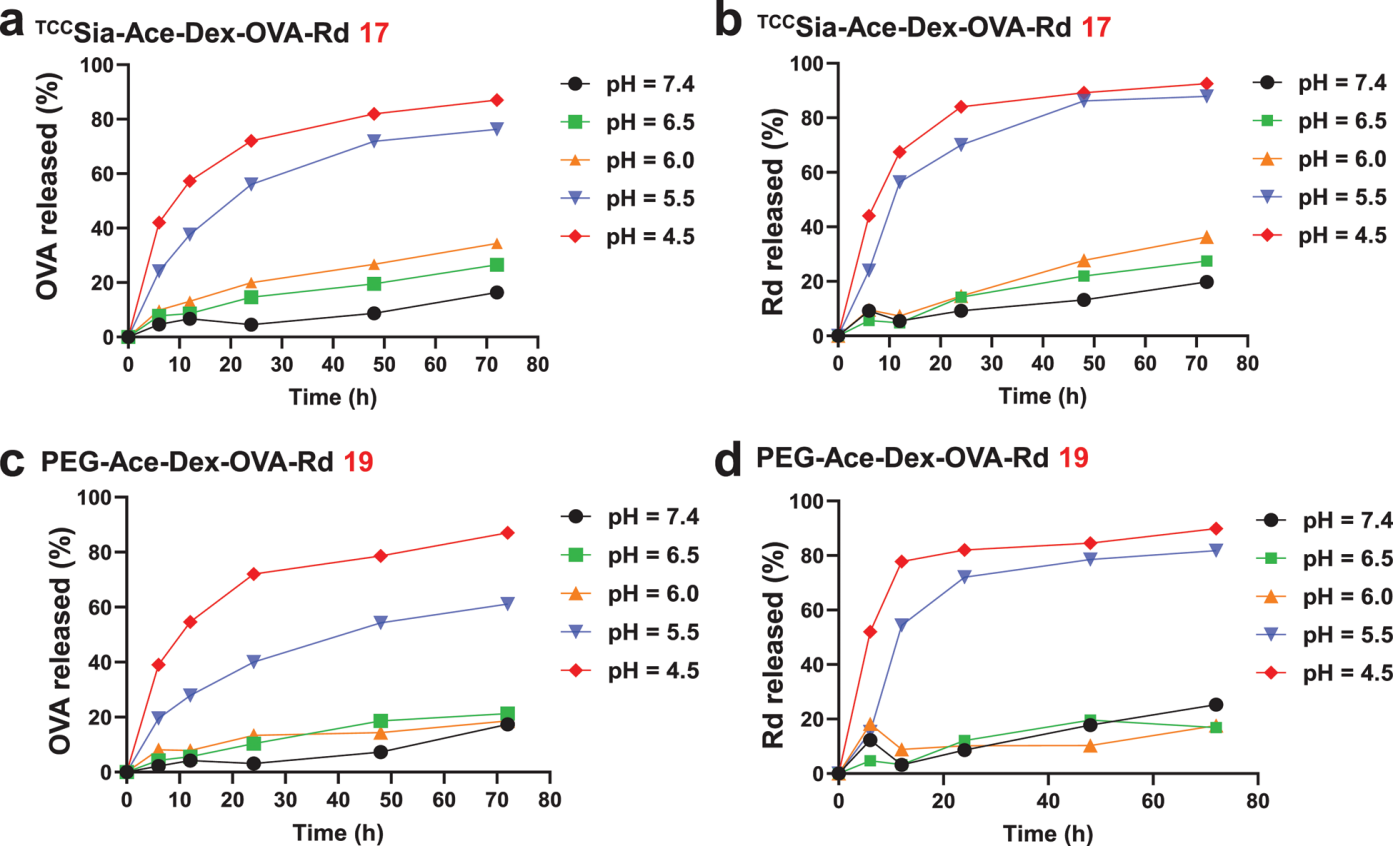
The pH‐dependent release profiles of ovalbumin (OVA) and Rd from a,b) ^TCC^Sia‐Ace‐Dex‐OVA‐Rd (**17**) and c,d) PEG‐Ace‐Dex‐OVA‐Rd (**19**).

With **17** and **19** in hand, we next tested the release rates of conjugated OVA and Rd in these NPs by treatment of the NPs in PBS at 37 °C with pH values of 7.4, 6.5, 6.0, 5.5, and 4.5. As shown in **Figure** **3**, when spiked into PBS at pH 6.0−7.4, the spontaneous release of OVA and Rd from **17** and **19** was slow. In contrast, their release rates increased significantly at pH 4.5 and 5.5 (Figure 3). This suggested that OVA and Rd can be released in acidic environments, including in lysosomes and endosomes.

**Figure 4:**
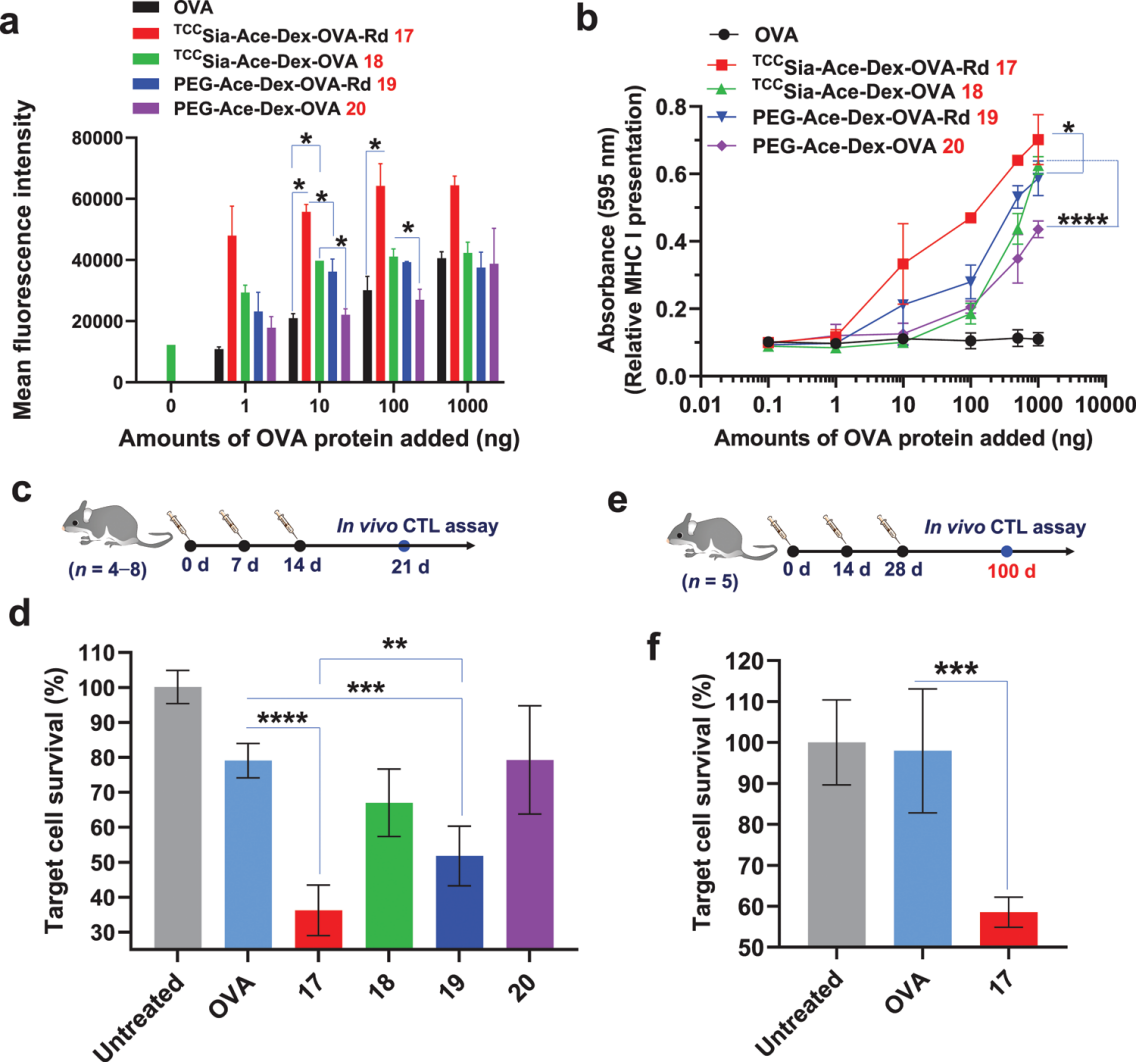
a) Detection of OVA_257−264_ presented by MHC‐I of the bone marrow‐derived macrophages (BMMs; CD169^+^). The BMMs (5 × 10^5^) were incubated with either free OVA, ^TCC^Sia‐Ace‐Dex‐OVA‐Rd (**17**), ^TCC^Sia‐Ace‐Dex‐OVA (**18**), PEG‐Ace‐Dex‐OVA‐Rd (**19**), and PEG‐Ace‐Dex‐OVA (**20**) for 24 h. The resulting cells were spiked with PE‐labeled anti‐mouse H‐2K^b^/SIINFEKL antibody for 30 min and analyzed by flow cytometry. b) MHC‐I antigen presentation by the BMMs. In vitro cytotoxic T‐lymphocyte (CTL) activation study of B3Z cells cocultured with the BMMs following incubation with free OVA and **17−20**, respectively. The error bars show the SD of three replicates. c,d) In vivo CTL activities. Mice were immunized weekly with free OVA and **17−20**, respectively (*n* = 8 mice for the untreated group, *n* = 4 mice per group for OVA, **18**, and **20** injections, *n* = 6 mice per group for **17** and **19** injections). On day 21, after three vaccinations, a 1:1 of CFSE^hi^OVA_257−264_^+^ and CFSE^lo^OVA_257−264_^−^ splenocytes was injected into the immunized and nontreated mice, respectively. After 24 h, their splenocytes were analyzed by flow cytometry. e,f) Persistence of in vivo CTL activities. Mice were immunized subcutaneously by three injections of free OVA or **17** on days 0, 14, and 28, respectively. On day 100, in vivo CTL assay was performed (*n* = 5 mice per group). The *p* values are analyzed by a two‐tailed unpaired Student's *t*‐test (a,d,f) or a two‐way ANOVA Bonferroni posttest (b) with GraphPad Prism 8. *p < 0.05, **p < 0.01, ***p < 0.001, ****p < 0.0001.

Activating APCs is a critical step in generating CTLs. We next assessed the effect of **17**−**20** on CTL activation. We first tested the presentation of OVA on major histocompatibility complex class I (MHC‑I) by the BMMs (CD169^+^). The BMMs were incubated with free OVA or **17**−**20**, which contained 0−1 µg of OVA. The incubated cells were stained with phycoerythrin (PE)‐labeled anti‐mouse H‐2K^b^/SIINFEKL antibody and then analyzed by flow cytometry. It was found that incubation of BMMs with **18** resulted in PE fluorescence intensities of the BMMs that were much increased compared to those of **20** (**Figure** **4**a). This validates the effectiveness of ^TCC^Sia‐LacNAc conjugation for enhancing MHC‐I antigen cross‐presentation of OVA peptide (OVA_257−264_, SIINFEKL). Furthermore, incubation of the BMMs with **17** led to a significant increase in PE fluorescence compared to **19** treatments (Figure 4a), further validating the importance of ^TCC^Sia‐LacNAc. To determine whether OVA_257−264_‐presented BMMs can be recognized by CTLs, B3Z assays^[^ ^18^ ^]^ were subsequently performed. The BMMs were incubated with free OVA or **17**−**20**. The resulting OVA_257−264_‐loaded BMMs were then cocultured with B3Z cells. As shown in Figure 4b, **17−20** (respectively) incubation led to more robust activation of B3Z cells than free OVA incubation. A comparison between **17** and **19** showed that **17** enhanced B3Z cell activation, indicating that **17** is superior to **19** in inducing OVA_257−264_‐specific CTLs.

After demonstrating their abilities to activate CTLs in vitro, in vivo CTL activation was evaluated. C57BL/6 mice were vaccinated with free OVA or **17−20**, by three weekly injections. The induced antigen‐specific T lymphocyte activities were evaluated by an in vivo CTL study using a carboxyfluorescein succinimidyl ester (CFSE) assay.^[^ ^19^ ^]^ Syngeneic splenocytes were used as targets for the in vivo CTL assay. As shown in Figure 4d, vaccinating these mice with **17** by the subcutaneous route produced more potent OVA‐specific CTLs than those of **19**, resulting in a higher population of OVA_257−264_ pulsed target cells were lysed, highlighting the advantages of conjugating ^TCC^Sia‐LacNAc with Ace‐Dex NPs for enhancing CTL activation in vivo. Subsequently, we also tested the persistence of CTL responses induced by the NPs. Intriguingly, after three vaccinations with **17** on days 0, 14, and 28, robust OVA‐specific CTL activities can be detected in the spleen on day 100 (Figure 4f), indicating that long‐lasting OVA‐specific CTL responses were elicited.

**Figure 5:**
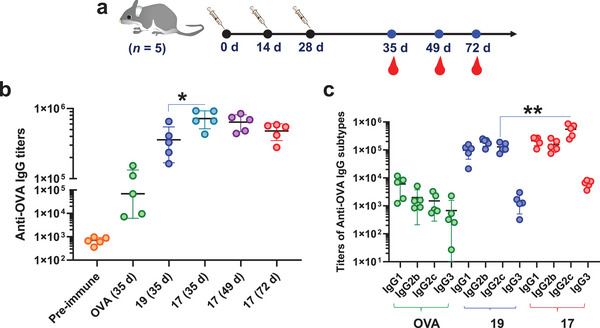
a,b) Anti‐OVA IgG antibody titers in mice immunized with free OVA, ^TCC^Sia‐Ace‐Dex‐OVA‐Rd (**17**), and PEG‐Ace‐Dex‐OVA‐Rd (**19**), respectively. c) IgG subtype titers in mice immunized with free OVA, **19** or **17**. Each symbol represents one mouse (*n* = 5 mice for each group). A two‐tailed unpaired Student's *t*‐test determined the *p* values with GraphPad Prism 8. *p < 0.05, **p < 0.01.

In addition to assessing CTL immunity, we also evaluated the induced humoral immunity of the NPs. On days 0, 14, and 28, C57BL/6 mice were immunized with sterile solutions of OVA, **17**, and **19**, respectively. Serum was collected on days −1, 35, 49, and 72 to determine antibody titers by ELISA (**Figure** **5**a). The results showed that at day 35, free OVA, **19**, and **17** induced mean IgG titers in mice of 69 553, 357 726, and 720 080, respectively (Figure 5b). The mean IgG titer generated by **19** is five times that of free OVA‐induced IgG, indicating that incorporating OVA and Rd on the Ace‐Dex carrier can efficiently activate B cells, resulting in high IgG antibodies. In addition, surprisingly, the mean IgG titer induced by **17** was 10 times higher than that induced by free OVA, demonstrating that the efficient uptake of nanomaterials by macrophages can induce stronger humoral immunity. Humoral responses can be enhanced by targeting the mannose receptors of APCs, including macrophages and dendritic cells.^[^ ^20^ ^]^ However, to our knowledge, targeting CD169 on macrophages with high‐affinity glycan ligands to enhance IgG production has not been reported. Our findings extend the application of ^TCC^Sia‐LacNAc.

Given the superiority of **17**, we next tested the persistence of antibody production. The results showed that the IgG induced by **17** still maintained high titers after 72 days, with an average titer of 479 329 (Figure 5b), indicating that **17** can induce long‐lasting IgG. IgG subtype analysis showed that the antibodies induced by free OVA were mainly IgG1, while **19** and **17** induced higher levels of IgG1, IgG2b, and IgG2c (Figure 5c), highlighting that conjugating OVA and Rd in Ace‐Dex NPs can be efficient in triggering Th1 and Th2 humoral responses. More importantly, **17** induced significantly higher IgG2c levels than those of **19**, suggesting that macrophage targeting contributes to a stronger Th1‐biased antibody response. Together, the result indicated that **17** induced robust and durable IgG responses in addition to strong CTL immunity in mice.

### 2.5 Tumor Challenge Study

**Figure 6:**
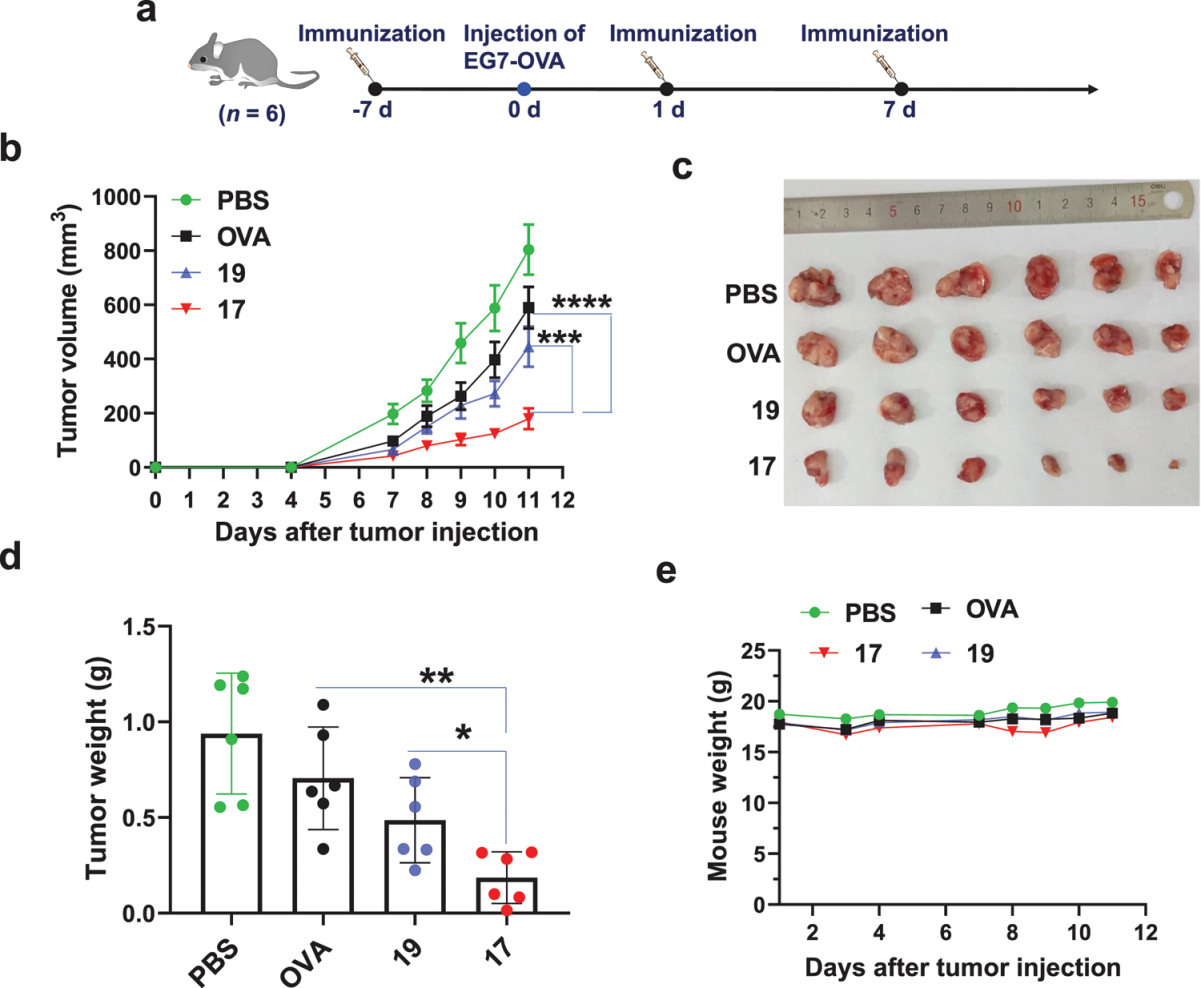
EG7‐OVA tumor growth. a) On day 0, C57BL/6 female mice were injected with EG7‐OVA cells (1 × 10^6^). On days −7, 1, and 7, a total of three subcutaneous injections with PBS, free OVA, ^TCC^Sia‐Ace‐Dex‐OVA‐Rd (**17**) or PEG‐Ace‐Dex‐OVA‐Rd (**19**) with the same amounts of OVA (100 µg) were given. b) Growth curves of tumors in mice were collected. On day 11, the mice were sacrificed by anesthesia. c) Photographs of the dissected tumors were taken, and d) the dissected tumors were weighed. e) The average body weight of tumor‐bearing mice was monitored over time. The error bars represent the SEM (b) or SD (d) of six mice for each group. The *p* value was obtained by two‐way ANOVA Bonferroni posttest (b) or a two‐tailed *t*‐test (d) with GraphPad Prism 8. *p < 0.05, **p < 0.01, ***p < 0.001, ****p < 0.0001.

With the excellent CTL and IgG responses elicited by ^TCC^Sia‐Ace‐Dex‐OVA‐Rd (**17**), we evaluated its ability to protect tumors. Seven days before tumor implantation (day −7), the mice were immunized with PBS, free OVA, **17** or **19**. Seven days after the first immunization (day 0), mice were subcutaneously implanted with EG7‐OVA tumor cells, which are model cells that express OVA. One and seven days (days 1 and 7) after the EG7‐OVA cells were injected, the mice were administered two more vaccinations with PBS, free OVA, **17** or **19**. The growth of the tumor was monitored daily. As shown in **Figure** **6**b–d, compared to the mock groups that received **19** and **17** injections, the free OVA vaccination was ineffective in slowing tumor growth. Compared with **19**, **17** drastically reduced tumor growth (Figure 6b–d), highlighting the importance of ^TCC^Sia‐LacNAc NPs in enhancing CTL and humoral responses against tumors. No significant weight loss was observed in the mice (Figure 6e), implying that **17** was highly biocompatible despite the pronounced tumoricidal effects. No noticeable side effects were observed in the **17**‐immunized mice through histological analysis (Figure S2, Supporting Information), confirming the potency and safety of **17** for future clinical translation.

### 2.6 Synthesis and Immunological Evaluation of the ^TCC^Sia‐Ace‐Dex‐RBD‐Rd

The COVID pandemic caused by SARS‐CoV‐2 is still seriously afflicting the world. According to a report by World Health Organization (WHO), as of 6 October 2022, there were 616 951 418 confirmed cases of COVID‐19 globally and 6 530 281 deaths.^[^ ^21^ ^]^ In response to SARS‐CoV‐2 infection, many vaccines have been developed to prevent SARS‐CoV‐2, including mRNA vaccines (Pfizer‐BioNTech BNT162b2 and Moderna mRNA‐1273),^[^ ^22^ ^]^ recombinant protein vaccines (Novavax NVX‐CoV2373 and ZF2001),^[^ ^5^, ^7^ ^]^ viral vector vaccines (AstraZeneca ChAdOx1 nCoV‐19 and Janssen Ad26.COV2.S),^[^ ^23^ ^]^ and inactivated vaccines (Sinovac [CoronaVac] and Sinopharm).^[^ ^24^ ^]^

While vaccination of current vaccines is effective against the prototype strain of SARS‐CoV‐2, the effectiveness of current vaccines has been significantly reduced against VOCs of SARS‐CoV‐2, including Omicron (BA. 1 to BA. 5).^[^ ^2b,c^ ^]^ Furthermore, current vaccines cause common side effects (headache and fatigue, etc.),^[^ ^25^ ^]^ as well as rare side effects (blood clots and cardiac injury, etc.).^[^ ^26^ ^]^ This underscores the need to continuously improve vaccines against SARS‐CoV‐2 and its VOCs.

The immunogens of current SARS‐CoV‐2 protein‐based vaccines (Novavax NVX‐CoV2373 and ZF2001) are based on the spike (S) protein of SARS‐CoV‐2 or its RBD region.^[^ ^4^, ^5^, ^7^ ^]^ Compared with S protein vaccines, RBD vaccines are safer because they avoid the production of many unnecessary antibodies that can cause side effects.^[^ ^27^ ^]^ However, the antibodies produced by RBD vaccines exhibit a reduced ability to neutralize SARS‐CoV‐2 variants, especially against Omicron. Encouragingly, booster injections of vaccines (BNT162b2 and mRNA‐1273) increase the vaccine effectiveness against Omicron.^[^ ^28^ ^]^ This suggests that it can increase efficacy against variants if stronger anti‐RBD antibodies can be induced.

**Figure 7:**
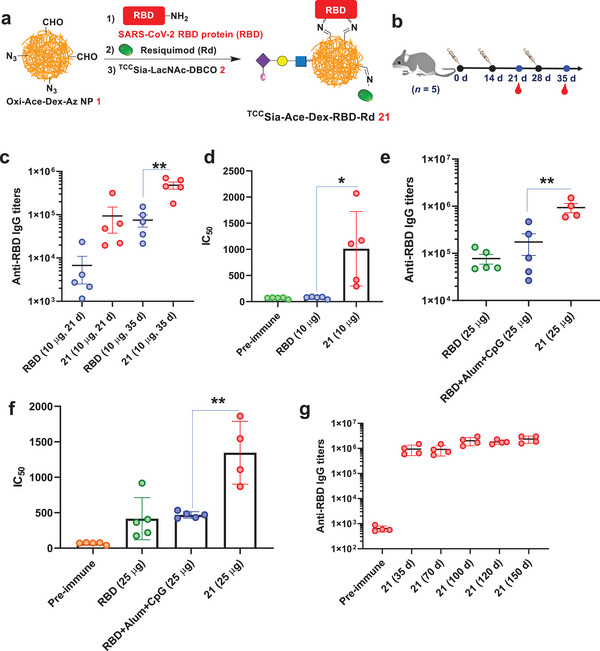
a) Synthesis of ^TCC^Sia‐Ace‐Dex‐RBD‐Rd (**21)**. b) Immunization of mice with free receptor‐binding domain (RBD) (10 µg) and **21** (with 10 µg RBD in the nanoparticles, NPs), respectively. c) Titers of anti‐RBD IgG from mice immunized with free RBD and **21**, respectively. d) IC_50_ values of RBD binding to hACE2 by antibodies elicited by free RBD and **21** showed **21**‐induced antibodies significantly blocked the SARS‐CoV‐2 RBD binding to hACE2. e–g) Immunization of mice with free RBD (25 µg), **21** (with 25 µg RBD in the NPs), or RBD+Alum+CpG (with 25 µg RBD). e) Titers of anti‐RBD IgG antibodies from mice immunized with free RBD, **21**, or RBD+Alum+CpG. f) IC_50_ determination of RBD binding to hACE2 by IgG elicited by free RBD, RBD+Alum+CpG and **21** showed **21** induced IgG effectively blocked SARS‐CoV‐2 RBD binding to hACE2. g) Persistence of antibody responses induced by **21**. Each symbol represents one mouse (*n* = 4−5 mice for each group). A two‐tailed unpaired Student's *t*‐test determined the *p* values with GraphPad Prism 8. *p < 0.05, **p < 0.01.

In the studies mentioned above, we completed the synthetic and immunological evaluation of ^TCC^Sia‐Ace‐Dex‐OVA‐Rd, demonstrating its ability to generate robust CTL and humoral immunity. Inspired by this, we synthesized ^TCC^Sia‐Ace‐Dex‐RBD‐Rd (**21**) by sequentially conjugating NP **1** with SARS‐CoV‐2 recombinant RBD protein (aa 321−591), Rd and ^TCC^Sia‐LacNAc‐DBCO **2** (**Figure** **7**a). The amount of RBD in **21** was determined by a Bradford assay, then the amounts of ^TCC^Sia‐LacNAc and Rd were determined by UV absorption measurements, as shown in Table S2 (Supporting Information).

After synthesizing and characterizing the vaccine, we assessed humoral immunity by **21**. C57BL/6 female mice were immunized three times on days 0, 14, and 28. Serum was collected before and after immunization for subsequent experiments (Figure 7b). It was found that on day 35, the mean IgG titers induced by free RBD (10 µg) and **21** (with 10 µg RBD) in mice were 75 400 and 478 736, respectively (Figure 7c). In addition, we also examined IC_50_, which reflects the effect of serum antibodies on blocking the interaction between RBD and the angiotensin‐converting enzyme 2 (hACE2). The higher the IC_50_ value is, the higher the neutralizing antibody level in the serum. The mean IC_50_ values of sera from free RBD and **21** immunized mice were 76 and 1011, respectively (Figure 7d). This indicated that postimmunization serum antibodies produced by **21** significantly blocked the RBD‐hACE2 interaction, while free RBD‐induced serum was less effective.

We also examined the effect of the injected amount of RBD on antibody production. As a control, we added a reported SARS‐CoV‐2 vaccine (RBD+Alum+CpG).^[^ ^6a^ ^]^ C57BL/6 mice were immunized on days 0, 14, and 28 with free RBD (25 µg), RBD+Alum+CpG (with 25 µg RBD), and **21** (with 25 µg RBD in the NPs), respectively. As shown in Figure 7e, after three immunizations, the average titer of IgG induced by RBD+Alum+CpG was 174 435 on day 35, which was twice that induced by free RBD. Interestingly, **21** produced high titers of IgG in mice, with a mean IgG titer of 935 927; this was fivefold higher than that of RBD+Alum+CpG‐induced IgG titers. IgG subtype analysis found that vaccination with **21** led to high titers of IgG1, IgG2c, and IgG3 (Figure S4, Supporting Information), highlighting that **21** induced both Th1 and Th2 humoral responses. We also tested whether these sera could block the interaction of RBD and hACE2. The results showed that the mean IC_50_ values of sera from free RBD, RBD+Alum+CpG, and **21** immunized mice were 416, 469, and 1346, respectively (Figure 7f). This indicated that the serum antibodies produced by **21** significantly blocked the RBD‐hACE2 interaction, while free RBD and RBD+Alum+CpG‐induced antibodies were less effective in blocking the RBD‐hACE2 interaction. More importantly, IgG induced by **21** maintained high titers after 150 days, with a mean titer of 2 358 471 (Figure 7g), indicating that **21** can induce long‐lasting IgG. These results indicated that **21** induced robust and long‐lasting RBD‐neutralizing antibodies in mice.

**Figure 8:**
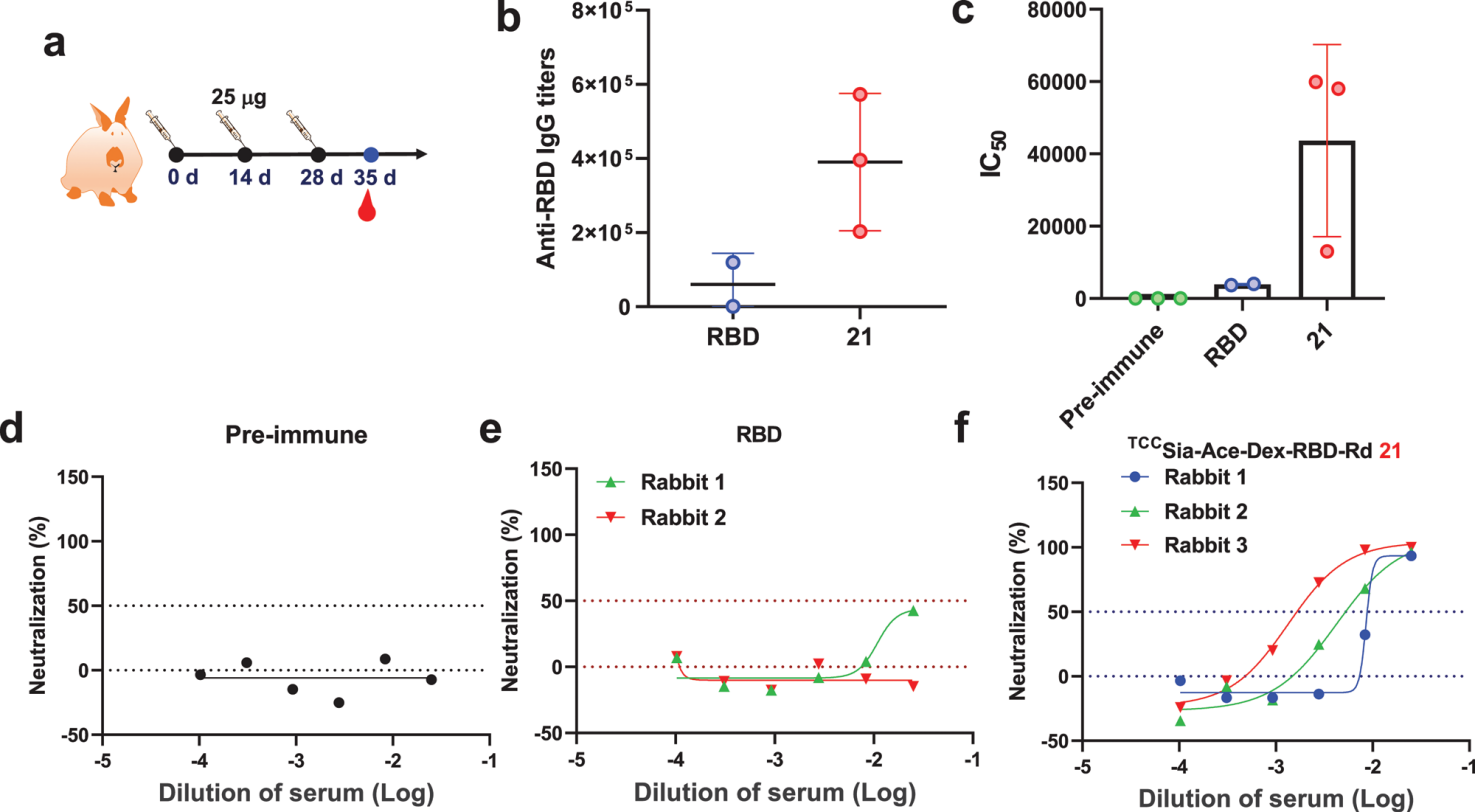
a) Immunization of rabbits with free receptor‐binding domain (RBD; 25 µg) and **21** (with 25 µg RBD), respectively. b) Titers of anti‐RBD IgG from rabbits immunized with free RBD and **21**, respectively. c) IC_50_ values of RBD binding to hACE2 by IgG elicited by free RBD and **21** from rabbits showed **21**‐induced IgG effectively blocked SARS‐CoV‐2 RBD binding to hACE2. b,c) Each symbol represents one rabbit. d–f) Authentic SARS‐CoV‐2 neutralization test showed that **21** postimmune rabbit sera effectively neutralized authentic SARS‐CoV‐2 (WIV04 strain) virus infection of Vero E6 cells.

We next evaluated the effectiveness and safety of **21** in rabbits. Rabbits were immunized three times on days 0, 14, and 28. Serum was collected before and after immunization for subsequent experiments (**Figure** **8**a), and their organs were isolated for histological analysis. The results showed that on day 35, immunization with free RBD produced a mean titer of anti‐RBD IgG of 60 389 in rabbits. In contrast, immunization of rabbits (1−3) with **21** produced higher IgG titers, with 202 672, 395 419, and 573 021 titers, respectively (Figure 8b). Furthermore, the mean IC_50_ value of anti‐RBD IgG produced by free RBD in rabbits for blocking the interaction between RBD and hACE2 was 3878. Interestingly, the IC_50_ values of anti‐RBD IgG produced by **21** in rabbits (1−3) for inhibition of RBD‐hACE2 interaction were 13 002, 58 064, and 59 896, respectively (Figure 8c). These results indicate that **21** induces higher levels of RBD‐neutralizing antibodies in rabbits than that of free RBD.

To further confirm whether the induced RBD antibodies could neutralize SARS‐CoV‐2, we performed live virus neutralization tests using the SARS‐CoV‐2 WIV04 strain.^[^ ^29^ ^]^ The results showed preimmune rabbit serum antibodies could not neutralize authentic SARS‐CoV‐2 infection of Vero E6 cells (Figure 8d). Rabbit sera immunized with free RBD insufficiently neutralized authentic SARS‐CoV‐2 virus (Figure 8e). Interestingly, immunization of rabbits (1−3) with **21** produced high RBD‐neutralizing antibodies with IC_50_ values of 117, 237, and 747 (Figure 8f) against infection with authentic SARS‐CoV‐2 virus. Furthermore, we collected and sliced rabbit organs after three immunizations for hematoxylin and eosin (H&E) staining. The results showed that vaccination with **21** had no observable side effects on rabbit organs, including the heart, liver, spleen, lung, and kidney (Figure S3, Supporting Information), confirming the safety of **21** for future clinical translation.

### 2.7 Synthesis and Immunological Evaluation of the ^TCC^Sia‐Ace‐Dex‐N‐Rd

In addition to the frequently mutated S or RBD protein, other structurally conserved SARS‐CoV‐2 proteins should be actively explored as immunogens to create a universal vaccine against SARS‐CoV‐2 and its VOCs. The nucleocapsid (N) protein is highly conserved in coronaviruses, including SARS‐CoV‐1, SARS‐CoV‐2, and its VOCs (BA.1 to BA.5).^[^ ^2c^ ^]^ In addition, the N protein is abundantly expressed during viral infection.^[^ ^30^ ^]^ There are an estimated 100 S copies and 1000 N copies per virion. More importantly, the N protein contains multiple T‐cell epitopes that can lead to robust T‐cell immunity.

**Scheme 5:**
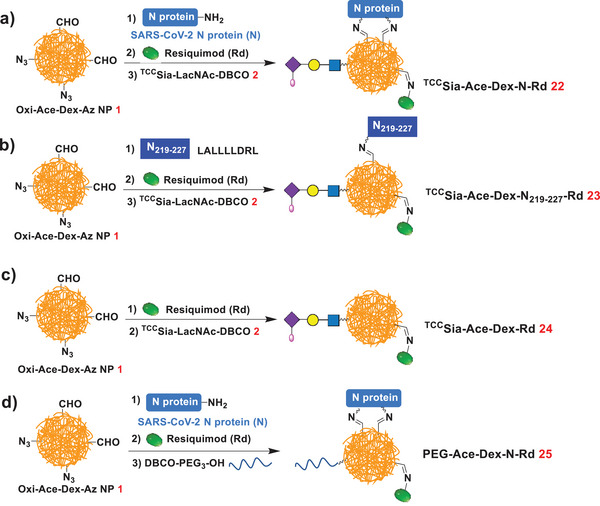
Synthesis of a) ^TCC^Sia‐Ace‐Dex‐N‐Rd (**22**), b) ^TCC^Sia‐Ace‐Dex‐N_219−227_‐Rd (**23**), c) ^TCC^Sia‐Ace‐Dex‐Rd (**24**), and d) PEG‐Ace‐Dex‐N‐Rd (**25**).

Given the advantages of SARS‐CoV‐2 N protein mentioned above, we designed ^TCC^Sia‐Ace‐Dex‐N‐Rd (**22**) as a universal vaccine. As shown in **Scheme** **5**a, NP **22** was synthesized by sequentially linking SARS‐CoV‐2 N protein (N),^[^ ^31^ ^]^ Rd, and **2** to Oxi‐Ace‐Dex‐Az NPs (**1**). Notably, in our recent study,^[^ ^10a^ ^]^ a CTL epitope from the N protein (N_219−227_) with the sequence of LALLLLDRL was identified. In parallel, we conjugated NP **1** with N_219−227_, Rd and **2** to obtain ^TCC^Sia‐Ace‐Dex‐N_219−227_‐Rd **23** (Scheme 5b). In addition, we synthesized PEG‐Ace‐Dex‐N‐Rd (**25**, Scheme 5d) with PEG_3_ as a negative control in the absence of ^TCC^Sia‐LacNAc. The protein content of NPs was determined by a Bradford assay. The peptide content was calculated by HPLC analysis, and the amounts of ^TCC^Sia‐LacNAc and Rd were determined by UV absorption assays (Table S3, Supporting Information).

**Figure 9:**
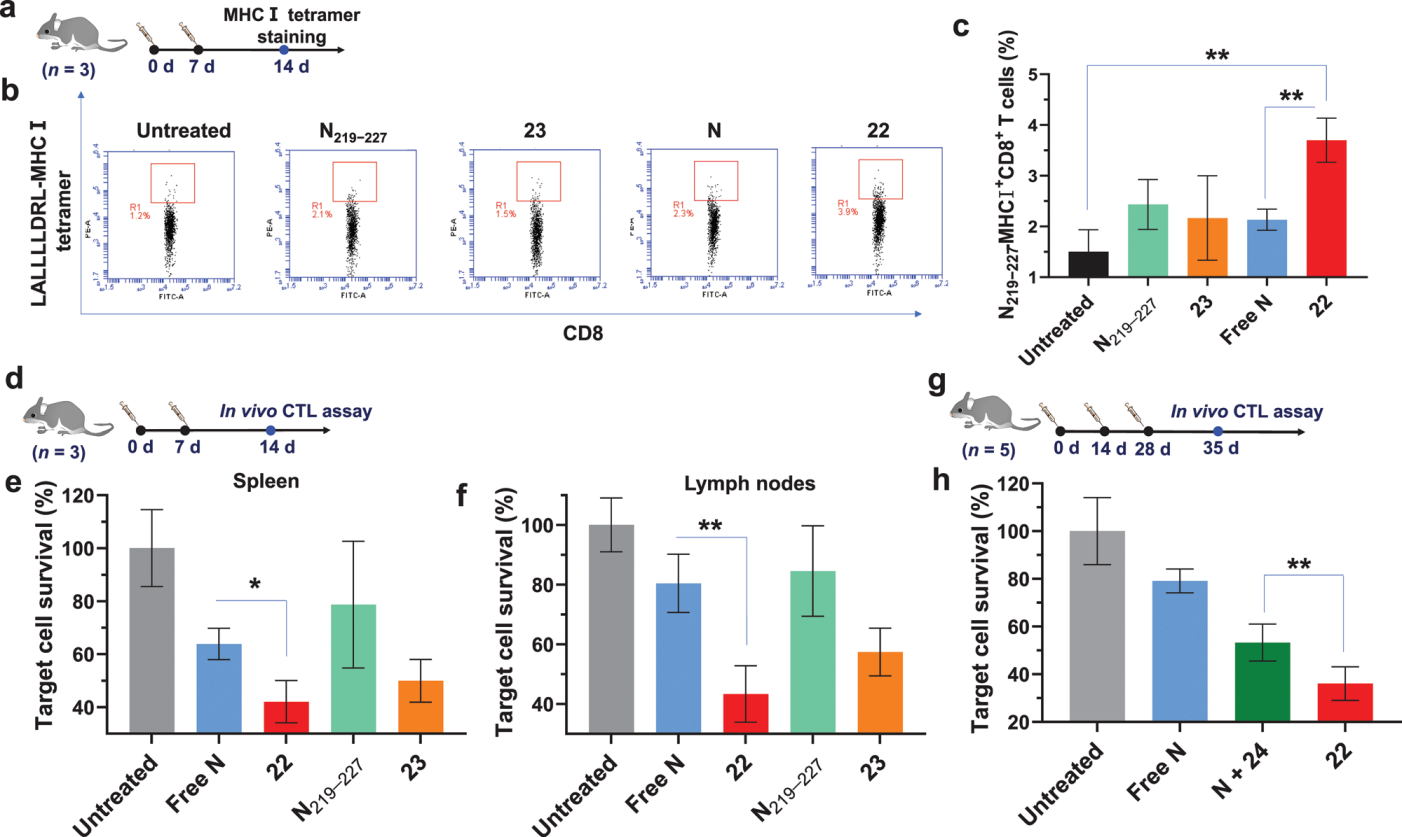
a–c) Vaccination of mice with ^TCC^Sia‐Ace‐Dex‐N‐Rd (**22**) induced N_219−227_ specific cytotoxic T‐lymphocytes (CTLs) in their splenocytes. C57BL/6 mice were subcutaneously immunized on days 0 and 7 with free N (50 µg), N_219−227_ (equivalent to the amount of N_219−227_ in 50 µg N), **22** (with 50 µg N), or **23** (with the same amount of N_219−227_ as the N_219−227_ treated group). 14‐day later, their spleens were collected for MHC‐I tetramer staining. The percentage of LALLLLDRL‐MHC‐I^+^CD8^+^ cells present in splenocytes was determined by staining splenocytes with PE‐conjugated LALLLLDRL‐MHC‐I tetramer prepared by QuickSwitch Custom MHC Tetramer Kit and FITC‐conjugated anti‐mouse CD8 antibody. d–f) In vivo CTL activities of free N, N_219−227_, **22** and **23**. By weekly injections, mice were immunized twice with free N (50 µg), N_219−227_ (equivalent to the amount of N_219−227_ in 50 µg N), **22** (with 50 µg N) or **23** (with the same amount of N_219−227_ as the N_219−227_ treated group), respectively, using nontreated mice as control (*n* = 3 mice per group). 14‐day later, a mixture of CFSE^hi^N_219−227_^+^ target cells and CFSE^lo^N_219−227_^−^ control cells with a ratio of 1:1 was injected into the immunized and nontreated mice, respectively. One day after injection, e) their splenocytes and f) lymph node cells were analyzed by flow cytometry. g,h) Mice were immunized three times with free N (50 µg), **22** (with 50 µg N) or N + **24** (with 50 µg N), respectively (*n* = 5 mice per group). 35‐day later, in vivo CTL assay was performed. A two‐tailed unpaired Student's *t*‐test determined the *p* values with GraphPad Prism 8. *p < 0.05, **p < 0.01.

With **22** and **23** in hand, the effects of these NPs on CTL activation were assessed. The generation of N peptide‐specific CTLs provides direct evidence that CTL is activated, and these CTLs can be detected by MHC‐I tetramer staining. To perform this study, N_219−227_ (LALLLLDRL), an identified CTL epitope from the SARS‐CoV‐2 N protein, was synthesized. On days 0 and 7, C57BL/6 mice were immunized with free N, **22**, N_219−227_, or **23**. Two weeks later, their spleens were isolated and transformed into a suspension for N_219−227_‐MHC‐I tetramer staining (**Figure** **9**a). Higher levels of N_219−227_‐specific CTLs (LALLLLDRL‐MHC‐I^+^CD8^+^ cells) were detected in the spleens of mice immunized with **22** (Figure 9b,c), which is direct evidence for the generation of N_219−227_‐specific CTLs. Furthermore, vaccination with **22** resulted in a marked upregulation of MHC‐I, CD86, CD8, and CD4 levels on mouse splenocytes (Figure S5, Supporting Information), which are critical for CTL activation. No detectable levels of serum IFN‐*γ* and TNF‐*α* were observed in the sera of vaccinated mice (Figure S6, Supporting Information), suggesting that **22** can induce strong anti‐SARS‐CoV‐2 CTL responses without causing a damaging cytokine storm.

Next, an in vivo CTL assay was performed to verify the killing effect of the generated CTLs. Mice were immunized weekly with free N, **22**, N_219−227_, and **23**, respectively. After 2 weeks, a mixture of CFSE^hi^‐labeled N_219−227_ pulsed (CFSE^hi^N_219−227_^+^) splenocytes and CFSE^lo^‐labeled control (CFSE^lo^N_219−227_^−^) splenocytes was injected into immunized and nontreated mice. After 24 h, the spleens and lymph nodes from these mice were isolated and transformed into a suspension for flow cytometry analysis. As shown in Figure 9e,f, NP **22** (or **23**) vaccination induced higher levels of N_219−227_‐specific CTLs than free N (or N_219−227_) immunization. Notably, **22** induced stronger N_219−227_ specific CTLs than **23**, indicating that N protein is more immunogenic than N peptide. Furthermore, N + ^TCC^Sia‐Ace‐Dex‐Rd (**24**) immunization resulted in higher levels of N_219−227_ pulsed target splenocyte killing compared to free N immunization, highlighting that **24** is a promising adjuvant that enhances CTL responses. As expected, ^TCC^Sia‐Ace‐Dex‐N‐Rd (**22**) generated a more robust N_219−227_‐specific CTL response than N + **24** (Figure 9h), underscoring the importance of covalently conjugating N to the NPs. This is because, compared to N + **24**, ^TCC^Sia‐Ace‐Dex‐N‐Rd can enter APCs (macrophages) more efficiently. This was confirmed by our findings that compared to N^FITC^ + **24**, ^TCC^Sia‐Ace‐Dex‐N^FITC^‐Rd was taken up by CD169^+^ BMMs more efficiently (Figure S7, Supporting Information).

**Figure 10:**
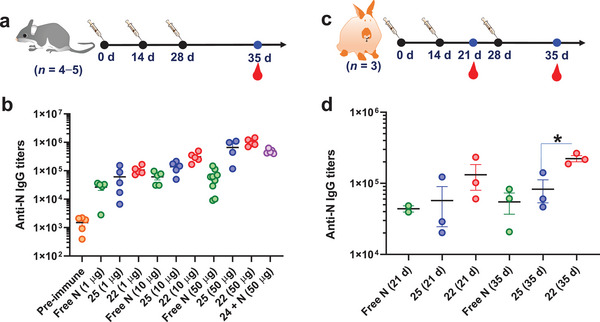
a,b) Titers of anti‐N IgG antibodies from mice immunized with free N, ^TCC^Sia‐Ace‐Dex‐N‐Rd (**22**), N + **24**, or PEG‐Ace‐Dex‐N‐Rd (**25**). Each symbol represents one mouse (*n* = 4−5 mice for each group). c,d) Titers of anti‐N IgG antibodies from rabbits immunized with free N, **22**, or **25**. Each symbol represents one rabbit (*n* = 3 rabbits for each group). A two‐tailed unpaired Student's *t*‐test determined the *p* values with GraphPad Prism 8. *p < 0.05.

The primary mechanism of N protein‐based vaccines is induced T‐cell immunity. Nevertheless, it has also been found that the levels of serum anti‐N antibodies are associated with protection against COVID‐19.^[^ ^32^ ^]^ Therefore, we assessed the humoral immunity elicited by ^TCC^Sia‐Ace‐Dex‐N‐Rd (**22**) in mice. C57BL/6 mice were immunized with sterile solutions of free N, **22**, **25**, and N + **24** on days 0, 14, and 28, respectively, and sera were collected on day 35 for ELISA (**Figure** **10**a). On day 35, the mean IgG titers induced by free N, **22** and **25** (with 50 µg N) in mice were 60 925, 1 019 055, and 658 323, respectively; the mean IgG titers induced in mice by free N, **22** and **25** (with 10 µg N) were 60 846, 311 103, and 137 864, respectively; and the mean IgG titers induced by free N, **22** and **25** (with 1 µg N) in mice were 26 366, 106 321, and 60 824, respectively (Figure 10b). These results showed that **22** induced higher levels of anti‐N IgG antibodies than **25**, highlighting the advantage of ^TCC^Sia‐Ace‐Dex‐Rd in enhancing IgG responses. Furthermore, the mean titer of IgG antibodies induced in mice 35 days after three immunizations with N + **24** was 483 377, eightfold higher than that induced by free N, highlighting that **24** is a potent agonist to activate APCs, including macrophages, which can enhance N‐specific antibody production. NP‐based adjuvants have been reported to enhance humoral immunity to proteins.^[^ ^33^ ^]^ However, there is limited research on actively targeting NP adjuvants to enhance the humoral immunity of proteins. NP **22** induced higher titers of IgG than N + **24**, highlighting that covalently linking N to **24** could further enhance the N‐specific humoral immune response.

Finally, we evaluated the humoral immunity elicited by **22** and **25** in rabbits. Rabbits were immunized with sterile solutions of free N, **22**, and **25** on days 0, 14, and 28, respectively, and sera were collected on day 35 for ELISA testing (Figure 10c). On day 35, free N, **22** and **25** induced mean IgG titers in rabbits of 54 884, 222 951, and 82 633, respectively (Figure 10d). This suggested that **22** induced higher levels of anti‐N IgG in rabbits than **25**. Taken together, **22** can generate strong CTL and humoral responses in both mice and rabbits.

## 3 Conclusion

Due to its biocompatibility and biodegradability, ethoxy acetalated dextran (Ace‐Dex) glyconanoparticle is an attractive vehicle for vaccine development. However, insufficient CTL activation and IgG production observed upon administration of Ace‐Dex NPs are some of the significant obstacles to overcome for successful vaccines. To enhance CTL and IgG responses, we explored high‐affinity glycan ligand‐modified Ace‐Dex NPs to target macrophages as a versatile platform for developing the next generation of protein‐based vaccines.

PA and Rd were bound to partially oxidized azide‐containing Ace‐Dex (Oxi‐Ace‐Dex‐Az) NPs via imine bond formation. The resulting NPs were then combined with 9‐*N*‐(4H‐thieno[3,2‐*c*]chromene‐2‐carbamoyl)‐Sia*α*2−3Gal*β*1−4GlcNAc*β*Pro‐DBCO (^TCC^Sia‐LacNAc‐DBCO) by a strain‐promoted azide‐alkyne cycloaddition reaction yielding ^TCC^Sia‐Ace‐Dex‐PA‐Rd NPs; these NPs are suitable for delivering PAs, including those from cancer and viruses, to macrophages in a targeted manner.

When applying the ^TCC^Sia‐Ace‐Dex‐PA‐Rd platform to develop anticancer vaccines, OVA, Rd and ^TCC^Sia‐LacNAc‐DBCO were conjugated with Oxi‐Ace‐Dex‐Az NPs to obtain ^TCC^Sia‐Ace‐Dex‐OVA‐Rd, which resulted in a potent and lasting OVA‐specific CTL response and high titers of IgG, producing superior antitumor immunotherapy. This is the first time ^TCC^Sia‐LacNAc‐NPs have shown antitumor efficacy in vivo.

To develop an unprecedented SARS‐CoV‐2 vaccine based on the ^TCC^Sia‐Ace‐Dex‐PA‐Rd platform, the combination of Oxi‐Ace‐Dex‐Az NPs with SARS‐CoV‐2 RBD, Rd, and ^TCC^Sia‐LacNAc‐DBCO provides the ^TCC^Sia‐Ace‐Dex‐RBD‐Rd vaccine, this vaccine produced highly potent RBD‐neutralizing IgG against live SARS‐CoV‐2 virus. Head‐to‐head immunization studies of ^TCC^Sia‐Ace‐Dex‐RBD‐Rd and a reported SARS‐CoV‐2 vaccine (RBD+Alum+CpG) showed that ^TCC^Sia‐Ace‐Dex‐RBD‐Rd elicited significantly stronger anti‐RBD IgG responses compared to RBD+Alum+CpG, demonstrating the superiority of ^TCC^Sia‐Ace‐Dex‐PA‐Rd platform for vaccine development.

To further explore a universal SARS‐CoV‐2 vaccine against SARS‐CoV‐2 and its VOCs, we also prepared the ^TCC^Sia‐Ace‐Dex‐N‐Rd vaccine containing highly conserved SARS‐CoV‐2 N protein; this vaccine generated robust anti‐N CTL responses and high titers of anti‐N IgG, highlighting its great potential against SARS‐CoV‐2 and its VOCs, including Omicron (BA.1 to BA.5). An important direction in the future is to combine N protein, RBD, and Rd with ^TCC^Sia‐Ace‐Dex NPs in one particle to design ^TCC^Sia‐Ace‐Dex‐N‐RBD‐Rd as a next‐generation universal SARS‐CoV‐2 vaccine. Overall, this study can open up a new direction for developing anticancer and anti‐SARS‐CoV‐2 vaccines using glyconanoparticles grafted with glycan ligands.

## Conflict of Interest

The authors declare no conflict of interest.

## Author Contributions

Y.G. and W.W. contributed equally to this work. X.W. conceived the concept of developing next‐generation protein vaccines and supervised the project. X.W., Y.G., T.J., and S.C. designed the experiments. Y.G., W.W., Y.Y., Q.Z., C.Y., X.J., Y.L., M.Z., C.Y., and W.Z. performed the experiments. X.W. and Y.G. wrote the manuscript. All authors revised the manuscript.

## Supporting information

Supporting Information

## Data Availability

The data that support the findings of this study are available from the corresponding author upon reasonable request.
