# Supplementary material for: Developing Next‐Generation Protein‐Based Vaccines Using High‐Affinity Glycan Ligand‐Decorated Glyconanoparticles
Source: Adv Sci (Weinh). 2022 Nov 18;10(2):2204598. doi: 10.1002/advs.202204598 (PMC9839878; doi:10.1002/advs.202204598)
Supplement: Supplementary file 1 — Supporting Information [file ADVS-10-2204598-s001.pdf]

ADVANCED SCIENCE
Open Access

Supporting Information

for *Adv. Sci.*, DOI 10.1002/advs.202204598

Developing Next-Generation Protein-Based Vaccines Using High-Affinity Glycan Ligand-Decorated Glyconanoparticles

Yanan Gao, Wei Wang, Yunru Yang, Qingyu Zhao, Chendong Yang, Xiaoying Jia, Yang Liu, Minmin Zhou, Weihong Zeng, Xuefei Huang, Sandra Chiu, Tengchuan Jin* and Xuanjun Wu**

Supporting Information

Developing next-generation protein-based vaccines using high-affinity glycan ligand-decorated glyconanoparticles

Yanan Gao, Wei Wang, Yunru Yang, Qingyu Zhao, Chendong Yang, Xiaoying Jia, Yang Liu, Minmin Zhou, Weihong Zeng, Xuefei Huang, Sandra Chiu, Tengchuan Jin*, and Xuanjun Wu**

Table of Contents

Figure S1. FTIR spectrum of Oxi-Ace-Dex-Az NPs (**1**). S4

Figure S2. *In vivo* toxicity of ^{TCC}Sia-Ace-Dex-OVA-Rd (**17**) by histological analysis. S4

Figure S3. *In vivo* toxicity of ^{TCC}Sia-Ace-Dex-RBD-Rd (**21**) by histological analysis. S5

Figure S4. IgG subtype titers in mice immunized with ^{TCC}Sia-Ace-Dex-RBD-Rd (**21**). S5

Figure S5. Vaccination with ^{TCC}Sia-Ace-Dex-N-Rd (**22**) activates immune cells. S6

Figure S6. Detection of sera TNF-α and IFN-γ. S7

Figure S7. Uptake efficiency of ^{TCC}Sia-Ace-Dex-N^{FITC}-Rd by CD169^{+} BMMs. S7

Materials **S8**

General methods for the synthesis **S9**

Synthesis of 4H-thieno[3,2-c] chromene-2-carboxylic acid (SI-2) **S9**

Synthesis of TCC-NHS 7 **S9**

Synthesis of 3′-Azidopropyl 2-Deoxy-2-acetamido-*β*-D-glucopyranoside (3) **S10**

Enzymatic synthesis of Gal*β*1−4GlcNAc*β*ProN$_3$ (4) **S12**

Synthesis of 9NH$_2$-Sia*α*2−3Gal*β*1−4GlcNAc*β*ProN$_3$ (6) **S13**

Synthesis of 9-*N*-(4H-thieno[3,2-*c*]chromene-2-carbamoyl)-Sia*α*2−3Gal*β*1−4GlcNAc-*β* ProN$_3$ (8) **S13**

Synthesis of 9-*N*-(4H-thieno[3,2-*c*]chromene-2-carbamoyl)-Sia*α*2−3Gal*β*1−4GlcNAc-*β*ProNH$_2$ (9) **S14**

Synthesis of 2 **S15**

Synthesis of CDI-TEG-azide **S15**

Synthesis of dextran-azide (Dex-Az) **S16**

Synthesis of partially oxidized dextran-azide (Oxi-Dex-Az) **S16**

Synthesis of Oxi-Ace-Dex-Az polymer **S16**

Synthesis of Oxi-Ace-Dex-Az NPs (1) **S16**

Synthesis of OVA^{FITC} **S17**

Synthesis of ^{TCC}Sia-Ace-Dex-OVA^{FITC}-Rd NPs **S17**

Synthesis of ^{TCC}Sia-Ace-Dex-OVA-Rd NPs **S18**

Quantification of OVA, Rd and ^{TCC}Sia-LacNAc in the NPs **S18**

Table S1. The amounts of OVA/OVA^{FITC}, ^{TCC}Sia-LacNAc, and Rd in the NPs. **S18**

The release profiles of OVA and Rd from the NPs **S18**

Preparation of CD169^{+} BMMs **S19**

Detection of OVA$_{257–264}$ presented by MHC-I of BMMs (CD169^{+}) **S19**

B3Z T cell activation study **S19**

Immunization **S19**

***In vivo* CTL activity studies of OVA based NPs** **S20**

Evaluation of antibody responses elicited by OVA based NPs **S20**

EG7-OVA tumor challenge study **S21**

Synthesis of ^{TCC}Sia-Ace-Dex-RBD-Rd (21) **S21**

Table S3. The amounts of RBD, ^{TCC}Sia-LacNAc and Rd in the NPs **21**. **S21**

Antibody responses by ^{TCC}Sia-Ace-Dex-RBD-Rd NPs **S21**

Competitive ELISA **S22**

Authentic SARS-CoV-2 virus neutralization test in Vero E6 cells **S22**

Synthesis of ^{TCC}Sia-Ace-Dex-N-Rd (22) and ^{TCC}Sia-Ace-Dex-N$_{219–227}$-Rd (23) **S22**

Table S3. The amounts of N protein, ^{TCC}Sia-LacNAc and Rd in the NPs. **S23**

Quantification of N$_{219–227}$-MHC-I^{+}CD8^{+} cells **S23**

***In vivo* CTL activity studies of N based NPs** **S23**

Evaluation of antibody responses elicited by N based NPs **S24**

Product Characterization Spectra **S25**

References **S33**

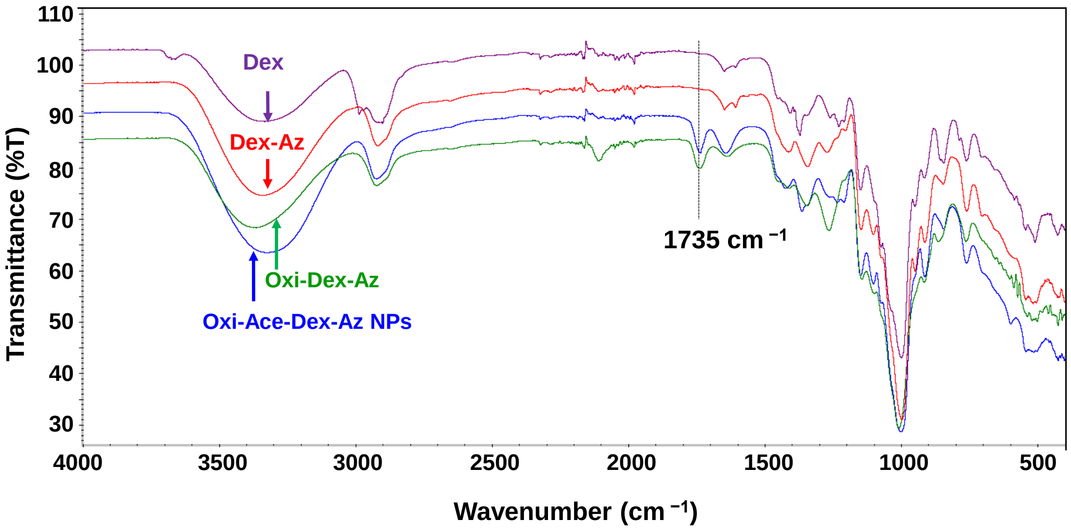

Figure S1. FTIR spectrum of Oxi-Ace-Dex-Az NPs (**1**). FTIR spectroscopy analysis revealed a signal at 1735 cm^{-1} in the Oxi-Dex-Az polymer and the Oxi-Ace-Dex-Az NPs (**1**) that was absent in the Dex and Dex-Az polymers without oxidation, indicating the presence of aldehyde groups on **1**.

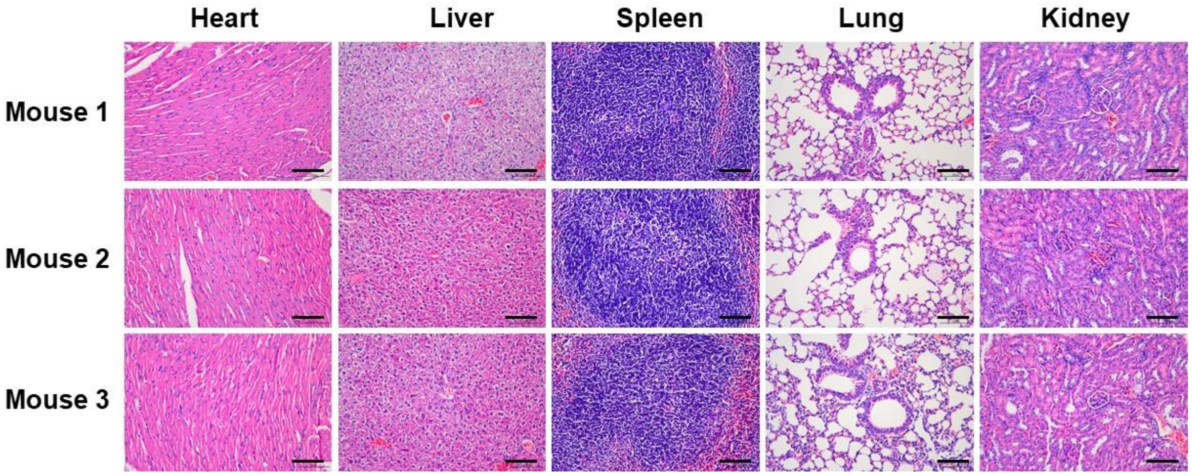

Figure S2. *In vivo* toxicity of ^{TCC}Sia-Ace-Dex-OVA-Rd (**17**) by histological analysis. C57BL/6 female mice were injected with EG7-OVA cells (1×10^6) on day 0. And a total of three subcutaneous immunizations with **17** were performed on days −7, 1, and 7. Then, mice were sacrificed by anesthesia on day 11. Organs from three mice immunized with **17** were harvested, sliced, and stained with hematoxylin/eosin (H&E). The results showed that no lesions were observed from these slides. Scale bars: 100 μm.

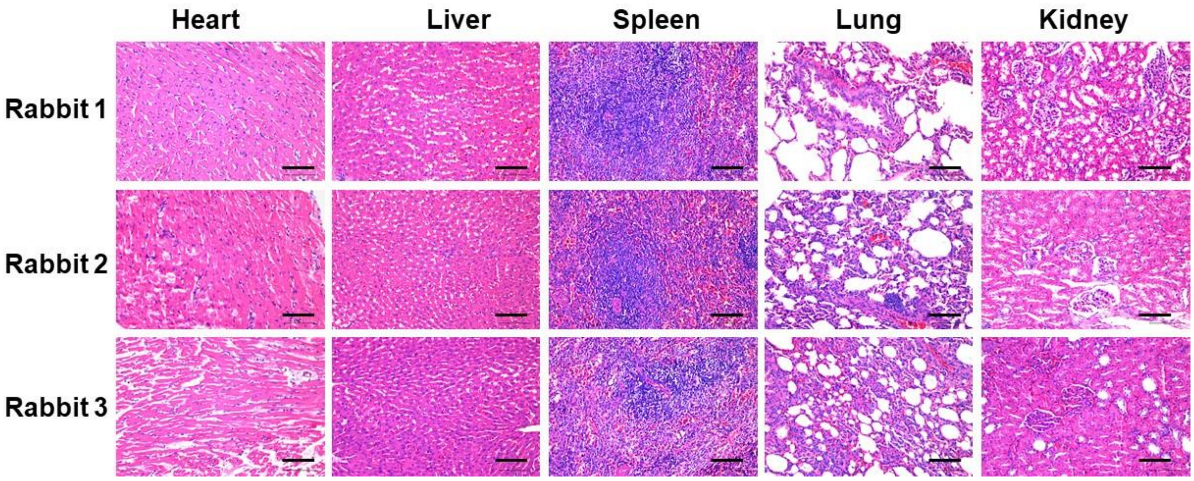

Figure S3. *In vivo* toxicity of ^{TCC}Sia-Ace-Dex-RBD-Rd (**21**) by histological analysis. Rabbits were subcutaneously immunized on days 0, 14, 28 with **21**. On day 35, organs from three rabbits immunized with **21** were harvested, sliced, and stained with hematoxylin/eosin. The results showed that no lesions were observed from these slides. Scale bars: 100 μm.

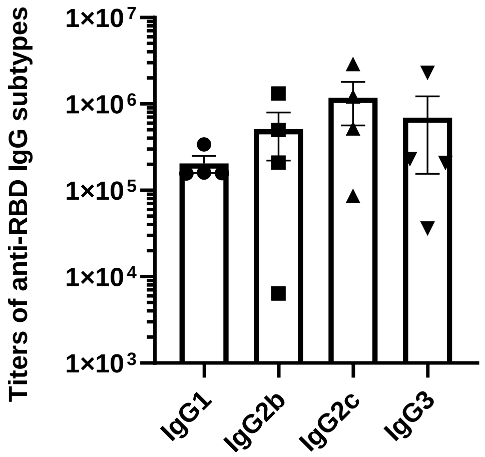

Figure S4. IgG subtype titers in mice immunized with ^{TCC}Sia-Ace-Dex-RBD-Rd (**21**). Each symbol represents one mouse. Mice were subcutaneously vaccinated with **21** (with 25 μg RBD in the NPs) on days 0, 14, 28, respectively. On day 35, sera were collected for ELISA analysis of IgG subtype titers. The results showed that vaccination with **21** led to robust Th1 and Th2 humoral responses.

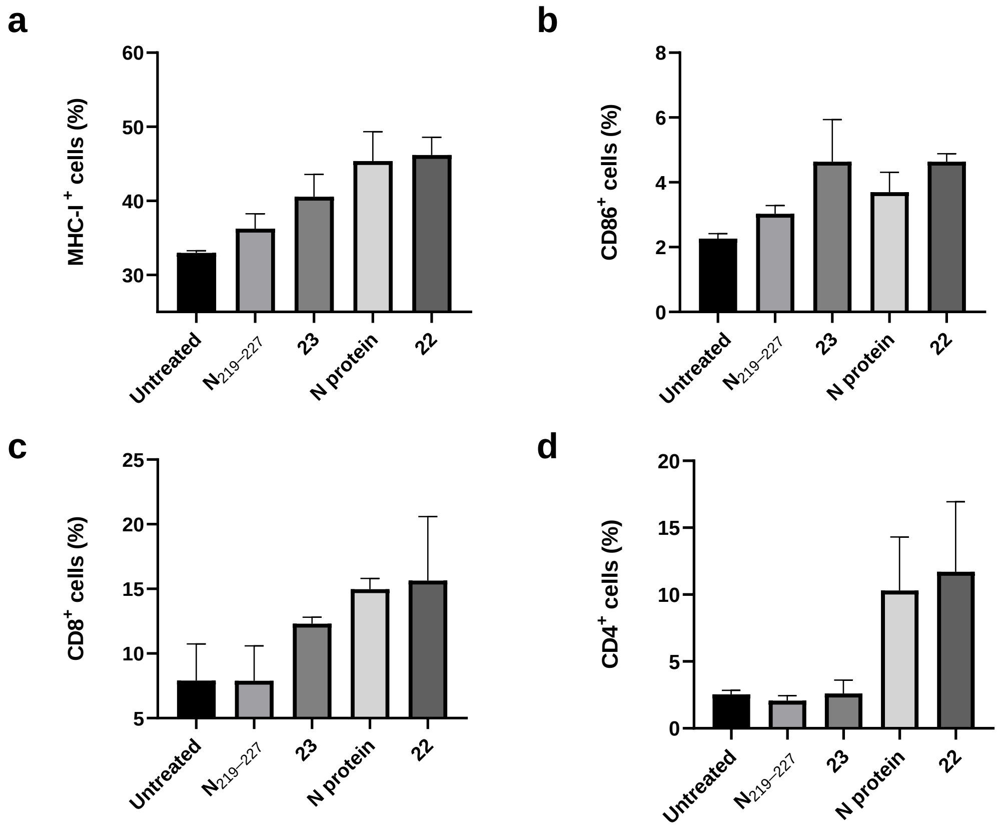

Figure S5. Vaccination with ^{TCC}Sia-Ace-Dex-N-Rd (**22**) activates immune cells. C57BL/6 mice were subcutaneously immunized on days 0 and 7 with free N (50 μg), $N_{219–227}$ (equivalent to the amount of $N_{219–227}$ in 50 μg N), **22** (with 50 μg N), or **23** (with the same amount of $N_{219–227}$ as the $N_{219–227}$ treated group). 14-day later, their spleens were collected for antibody staining. (a) The percentage of MHC-I$^+$ cells in splenocytes was determined by FITC-conjugated anti-mouse H-2K^b antibody staining. (b) The percentage of CD86$^+$ cells in splenocytes was determined by FITC-conjugated anti-mouse CD86$^+$ antibody staining. (c) The percentage of CD8$^+$ cells in splenocytes was determined by FITC-conjugated anti-mouse CD8 antibody staining. (d) The percentage of CD4$^+$ cells in splenocytes was determined by FITC-conjugated anti-mouse CD4$^+$ antibody staining. (n = 3 mice for each group). The results showed that vaccination of mice with NPs **23** and **22** upregulated MHC-I, CD86, and CD8 on splenocytes, indicating their high activation of T cells. The expression of CD8, MHC-I, CD86, CD8, and CD4 in mice immunized with **22** was similar to those vaccinated with N protein, indicating that binding with NP would not change the ability of N protein to activate immune cells. Despite similar increases in CD8, MHC-I, CD86, CD8, and CD4, **22** induced higher levels of N-specific CTLs *in vivo* (see **Figure 9**), highlighting the importance of incorporating N protein into NP.

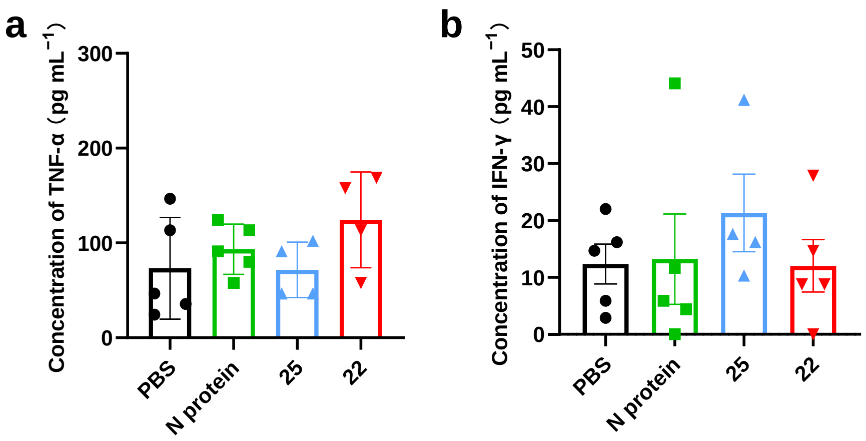

Figure S6. Detection of sera TNF-α and IFN-γ. C57BL/6 female mice were immunized subcutaneously on days 0, 14, 28 with PBS, N protein, **25**, and **22**, respectively. On day 35, sera were collected and analyzed with TNF-α and IFN-γ ELISA kits. Each symbol represents one mouse. The results showed that TNF-α and IFN-γ were not increased in **22** immunized mice, suggesting that vaccination with **22** can induce a strong anti-SARS-CoV-2 CTL immune response without generating a deleterious cytokine storm.

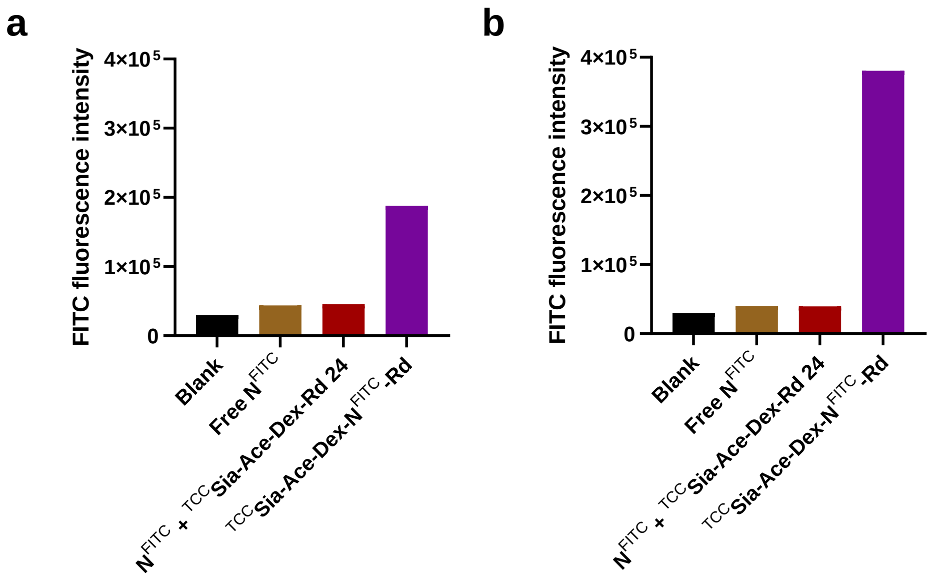

Figure S7. Uptake efficiency of ^{TCC}Sia-Ace-Dex-N^{FITC}-Rd by CD169^{+} BMMs. Detection of free N^{FITC}, ^{TCC}Sia-Ace-Dex-N^{FITC}-Rd, N^{FITC} + ^{TCC}Sia-Ace-Dex-Rd **24** within CD169^{+} BMMs upon incubation with the NPs (containing the same amount of N^{FITC}, 50 μg) for (a) 6 h or (b) 24 h by flow cytometry. It was found that ^{TCC}Sia-Ace-Dex-N^{FITC}-Rd was taken up by CD169^{+} BMMs more efficiently than N^{FITC} + **24**.

Materials

Ovalbumin (OVA) peptide $OVA_{257-264}$ (SIINFEKL) and SARS-CoV-2 nucleocapsid protein-derived peptide $N_{219-227}$ (LALLLLDRL) were acquired from NJPeptide (Nanjing, China). Chlorophenol red-β-D-galactopyranoside (CPRG) was purchased from J&K Scientific. LysoTracker Red was purchased from Beyotime. PE-conjugated anti-mouse H-$2K^b$ bound to SIINFEKL antibody (Clone: 25-D1.16) and FITC-conjugated anti-mouse CD8α antibody (Clone: 53-6.7) were purchased from Biolegend. FITC-conjugated anti-mouse IgG2a (κ isotype control), FITC-conjugated anti-mouse H-$2K^b$ (MHC Class I) antibody (Clone: AF6-88.5.5.3), FITC-conjugated anti-mouse CD4 antibody (Clone: RM4-5), and FITC-conjugated anti-mouse CD86 antibody (Clone: GL1) were purchased from Thermo Fisher Scientific. HRP-conjugated goat anti-mouse IgG, IgG1, IgG2b, IgG2c, and IgG3 were purchased from Abcam. Mouse TNF-α ELISA (Enzyme-Linked Immunosorbent Assay) Kit (SEKM-0034) and mouse IFN-γ ELISA Kit (SEKM-0031) were purchased from Solarbio. QuickSwitch™ Custom MHC Tetramer Kit from Mbl Beijing Biotech Co., Ltd. was used for the preparation of PE-conjugated $N_{219-227}$-MHC-I tetramer according to the manufacturer's instructions. Ethyl 4H-thieno[3,2-c]chromene-2-carboxylate and other chemicals were purchased from Sigma-Aldrich.

B3Z cells were cultured in RPMI 1640 medium with 10% fetal bovine serum (FBS), 100 IU mL^{-1} penicillin, 100 µg mL^{-1} streptomycin, 2 mM L-glutamine, 1 mM sodium pyruvate, 50 µM 2-mercaptoethanol. EG7-OVA cells from the American Type Culture Collection (ATCC) were cultured in RPMI 1640 medium supplemented with 10% FBS, 2 mM L-glutamine, 10 mM HEPES, 1.0 mM sodium pyruvate, 50 µM β-mercaptoethanol, 0.4 mg mL^{-1} G418, 100 IU mL^{-1} penicillin, and 100 µg mL^{-1} streptomycin. SARS-CoV-2 nucleocapsid protein and SARS-CoV-2 RBD protein (321−591 aa) were obtained from previous studies by Prof. Tengchuan Jin.[1] Vero E6 cells were maintained in Dulbecco's modified Eagle's medium (DMEM) supplemented with 10% FBS and 1% penicillin-streptomycin at 37 °C under a 5% CO_2 atmosphere. The SARS-CoV-2 WIV04 strain was initially isolated from a COVID-19 patient in 2019 (GISAID, accession no. EPI_ISL_402124)[2]. All processes in this study involving authentic SARS-CoV-2 were performed in a BSL-3 facility.

Confocal microscopic images were performed on Zeiss LSM880 using the following filters: λex@488 nm and λem@500−530 nm for OVA^{FITC}; λex@543 nm and λem@555−600 nm for LysoTracker Red. *In vivo* CTL studies were performed on a BD Accuri™ C6 Plus Flow Cytometer. Transmission electron microscopy (TEM) images were acquired from FEI Tecnai G2 F20 (Thermo Fisher Scientific). The synthetic compounds were characterized by a high-resolution mass spectrometer (LCMS-IT-TOF, LC-20A) and nuclear magnetic resonance (NMR) spectrometer (Agilent DD2 600). C57BL/6 female mice aged 6−10 weeks from Shandong University Laboratory Animal Center were used for all studies. All animal experiments were performed under the guidelines of the Animal Care and Use Committee of Shandong University (SYDWLL-2021-72 and SYDWLL-2022-045).

General methods for the synthesis

Synthesis of 4H-thieno[3,2-c] chromene-2-carboxylic acid (SI-2)

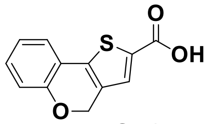

SI-2

To a solution of Ethyl 4H-thieno[3,2-c]chromene-2-carboxylate (**SI-1**, 105 mg, 0.40 mmol) in 5 mL of methanol was added an equal volume of 1N NaOH solution. The mixture was stirred at room temperature (RT) for 6 h, then washed 3 times with dichloromethane (DCM). Then, 1N hydrochloric acid (HCl) solution was added to the aqueous phase to precipitate the product. After filtration and washing with water, the white solid was dried to obtain **SI-2** (79 mg, 0.34 mmol, 85%).

Synthesis of TCC-NHS 7

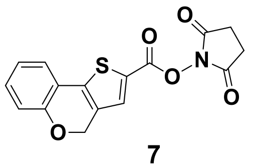

7

SI-2 (250 mg, 1.08 mmol) was dissolved in DCM (5 mL) with *N*-hydroxy succinimide (375 mg, 3.26 mmol), 1-Ethyl-3-(3-dimethylaminopropyl) carbodiimide (370 mg, 1.93 mmol) and triethylamine (100 µL). The reaction was complete after 1 h. The mixture was concentrated in vacuo, and the obtained residue was purified by silica gel column

chromatography (EtOAc/petroleum ether (PE), 1:1) to give TCC-NHS **7** (290 mg, 0.88 mmol, 81%) as a white solid.

Synthesis of 3′-Azidopropyl 2-Deoxy-2-acetamido-*β*-D-glucopyranoside (3)

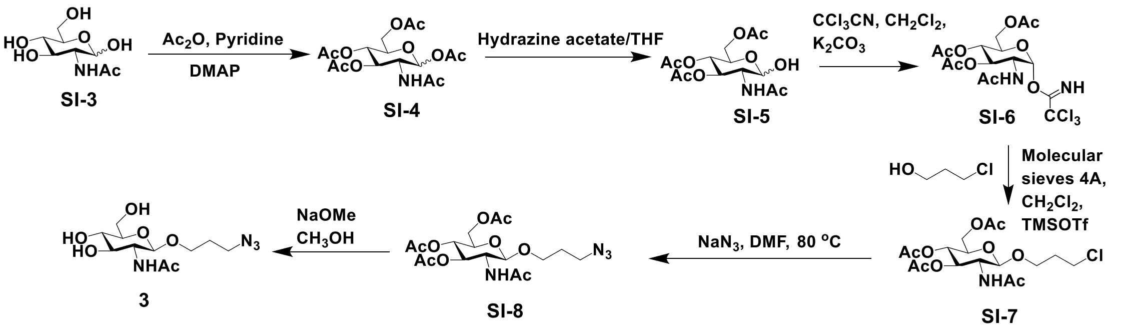

Scheme S1 Synthesis of GlcNAc*β*ProN$_3$ (3).

Synthesis of 2-Acetamido-1,3,4,6-tetra-*O*-acetyl-2-deoxy-D-glucopyranose (SI-4)

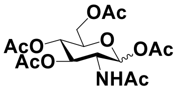

SI-4

2-Acetamido-2-deoxy-D-glucose (**SI-3**, 1.0 g, 4.5 mmol) was dissolved in anhydrous pyridine (12 mL). To the solution were added acetic anhydride (5.5 mL, 58.2 mmol) and 4-dimethylaminopyridine (DMAP, 0.11 g, 0.9 mmol). The mixture was stirred at RT overnight. The solution was evaporated to dryness and dissolved in DCM. The mixture was washed three times with water, and the organic phase was collected to give the crude product. The product was purified by silica gel column chromatography (EtOAc/PE, 1:1) to obtain the compound **SI-4** (1.6 g, 4.11 mmol, 91%) as a white solid.

Synthesis of 2-Acetamido-3,4,6-tri-*O*-acetyl-2-deoxy-D-glucopyranose (SI-5)

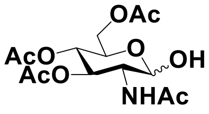

SI-5

To a solution of **SI-4** (1.0 g, 2.57 mmol) in THF (5 mL) was added hydrazine acetate (500 mg, 5.43 mmol). After stirring at RT for 30 min, the reaction mixture was concentrated

in vacuo. The crude product was purified by silica gel column chromatography (EtOAc/petroleum ether, 1:1) to give the compound **SI-5** (650 mg, 1.87 mmol, 73%) as a white solid.

Synthesis of 2-Acetamido-3,4,6-tri-*O*-acetyl-2-deoxy-α-D-glucopyranosyl Trichloro-acetimidate (SI-6)

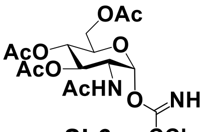

SI-6

To dry DCM (25 mL) with compound **SI-5** (1.0 g, 2.9 mmol) were added trichloroacetonitrile (1.1 mL, 11.0 mmol) and anhydrous potassium carbonate (2.0 g, 14.5 mmol). The reaction was carried out at RT for 3 h. The crude product was purified by silica gel column chromatography (EtOAc/PE, 1:1) to obtain **SI-6** (1.0 g, 2.0 mmol, 69%) as a white solid.

Synthesis of 3′-Chloropropyl 3,4,6-Tri-O-acetyl-2-deoxy-2-acetamido-*β*-D-glucopyranoside (SI-7)

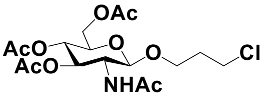

SI-7

3-Chloro-1-propanol (0.51 mL, 6.1 mmol) was added to a solution of **SI-6** (1.0 g, 2.0 mmol) in DCM (10 mL) with baked 4Å MS (0.5 g). After stirring at RT for 30 min under argon, TMSOTf (23 μL, 124.0 μmol) was slowly added dropwise at −20 °C. After the completion of the reaction, the crude product was purified by silica gel column chromatography (EtOAc/PE, 1:2 to 1:1) to obtain **SI-7** (480 mg, 1.13 mmol, 57%).

Synthesis of 3′-Azidopropyl 2-Acetamido-3,4,6-tri-O-acetyl-2-deoxy-*β*-D-glucopyranoside (SI-8)

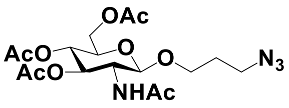

SI-8

To a solution of **SI-7** (500 mg, 1.18 mmol) in anhydrous DMF (10 mL) was added NaN_3 (767 mg, 11.8 mmol). After stirring overnight at 80°C, the reaction mixture was diluted with EtOAc (150 mL) and extracted with water. The organic phase was dried over Na_2SO_4. The crude product was purified by silica gel column chromatography (EtOAc/PE, 1:1) to give **SI-8** (440 mg, 1.02 mmol, 86%).

Synthesis of 3′-Azidopropyl 2-Deoxy-2-acetamido-*β*-D-glucopyranoside (3)

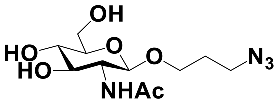

3

To a solution of **SI-8** (440 mg, 1.02 mmol) in anhydrous methanol (5 mL) was added a solution of sodium methoxide (30 wt.%) in methanol (100 μL). The reaction was carried out at RT for 3 h. Afterward, the mixture was neutralized with DOWEX HCR-W2 (H^+) resin, and the solvent was removed by rotary evaporation. The residue was purified by silica gel column chromatography (EtOAc/CH_3OH, 4:1) to give **3** (297 mg, 0.98 mmol, 96%). ^1H NMR (600 MHz, D_2O) δ 4.50 (d, J = 8.5 Hz, 1H), 3.96 (dt, J = 10.5, 5.6 Hz, 1H), 3.91 (dd, J = 12.4, 2.0 Hz, 1H), 3.73 (dd, J = 12.3, 5.6 Hz, 1H), 3.71 – 3.62 (m, 2H), 3.55 – 3.51 (m, 1H), 3.46 – 3.33 (m, 4H), 2.04 (s, 3H), 1.83 (p, J = 6.7 Hz, 2H). ^{13}C NMR (150 MHz, D_2O) δ 174.46, 101.09, 75.76, 73.66, 69.82, 66.99, 60.63, 55.48, 47.69, 28.01, 22.05. HRMS (ESI) m/z calcd for $C_{11}H_{20}N_4O_6$ $[M+H]^+$ 305.1456, found 305.1455.

Enzymatic synthesis of Gal*β*1−4GlcNAc*β*ProN$_3$ (4)

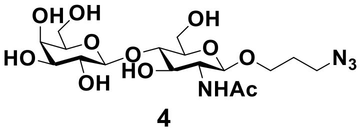

4

To Tris-HCl buffer (100 mM, pH 7.5, 10 mL) containing $MgCl_2$ (20 mM) was added **3** (59.4 mg, 0.2 mmol), UDP-galactose (UDP-Gal, 158.6 mg, 0.28 mmol). Then, *β*1,4-galactosyltransferase (Lgtb, 440 μg) was added to the reaction. The reaction was kept in a 37 °C incubator with shaking at 100 rpm overnight. The reaction was monitored by TLC (EtOAc/MeOH/H_2O/HOAc, 12:3:2:1). After the reaction was complete, an equal volume of

cold ethanol (EtOH) was added to stop the reaction. The residue was purified by BioGel P-2 gel column chromatography (eluted with H_2O), DEAE Sepharose fast flow (eluted with 0.02 M NaCl solution), and the second pass of BioGel P-2 gel to give the pure product **4** (83.0 mg, 0.18 mmol, 90%). ^{1}H NMR (600 MHz, D_2O) δ 4.55 (d, J = 7.6 Hz, 1H), 4.49 (d, J = 7.8 Hz, 1H), 4.03 – 3.96 (m, 2H), 3.94 (d, J = 3.4 Hz, 1H), 3.85 (dd, J = 12.3, 5.2 Hz, 1H), 3.81 – 3.64 (m, 10H), 3.61 (ddt, J = 7.5, 5.2, 2.3 Hz, 1H), 3.55 (dd, J = 10.0, 7.8 Hz, 1H), 3.39 (td, J = 6.5, 2.9 Hz, 2H), 2.06 (s, 3H), 1.89 – 1.82 (m, 2H). ^{13}C NMR (150 MHz, D_2O) δ 174.46, 102.80, 101.03, 78.35, 75.28, 74.66, 72.41, 72.30, 70.90, 68.49, 67.12, 60.97, 55.03, 47.72, 28.04, 22.14, 16.74. HRMS (ESI) m/z calcd for $C_{17}H_{30}N_4O_{11}$ $[M+H]^+$ 467.1984, found 467.2001.

Synthesis of 9NH$_2$-Sia*α*2−3Gal*β*1−4GlcNAc*β*ProN$_3$ (6)

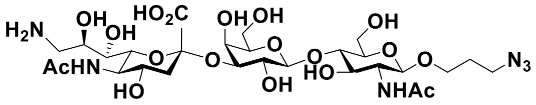

6

To Tris-HCl buffer (100 mM, pH 8.5, 10 mL) containing $MgCl_2$ (20 mM) was added **4** (46.6 mg, 0.1 mmol), 9NH$_2$-Sia **5**[3] (46.2 mg, 0.15 mmol) and cytidine-5'-triphosphate (CTP, 91.5 mg, 0.19 mmol). Then NmCSS (300 μg) and PmST1 (380 μg) were added to the reaction solution. The reaction mixture was kept in a 37 °C incubator with shaking at 100 rpm overnight. The reaction was monitored by TLC (EtOAc/MeOH/H_2O/HOAc, 9:3:2:1). When the reaction was complete, an equal volume of cold ethanol (EtOH) was added. After standing on ice for 0.5 h, the enzyme precipitate was removed by centrifugation (8000 rpm, 30 min). The supernatant was concentrated and subjected to BioGel P-2 gel column chromatography (eluted with H_2O), DEAE Sepharose fast flow (eluted with 0.05 M NaCl solution), and BioGel P-2 gel column chromatography to afford **6** (52.2 mg, 0.069 mmol, 69%) as a white solid. ^{1}H NMR (600 MHz, D_2O) δ 4.52 (d, J = 7.8 Hz, 1H), 4.50 (d, J = 7.9 Hz, 1H), 4.10 – 4.02 (m, 2H), 3.98 – 3.92 (m, 3H), 3.84 – 3.77 (m, 2H), 3.74 – 3.58 (m, 10H), 3.58 – 3.50 (m, 3H), 3.40 – 3.30 (m, 3H), 2.99 (dd, J = 13.1, 9.7 Hz, 1H), 2.72 (dd, J = 12.5, 4.6 Hz, 1H), 2.01 (s, 3H), 2.00 (s, 3H), 1.84 – 1.74 (m, 3H). ^{13}C NMR (150 MHz, D_2O) δ 174.95, 174.41, 173.72, 102.52, 101.04, 99.89, 78.36, 75.38, 74.97, 74.56, 72.42, 72.29, 69.86, 69.38, 68.11, 67.84, 67.27, 67.04, 60.88, 59.99, 54.93, 51.50, 47.64, 42.01, 39.47, 27.99, 22.04, 21.92. HRMS (ESI) m/z calcd for $C_{28}H_{48}N_6O_{18}$ $[M+H]^+$ 757.3098, found 757.3155.

Synthesis of

9-*N*-(4H-thieno[3,2-*c*]chromene-2-carbamoyl)-Sia*α*2−3Gal*β*1−4GlcNAc-*β* ProN$_3$ (8)

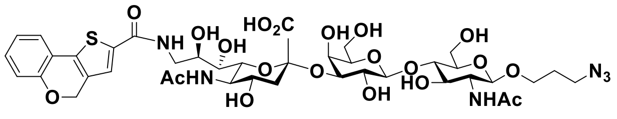

8

To a solution of **6** (100 mg, 0.13 mmol) in H_2O (2 mL) was added TCC-NHS **7** (64 mg, 0.20 mmol) in THF (2 mL). Triethylamine (100 μL) was added to the solution and stirred at RT for 1.5 h. The mixture was evaporated to dryness and purified by BioGel P-2 gel column chromatography to afford **8** (102 mg, 0.11 mmol, 85%) as a white solid. ^{1}H NMR (600 MHz, D_2O) δ 7.25 (dd, *J* = 7.6, 1.6 Hz, 1H), 7.24 (s, 1H), 7.20 (td, *J* = 7.8, 1.6 Hz, 1H), 6.95 (td, *J* = 7.5, 1.1 Hz, 1H), 6.86 (dd, *J* = 8.1, 1.1 Hz, 1H), 5.12 (s, 2H), 4.35 (d, *J* = 7.9 Hz, 1H), 4.28 (d, *J* = 8.1 Hz, 1H), 4.01 (dd, *J* = 9.8, 3.2 Hz, 1H), 3.95 (td, *J* = 8.8, 2.7 Hz, 1H), 3.88 (d, *J* = 3.2 Hz, 1H), 3.87 – 3.79 (m, 2H), 3.74 – 3.46 (m, 14H), 3.35 – 3.28 (m, 2H), 3.26 – 3.17 (m, 3H), 2.74 (dd, *J* = 12.4, 4.6 Hz, 1H), 2.00 (s, 3H), 1.97 (s, 3H), 1.75 (t, *J* = 12.2 Hz, 1H), 1.62 (pd, *J* = 7.0, 3.7 Hz, 2H). ^{13}C NMR (150 MHz, D_2O) δ 174.85, 174.23, 173.38, 163.47, 151.52, 136.62, 135.97, 131.98, 130.27, 126.21, 123.62, 122.58, 118.89, 116.39, 102.32, 100.90, 99.93, 77.78, 76.10, 75.20, 74.43, 72.83, 72.07, 70.79, 69.88, 69.04, 68.12, 67.28, 66.46, 65.61, 60.85, 59.63, 54.82, 51.59, 47.60, 42.89, 40.05, 27.98, 22.02, 21.96. HRMS (ESI) m/z calcd for $C_{40}H_{54}N_6O_{20}S$ $[M\text{-}H]^-$ 969.3041, found 969.2983.

Synthesis of

9-*N*-(4H-thieno[3,2-*c*]chromene-2-carbamoyl)-Sia*α*2−3Gal*β*1−4GlcNAc-*β*ProNH$_2$ (9)

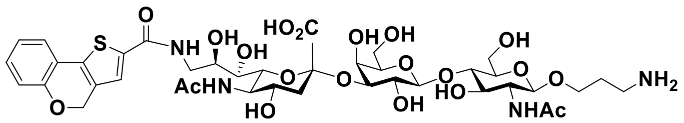

9

To **8** (100 mg, 0.1 mmol) in H_2O (2 mL) was added Pd/C (50 mg) and MeOH (2 mL). The reaction mixture was stirred under H_2 atmosphere until **8** was completely consumed. The reaction mixture was then filtered to remove Pd/C. The resulting **9** was used directly in the next step. ^{1}H NMR (600 MHz, D_2O) δ 7.30 (dd, *J* = 7.6, 1.6 Hz, 1H), 7.29 (s, 1H), 7.22 (td, *J* = 7.6, 1.6 Hz, 1H), 6.98 (td, *J* = 7.5, 1.1 Hz, 1H), 6.90 (dd, *J* = 8.2, 1.1 Hz, 1H), 5.15 (s, 2H), 4.35 (d, *J* = 7.8 Hz, 1H), 4.30 (d, *J* = 8.4 Hz, 1H), 4.02 (dd, *J* = 9.9, 3.2 Hz, 1H), 3.96 (td, *J* =

8.7, 2.8 Hz, 1H), 3.89 (d, *J* = 3.2 Hz, 1H), 3.87 – 3.81 (m, 2H), 3.72 – 3.46 (m, 16H), 3.42 (dt, *J* = 10.3, 5.7 Hz, 1H), 3.36 (ddd, *J* = 9.7, 4.5, 2.5 Hz, 1H), 3.27 (dd, *J* = 14.2, 8.5 Hz, 1H), 2.87 (t, *J* = 6.9 Hz, 2H), 2.74 (dd, *J* = 12.4, 4.6 Hz, 1H), 2.00 (s, 3H), 1.97 (s, 3H), 1.79 – 1.68 (m, 3H). ^{13}C NMR (150 MHz, D_2O) δ 174.89, 174.44, 173.45, 163.61, 151.60, 136.61, 136.00, 132.12, 130.30, 126.33, 123.67, 122.63, 119.01, 116.46, 102.37, 100.98, 99.91, 77.91, 75.99, 75.21, 74.46, 72.84, 71.96, 70.79, 69.83, 69.09, 68.11, 67.60, 67.25, 65.64, 62.80, 60.86, 54.78, 51.61, 42.81, 40.00, 37.58, 27.19, 21.99, 21.95. HRMS (ESI) m/z calcd for $C_{40}H_{56}N_4O_{20}S$ $[M-H]^-$ 943.3136, found 943.3086.

Synthesis of 2

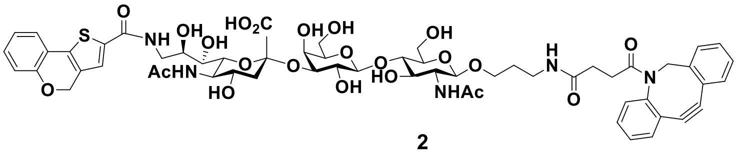

2

To **9** (94.4 mg, 0.1 mmol) in water (3 mL) was added DBCO-NHS ester (60 mg, 0.15 mmol) in THF (3 mL). To the mixture was added triethylamine (100 μl). After stirring at RT for 0.5 h, the residue after removal of the solvent was purified by BioGel P-2 gel column chromatography to give **2** (69 mg, 0.056 mmol, 56%) as a white solid. ^{1}H NMR (600 MHz, DMSO) δ 7.74 (q, *J* = 8.6 Hz, 1H), 7.69 (s, 1H), 7.62 (q, *J* = 4.8 Hz, 2H), 7.52 – 7.41 (m, 4H), 7.40 – 7.35 (m, 2H), 7.33 (t, *J* = 7.2 Hz, 1H), 7.29 (d, *J* = 7.4 Hz, 1H), 7.23 – 7.16 (m, 1H), 6.97 (dd, *J* = 9.6, 5.6 Hz, 1H), 6.93 (t, *J* = 7.3 Hz, 1H), 5.25 (d, *J* = 3.1 Hz, 2H), 5.03 (dd, *J* = 14.2, 7.6 Hz, 1H), 4.80 (d, *J* = 3.5 Hz, 1H), 4.58 (s, 1H), 4.23 (d, *J* = 7.8 Hz, 1H), 3.92 (dd, *J* = 9.9, 3.2 Hz, 1H), 3.76 (d, *J* = 11.7 Hz, 2H), 3.70 – 3.53 (m, 7H), 3.52 – 3.40 (m, 8H), 3.21 (s, 3H), 3.15 – 3.11 (m, 1H), 3.03 – 2.86 (m, 2H), 2.72 (dd, *J* = 12.0, 4.7 Hz, 1H), 2.62 – 2.53 (m, 1H), 2.27 – 2.13 (m, 2H), 2.05 – 1.92 (m, 2H), 1.88 (s, 3H), 1.80 – 1.75 (m, 3H). ^{13}C NMR (150 MHz, DMSO) δ 175.59, 172.64, 172.42, 171.14, 170.42, 168.94, 160.93, 152.13, 151.66, 148.69, 148.47, 139.39, 134.81, 132.51, 132.35, 129.84, 129.68, 128.85, 128.19, 127.99, 127.85, 127.73, 127.66, 126.67, 125.43, 125.15, 123.45, 122.57, 122.27, 121.31, 119.41, 116.65, 114.19, 108.44, 104.31, 100.07, 75.84, 75.06, 73.06, 70.79, 68.71, 66.82, 65.44, 63.42, 62.19, 60.48, 56.77, 54.92, 54.80, 54.60, 52.97, 40.06, 33.40, 31.62, 30.38,

29.72, 29.06, 23.04, 22.50. HRMS (ESI) m/z calcd for $C_{59}H_{69}N_5O_{22}S$ $[M-H]^-$ 1230.4082, found 1230.3949.

Synthesis of CDI-TEG-azide

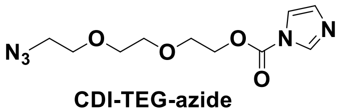

CDI-TEG-azide

To a flask was added azidotriethylene glycol (0.5 g, 2.85 mmol), molecular sieves (0.6 g, 4 Å), and THF (8 mL). Under argon, 1,1'-carbonyldiimidazole (CDI, 32 g, 3.2 mmol) was added to the reaction. After stirring for 30 min, the molecular sieves were removed by filtration. The solvent was removed by rotary evaporation. The residue was dissolved in EtOAc (50 mL), washed with ammonium chloride (10%, 50 mL) then brine (50 mL), and dried over anhydrous sodium sulfate. Rotary evaporation of the solvent gave CDI-TEG-azide (0.7 g, 2.6 mmol, 91%) as a clear oil. HRMS (ESI) m/z calcd for $C_{10}H_{15}N_5O_4$ $[M+H]^+$ 270.1197, found 270.1195.

Synthesis of dextran-azide (Dex-Az)

To DMSO (10 mL) containing dextran (Dex, 1 g, Mw: 9000−11000 g·mol^{-1}) was added CDI-TEG-Azide (0.7 g) and DMAP (1 g). Under argon, the mixture was stirred for 2 days. The reaction solution was then dialyzed, during which time the water was changed 4−5 times. The solution was lyophilized to obtain dextran-azide (Dex-Az). ^{1}H NMR (600 MHz, D_2O) δ 4.99 – 4.92 (m, 2H), 4.47 – 4.08 (m, 1H), 4.10 – 3.39 (m, 15H).

Synthesis of partially oxidized dextran-azide (Oxi-Dex-Az)

To dextran-azide (Dex-Az) (1 g) in water (10 mL) was added sodium periodate (0.22 g, 1.03 mmol). After stirring at RT for 5 h, the reaction mixture was dialyzed to remove small molecules. The product was lyophilized to give partially oxidized dextran-azide (Oxi-Dex-Az) as a white solid (0.7 g). Fourier transform infrared (FTIR) spectroscopy analysis shows a characteristic peak of the aldehyde group at 1735 cm^{-1} (see **Figure S1**). ^{1}H NMR (600 MHz, D_2O) δ 4.97 (d, J = 3.7 Hz, 1H), 4.25 – 3.18 (m, 9H), 2.71 (s, 1H).

Synthesis of Oxi-Ace-Dex-Az polymer

To the flask was added Oxi-Dex-Az polymer (1 g) and DMSO (10 mL). To the DMSO solution were added pyridinium *p*-toluenesulfonate (15.6 mg, 0.062 mmol) and

2-ethoxypropene (3.2 mL, 29.7 mmol) under nitrogen protection. After stirring at RT for 3 h, the reaction was quenched by adding triethylamine (1 mL). 100 mL of ultrapure water was then added. The mixture was centrifuged at 12,000 rpm for 20 min, and the pellet was washed with ultrapure water (50 mL × 2) and lyophilized to obtain the Oxi-Ace-Dex-Az polymer. ^{1}H NMR (600 MHz, Chloroform-*d*) δ 6.32 – 5.86 (m,1H), 5.27 – 4.55 (m, 4H), 4.16 – 2.86 (m, 22H), 2.37 – 2.07 (m, 14H), 2.07 – 0.95 (m, 25H).

Synthesis of Oxi-Ace-Dex-Az NPs (1)

20 mg of Oxi-Ace-Dex-Az polymer was dissolved in DCM (1 mL). The solution was sonicated on ice for 1 min (4 sec on, 2 sec off) using a sonicator (Xinzhi JY96-IIN, with a duty cycle of 50%) to obtain a primary emulsion, which was added to 2 mL of H_2O containing poly (vinyl alcohol) (PVA, Mw: 13−23 kg mol^{-1}, 3% w/w). After sonication on ice for 1 min, the emulsion was added to 0.3% (w/w) PVA in water (10 mL). After stirring for 3 h, the solvent was removed by centrifugation (12,000 rpm, 20 min). The particles were washed three times with ultrapure water (3 × 20 mL) and lyophilized to obtain Oxi-Ace-Dex-Az NPs, which were characterized by TEM and FTIR spectroscopy.

Synthesis of OVA$^{\mathbf{FITC}}$

To PBS (5 mL) containing 10 mg OVA was added FITC (0.6 mg) in DMSO (10 μL). After stirring at 4 °C for 2 h, the reaction mixture was subjected to ultrafiltration (MWCO 30 kDa) to remove unconjugated FITC. Lyophilization of samples trapped in ultrafiltration tubes to obtain OVA^{FITC}.

Synthesis of $^{\mathbf{TCC}}$Sia-Ace-Dex-OVA$^{\mathbf{FITC}}$-Rd NPs

To PBS (0.1M, pH 7.4, 200 μL) containing Oxi-Ace-Dex-Az NPs (10 mg) was added OVA^{FITC} (2 mg). The reaction was carried out at RT for 5 h. After that, resiquimod (Rd, 2 mg in 20 μL DMSO) and ^{TCC}Sia-LacNAc-DBCO **2** (2 mg) were added to the solution, followed by stirring at RT overnight. The pellet was obtained by centrifugation (12000 rpm, 20 min) and washed 3 times with ultrapure water (3 × 20 mL). The resulting particles were then subjected to ultrafiltration against ultrapure water, followed by lyophilization to obtain ^{TCC}Sia-Ace-Dex-OVA^{FITC}-Rd (**11**). For the synthesis of ^{TCC}Sia-Ace-Dex-OVA^{FITC} (**12**), the synthetic steps were the same as those of **11**, except that no Rd was added after the conjugation of Oxi-Ace-Dex-Az NPs with OVA^{FITC}. In addition, PEG-Ace-Dex-OVA^{FITC}-Rd

(**13**) and PEG-Ace-Dex-OVA^{FITC} (**14**) were synthesized with reference to the synthetic steps of **11** and **12**, respectively, except that the conjugated ligand **2** was replaced by DBCO-PEG$_3$-OH (CAS: 2566404-76-8, 2 mg). Ace-Dex OVA^{FITC}/Rd (**15**) and Ace-Dex OVA^{FITC} (**16**) were prepared by the double-emulsion evaporation technique as described elsewhere.[4]

Synthesis of ^{TCC}Sia-Ace-Dex-OVA-Rd NPs

To 200 μL of PBS (0.1 M, pH 7.4) containing 10 mg Oxi-Ace-Dex-Az NPs were added 2 mg of OVA and 2 mg of Rd. After shaking at RT for 5 h, ligand **2** (2 mg) was added and stirred at RT overnight. After centrifugation (12000 rpm, 20 min), the pellets were washed 3 times with ultrapure water (3 × 20 mL), followed by ultrafiltration (MWCO 30 kDa) to completely remove free OVA, Rd and ligand **2**. Lyophilization of the resulting particles yielded ^{TCC}Sia-Ace-Dex-OVA-Rd (**17**). ^{TCC}Sia-Ace-Dex-OVA (**18**), PEG-Ace-Dex-OVA-Rd (**19**), and PEG-Ace-Dex-OVA (**20**) were also prepared according to the synthetic steps of **11**−**14**, respectively, except that OVA was used instead of OVA^{FITC}.

Quantification of OVA, Rd and ^{TCC}Sia-LacNAc in the NPs

The amounts of OVA^{FITC} on NP$_S$ **11**−**16** were determined based on FITC absorbance measurements at 490 nm. The Bradford assay determined the amounts of OVA on NP$_S$ **17**−**20**. The amounts of ^{TCC}Sia-LacNAc in ^{TCC}Sia-Ace-Dex-OVA (**18**) and ^{TCC}Sia-Ace-Dex-OVA^{FITC} (**12**) were quantified based on TCC absorbance measurements at 345 nm. The amounts of Rd in PEG-Ace-Dex-OVA-Rd (**19**), PEG-Ace-Dex-OVA^{FITC}-Rd (**13**), and Ace-Dex OVA^{FITC}/Rd (**15**) were determined by UV absorption measurement at 321 nm. To determine the content of Rd and ^{TCC}Sia-LacNAc in ^{TCC}Sia-Ace-Dex-OVA^{FITC}-Rd (**11**) and ^{TCC}Sia-Ace-Dex-OVA-Rd (**17**), the NPs were degraded in water containing 0.5% TFA to release Rd. After ultrafiltration (MWCO 30 kDa), the absorbance at 345 nm of the intercepted precipitate was measured to quantify ^{TCC}Sia-LacNAc. In addition, the absorbance of the ultrafiltration filtrate at 321 nm was measured to quantify Rd.

Table S1. The amounts of OVA/OVA^{FITC}, ^{TCC}Sia-LacNAc, and Rd in the NPs.

NPs	OVA/OVA^{FITC} (μg) per mg NP	□^{TCC}Sia-LacNAc (μg) per mg NP	Rd (μg) per mg NP
^{TCC}Sia-Ace-Dex-OVA^{FITC}-Rd (**11**)	72	93	55
^{TCC}Sia-Ace-Dex-OVA^{FITC} (**12**)	69	79	

PEG-Ace-Dex-OVA^{FITC}-Rd (**13**)	84		64
PEG-Ace-Dex-OVA^{FITC} (**14**)	72		
Ace-Dex OVA^{FITC}/Rd (**15**)	71		61
Ace-Dex OVA^{FITC} (**16**)	70		
^{TCC}Sia-Ace-Dex-OVA-Rd (**17**)	76	67	62
^{TCC}Sia-Ace-Dex-OVA (**18**)	87	70	
PEG-Ace-Dex-OVA-Rd (**19**)	67		60
PEG-Ace-Dex-OVA (**20**)	79		

The release profiles of OVA and Rd from the NPs

To test the release profiles of OVA and Rd, 0.5 mg of ^{TCC}Sia-Ace-Dex-OVA-Rd (**17**) and PEG-Ace-Dex-OVA-Rd (**19**) were respectively dissolved in 1 mL of 0.1 M PBS (pH 7.4, 6.5, 6.0, 5.5 and 4.5) at 37 °C. At selected time intervals (6, 12, 24, 48 and 72 h), the supernatant was removed by centrifugation and the pellet was lyophilized. The amounts of unreleased OVA and Rd of the resulting particles were determined by the Bradford method and UV absorbance measurement, respectively.

Preparation of CD169^{+} BMMs

Bone marrow cells from C57BL/6 mice were isolated were cultured in RPMI 1640 medium supplemented with 10% heat-inactivated FCS, 2 mM glutamine, 100 IU mL^{-1} penicillin, 100 μg mL^{-1} streptomycin, 1 mM non-essential amino acids, 1 mM sodium pyruvate, 50 μM *β*-mercaptoethanol, 20 mM HEPES and 10 ng mL^{-1} macrophage-colony stimulating factor (M-CSF). After 7 days of culture, cells were stimulated with lipopolysaccharide (LPS) to obtain CD169-expressing mouse bone marrow-derived macrophages CD169 (CD169^{+} BMMs).

Detection of OVA$_{257-264}$ presented by MHC-I of BMMs (CD169^{+})

BMMs (5 × 10^{5} per well) were cultured overnight in 96-well plates. To the wells were added free OVA, ^{TCC}Sia-Ace-Dex-OVA-Rd (**17**), ^{TCC}Sia-Ace-Dex-OVA (**18**), PEG-Ace-Dex-OVA-Rd (**19**) and PEG-Ace-Dex-OVA (**20**), respectively, containing 0−1 μg OVA. After 24 h of incubation, the cells were washed 3 times with FACS buffer (containing 1% FBS and 0.1% NaN_3) and stained with PE-conjugated anti-mouse H-2K^{b}/SIINFEKL

antibody for 30 min on ice. Then, they were washed 3 times with FACS buffer and analyzed by flow cytometry.

B3Z T cell activation study

To BMMs (5×10^4 per well) were added free OVA, ^{TCC}Sia-Ace-Dex-OVA-Rd (**17**), ^{TCC}Sia-Ace-Dex-OVA (**18**), PEG-Ace-Dex-OVA-Rd (**19**) and PEG-Ace-Dex-OVA (**20**), respectively (containing 0−1 μg OVA). After 18 h of incubation, B3Z T cells (3×10^5) were added to each well and incubated for an additional 16 h. The medium was removed by centrifugation at 2500 rpm for 5 min. Then, 100 μL of CPRG solution (90 mg of $MgCl_2$, 9.1 mg of CPRG, and 0.125 mg of NP40 in 100 mL of PBS) was added to each well. After 6 h, the UV-Vis absorbance at 595 nm was recorded. The assay was performed in triplicate.

Immunization

The priority immunization procedure for *in vivo* CTL detection was to immunize mice for a total of three injections weekly (Days 0, 7 and 14). To test the effect of dose or immunization interval on CTL induction, other immune procedures (see **Figure 9g,h** in the main text) were also tested. The immune process for humoral evaluation was to immunize mice or rabbits at two weeks intervals for three injections (Days 0, 14 and 28).

***In vivo* CTL activity studies of OVA based NPs**

C57BL/6 mice were injected subcutaneously with free OVA, ^{TCC}Sia-Ace-Dex-OVA-Rd (**17**), ^{TCC}Sia-Ace-Dex-OVA (**18**), PEG-Ace-Dex-OVA-Rd (**19**) and PEG-Ace-Dex-OVA (**20**) *via* the neck on days 0, 7 and 14, respectively. All injected vaccines contained the same dose of OVA (100 μg). Spleens were isolated from naive C57BL/6 mice on day 21 to prepare suspensions. Half of the splenocytes (2×10^7, 2 mL) were labeled with a low concentration of carboxyfluorescein succinimidyl ester (CFSE concentration of 1 μM), and placed in a CO_2 incubator for 10 min. By removing free CFSE from the cell mixture by centrifugation at 1600 rpm for 3 min, $CFSE^{lo}OVA_{257–264}^{−}$ splenocytes were obtained. The other half of splenocytes (2×10^7, 2 mL) were labeled with a high concentration (10 μM) of CFSE and pulsed with $OVA_{257–264}$ (1 $\mu g \cdot mL^{-1}$) for 1 h to obtain $CFSE^{hi}OVA_{257–264}^{+}$ splenocytes. Afterward, $CFSE^{lo}OVA_{257–264}^{−}$ and $CFSE^{hi}OVA_{257–264}^{+}$ (1:1, 0.1 mL, 2×10^6) mixed cells were injected into untreated and vaccine-immunized mice *via* the tail vein. 24 h later, mouse splenocytes were prepared for FACS analysis. The changes in the ratio of $CFSE^{hi}$ to $CFSE^{lo}$ were

analyzed from the FITC-SSC plot to evaluate target cell viability. Furthermore, to assess whether ^{TCC}Sia-Ace-Dex-OVA-Rd (**17**) induces durable CTL responses, three immunizations were performed on days 0, 14, and 28, and *in vivo* CTL analysis was performed on day 100 (see **Figure 4e**).

Evaluation of antibody responses elicited by OVA based NPs

OVA (2 μg per well) in $NaHCO_3$/Na_2CO_3 buffer (0.05 M, pH 9.6 with 0.02% NaN_3) was added to 96-well microtiter plates (Thermo Scientific, Catalog: 442404) and coated overnight at 4 °C. After antigen coating, the plate was washed with PBST (containing 0.5% Tween-20). To each well was added 200 μL of 1% BSA in PBS. The plate was washed with PBST after blocking for 1 h. Diluted post-immunization sera were added to the wells, and the plates were incubated at 37 °C for 2 h, then washed with PBST. Next, a 1/2000 dilution of HRP-conjugated goat anti-mouse IgG, IgG1, IgG2b, IgG2c, and IgG3 in 0.1% BSA/PBS was added to each well. Plates were incubated at 37 °C for 1 h and washed with PBST. Then, 200 μL of 3,3',5,5'-tetramethylbenzidine (TMB) (for one plate: 2 mL DMSO, 5 mg TMB, 18 mL citrate buffer, and 20 μL H_2O_2) was added to each well. After 15 min of color development, 50 μL of 0.5 M H_2SO_4 was added to each well. Absorbance was measured at 450 nm. All samples were performed in triplicate. The cut off value for positivity was set at 2.1 × the OD value of the negative control.[5]

EG7-OVA tumor challenge study

On day −7, C57BL/6 mice were immunized with PBS, free OVA, ^{TCC}Sia-Ace-Dex-OVA-Rd (**17**), and PEG-Ace-Dex-OVA-Rd (**19**), respectively. All injected vaccines contained the same dose of OVA (100 μg). On day 0, EG7-OVA cells (1×10^6) were subcutaneously injected into the mice. Subsequently, two vaccine injections were given on the first and seventh days. Tumor volume was calculated with the formula: Volume (mm^3) = 1/2 (length × width × height).[6] Tumors were dissected on day 11, photographed, and weighed. The changes in the body weight of the mice were also monitored during this experiment.

Synthesis of ^{TCC}Sia-Ace-Dex-RBD-Rd (21)

To 200 μL of PBS (0.1 M, pH 7.4) containing 10 mg of Oxi-Ace-Dex-Az NPs (**1**) was added 2 mg of SARS-CoV-2 recombinant RBD protein (321−591aa) and 2 mg of Rd. After shaking at RT for 5 h, ligand **2** (2 mg) was added and stirred at RT overnight. After

centrifugation (12000 rpm, 20 min), the pellet was washed with water (3 × 20 mL), followed by ultrafiltration to remove unbound RBD, Rd, and ligand **2**. The resulting NPs were lyophilized to yield ^{TCC}Sia-Ace-Dex-RBD-Rd (**21**).

Table S2. The amounts of RBD, ^{TCC}Sia-LacNAc and Rd in the NP **21**.

NPs	RBD (μg) per mg NP	□^{TCC}Sia-LacNAc (μg) per mg NP	Rd (μg) per mg NP
^{TCC}Sia-Ace-Dex-RBD-Rd (**21**)	75	71	69

Antibody responses by ^{TCC}Sia-Ace-Dex-RBD-Rd NPs

For evaluation of ^{TCC}Sia-Ace-Dex-RBD-Rd (**21**) in mice, on days 0, 14, and 28, C57BL/6 mice were immunized with free RBD or ^{TCC}Sia-Ace-Dex-RBD-Rd (**21**) containing the same amount of RBD (10 μg). Sera were collected on days −1 and 35. SARS-CoV-2 RBD protein (1 μg RBD per well) was coated on the plate for ELISA. For evaluation of ^{TCC}Sia-Ace-Dex-RBD-Rd (**21**) in rabbits, on days 0, 14, and 28, rabbits (*n* = 3 per group) were immunized with free RBD or ^{TCC}Sia-Ace-Dex-RBD-Rd (**21**) containing the same amount of RBD (25 μg). Sera were collected on days −1 and 35. ELISA was performed using HRP-conjugated goat anti-rabbit IgG to determine rabbit serum titers. Furthermore, to determine antibody titers against Omicron RBD, SARS-CoV-2 B.1.1.529 (Omicron) Spike RBD Protein (His Tag) (1 μg per well) was used for plate coating.

Competitive ELISA

To test whether the antibodies produced by the NPs could effectively inhibit the interaction of RBD (or its mutant Omicron-RBD) and hACE2, we performed a competitive ELISA experiment. In brief, RBD protein or Omicron-RBD protein (0.3 μg per well) in $NaHCO_3/Na_2CO_3$ buffer (0.05 M, pH 9.6 with 0.02% NaN_3) was added to 96-well microtiter plates and coated overnight at 4 °C. The plate was washed with PBS containing 0.5% Tween-20 (PBST) and blocked with 5% defatted milk (w/v) in PBS for 2 h at rt. The plate was then washed 5 times with PBST and incubated with serially diluted serum (50 μL). After 30 min, 5 nM biotinylated hACE2-Fc (50 μL) was added, and the plate was incubated for an additional 30 min. After washing with PBST, the plate was incubated with HRP-labeled streptavidin (1:5000) for 30 min. Then, the plate was washed 5 times with PBST. To each

well was added 100 μL of TMB. After 10 min. 50 μL of H_2SO_4 (0.5 M) was added to each well to stop the reaction. Absorbance was measured at 450 nm.

$$\text{Inhibition} = \left(1 - \frac{\text{OD value of Sample}}{\text{OD value of positive control}}\right) \times 100\%.$$

IC_{50} was calculated by fitting the inhibition from serially diluted serum to a sigmoidal dose-response curve. The positivity cut-off value was set at the mean of negative control (pre-immune sera) + 2 × SD (standard deviation).[7]

Authentic SARS-CoV-2 virus neutralization test in Vero E6 cells

Authentic virus neutralization titers were determined as previously described.[8] Briefly, serum antibodies were serially diluted in DMEM containing FBS (2.5%) and mixed with an equal volume of virus suspension. After 1 h of incubation, the mixture was added to 24-well plates containing Vero E6 monolayers for incubation of an additional 1 h. The inoculate was replaced with DMEM containing FBS (2.5%) and carboxymethylcellulose (0.9%). Plates were fixed with paraformaldehyde (8%) and stained with 0.5% crystal violet after 3 days. Plaque reduction neutralizing titers were calculated using the "inhibitor versus normalized response (variable slope)" model in GraphPad (Prism 8.0) software. Cutoff values (geometric mean + 3 times geometric standard deviation) were calculated from negative controls.

Synthesis of ^{TCC}Sia-Ace-Dex-N-Rd (22) and ^{TCC}Sia-Ace-Dex-$N_{219–227}$-Rd (23)

To 200 μL of PBS (0.1 M, pH 7.4) containing 10 mg of Oxi-Ace-Dex-Az NPs (**1**) was added 2 mg of SARS-CoV-2 N protein (N) and 2 mg of Rd. After shaking at RT for 5 h, ligand **2** (2 mg) was added and stirred at RT overnight. After centrifugation (12000 rpm, 20 min), the pellet was washed with water (3 × 20 mL), followed by ultrafiltration to remove unbound N protein, Rd, and ligand **2**. The resulting NPs were lyophilized to yield ^{TCC}Sia-Ace-Dex-N-Rd (**22**). In addition, ^{TCC}Sia-Ace-Dex-$N_{219–227}$-Rd (**23**) was prepared similarly to **22**, except that $N_{219–227}$ was used instead of the N protein. As a control, PEG-Ace-Dex-N-Rd (**25**) without ^{TCC}Sia-LacNAc was prepared in the same manner as **22**, but DBCO-PEG_3-OH was used instead of ligand **2** to conjugate with Oxi-Ace-Dex-Az NPs.

Table S3. The amounts of N protein, ^{TCC}Sia-LacNAc and Rd in the NPs.

NPs	N or N^{FITC} (μg) per mg NP	□^{TCC}Sia-LacNAc (μg) per mg NP	Rd (μg) per mg NP
^{TCC}Sia-Ace-Dex-N-Rd (**22**)	89	67	62
^{TCC}Sia-Ace-Dex-Rd (**24**)		88	72

PEG-Ace-Dex-N-Rd (**25**)	79		70
PEG-Ace-Dex-N^{FITC}	76		
^{TCC}Sia-Ace-Dex-N^{FITC}	89	87	
^{TCC}Sia-Ace-Dex-N^{FITC}-Rd	72	83	69
PEG-Ace-Dex-N^{FITC}-Rd	69		78

Quantification of $N_{219–227}$-MHC-$I^+$$CD8^+$ cells

C57BL/6 female mice were subcutaneously immunized on days 0, 7 with free N, **22**, $N_{219–227}$, and **23**, respectively. On day 14, their spleens were collected. The percentage of $N_{219–227}$-MHC-$I^+$$CD8^+$ cells present in splenocytes was determined by staining splenocytes with PE-labeled LALLLLDRL-MHC-I tetramer prepared by QuickSwitchTM Custom MHC Tetramer Kit and FITC-labeled anti-mouse CD8 antibody.

In vivo CTL activity studies of N based NPs

C57BL/6 mice were injected subcutaneously with free N, **22**, $N_{219–227}$, and **23**, on days 0, 7, respectively. All injected vaccines contained the same dose of N protein (50 μg). The amount of $N_{219–227}$ and **23** injected was the same as that of $N_{219–227}$ on the N protein (50 μg). Spleens were isolated from naive C57BL/6 mice on day 14 to prepare suspensions. Half of the splenocytes (2×10^7, 2 mL) were labeled with 1 μM of CFSE and placed in a CO_2 incubator for 10 min. By removing free CFSE from the cell mixture by centrifugation at 1600 rpm for 3 min, $CFSE^{lo}N_{219–227}^{-}$ splenocytes were obtained. The other half of splenocytes (2×10^7, 2 mL) were labeled with 10 μM of CFSE, and then pulsed with $N_{219–227}$ with the sequence of LALLLLDRL (1 $\mu g \cdot mL^{-1}$) for 1 h to obtain $CFSE^{hi}N_{219–227}^{+}$ splenocytes. Afterward, $CFSE^{lo}N_{219–227}^{-}$ and $CFSE^{hi}N_{219–227}^{+}$ (1:1, 0.1 mL, 2×10^6) mixed cells were injected into untreated and vaccine-immunized mice *via* the tail vein. After 1 day, mouse spleens and lymph nodes were prepared as single-cell suspensions for FACS analysis.

Evaluation of antibody responses elicited by N based NPs

On days 0, 14, and 28, C57BL/6 mice were immunized with free N, ^{TCC}Sia-Ace-Dex-N-Rd (**22**), PEG-Ace-Dex-N-Rd (**25**), and ^{TCC}Sia-Ace-Dex-Rd (**24**) + N, respectively. Sera were collected on days −1 (pre-immunization) and 35 for subsequent experiments. The procedure for antibody titer determination for N-based NPs is similar to that of OVA NPs. Different steps involved: SARS-CoV-2 N protein (N) (1 μg N protein per

well) was coated on the plate. Second, the primary antibody-incubated serum was from mouse serum immunized with N-protein NPs. For rabbit immunization, on days 0, 14, and 28, rabbits (*n* = 3 per group) were vaccinated with free N, ^{TCC}Sia-Ace-Dex-N-Rd (**22**), PEG-Ace-Dex-N-Rd (**25**), respectively. Sera were collected on days −1, 21 and 35. To determine rabbit serum titers, ELISA was performed using HRP-conjugated goat anti-rabbit IgG.

Product Characterization Spectra

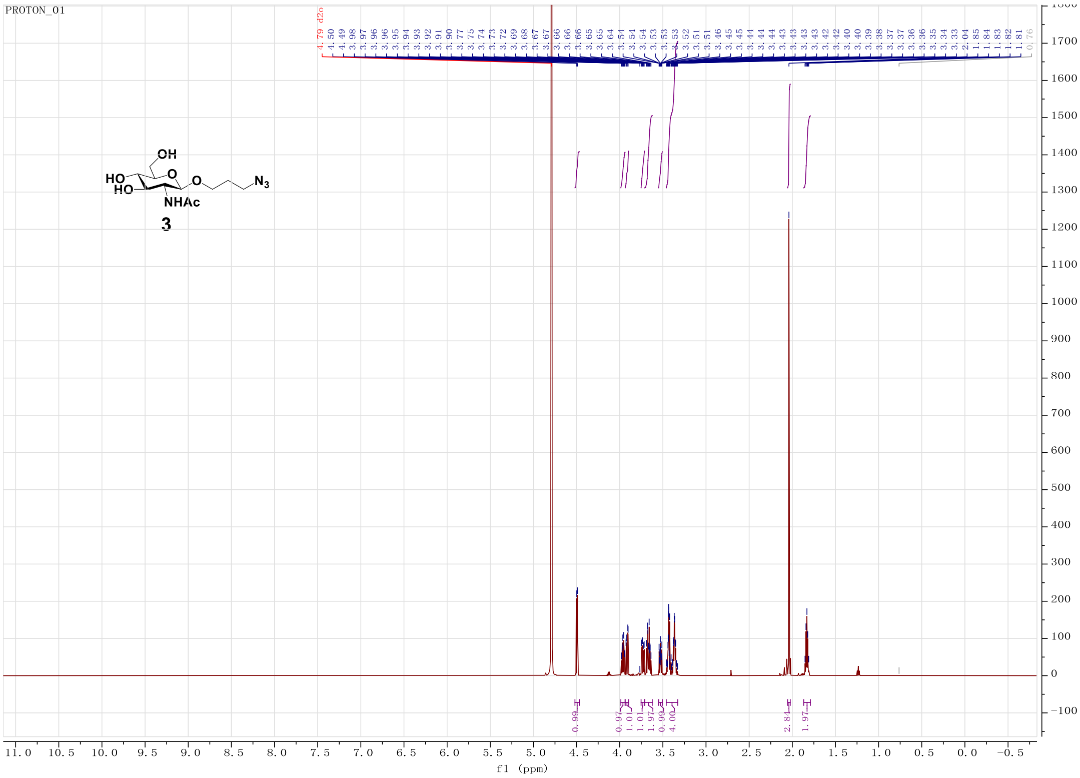

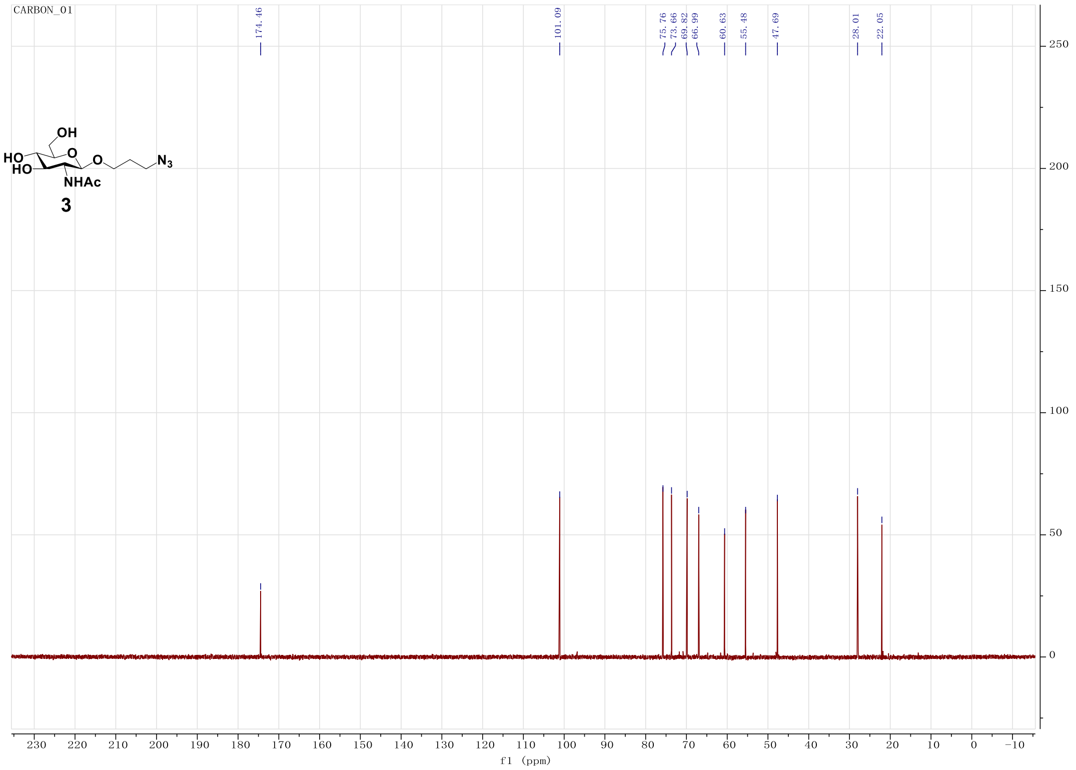

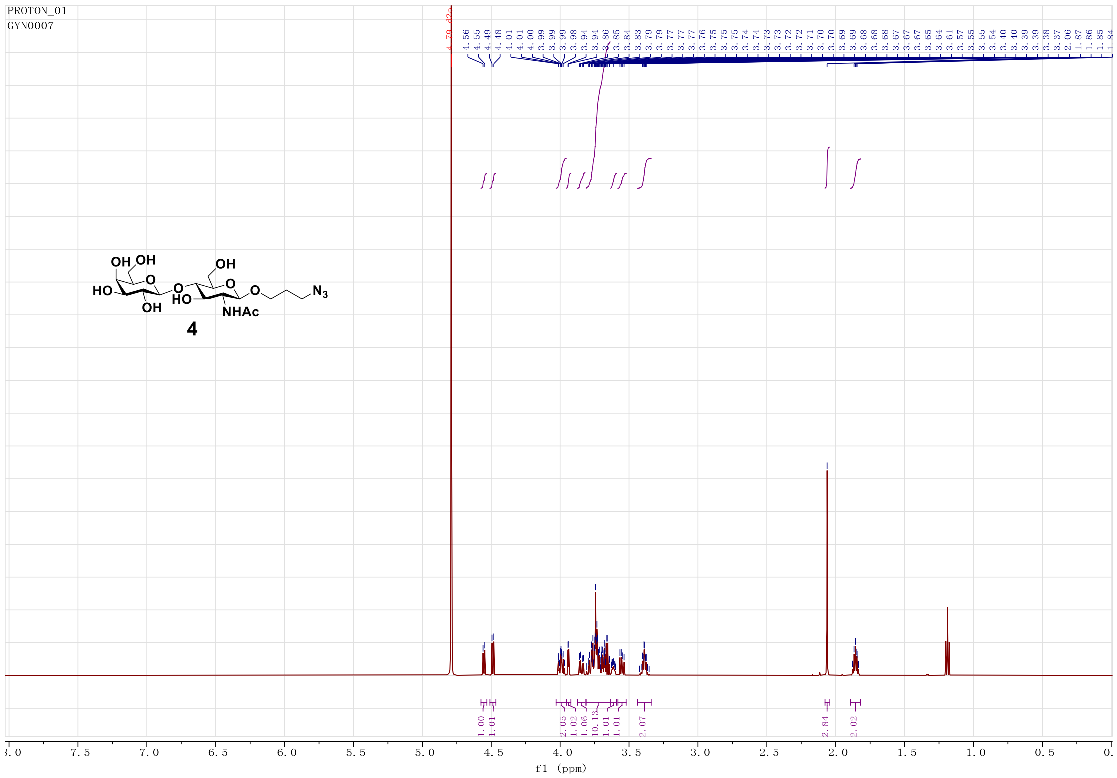
PROTON_01
GYN0007
4.56 4.55 4.49 4.48 4.01 4.01 4.00 3.99 3.99 3.99 3.98 3.94 3.94 3.86 3.85 3.84 3.83 3.79 3.79 3.77 3.77 3.76 3.75 3.75 3.75 3.74 3.74 3.73 3.73 3.72 3.72 3.71 3.70 3.70 3.69 3.69 3.68 3.68 3.68 3.67 3.67 3.65 3.64 3.61 3.57 3.55 3.55 3.54 3.40 3.40 3.39 3.39 3.38 3.37 2.06 1.87 1.86 1.85 1.84
OH OH
OH
HO
O
O
HO
O
O
N3
OH
NHAc
4
1.00 1.01 2.05 1.02 1.04 10.13 1.01 1.01 2.07 2.84 2.02
8.0 7.5 7.0 6.5 6.0 5.5 5.0 4.5 4.0 3.5 3.0 2.5 2.0 1.5 1.0 0.5 0.0
f1 (ppm)

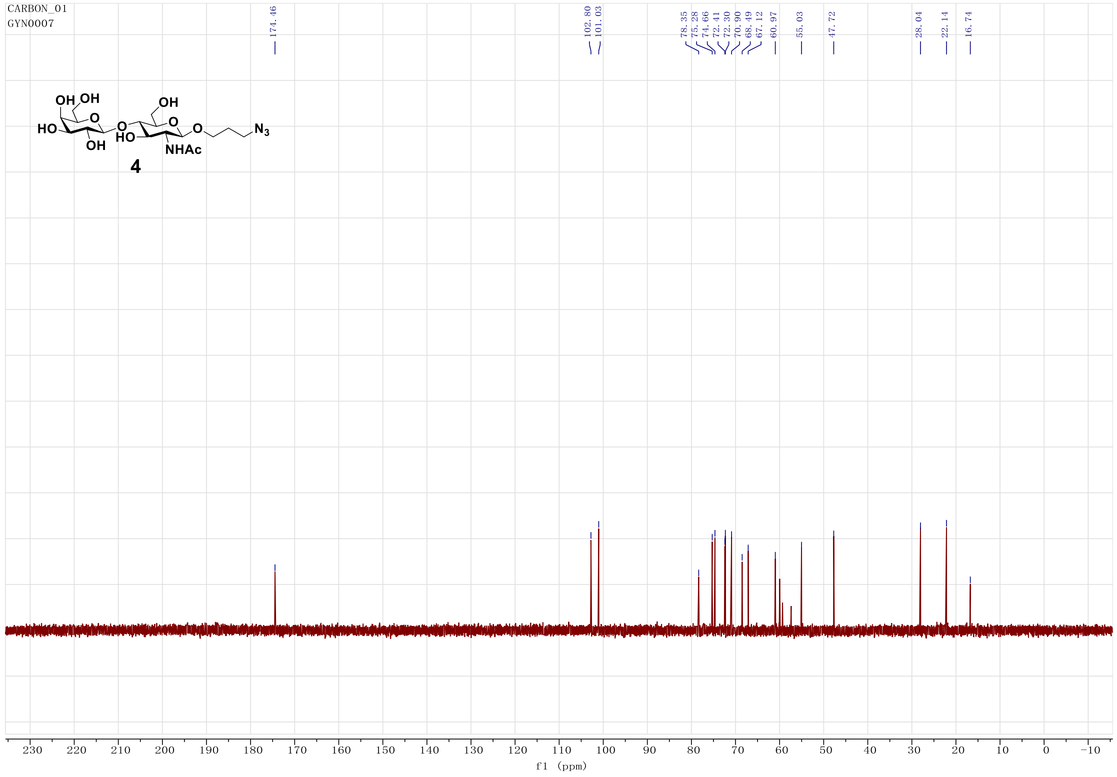
CARBON_01
GYN0007
174.46
102.80 101.03
78.35 75.28 74.66 72.41 72.30 70.90 68.49 67.12 60.97 55.03 47.72
28.04 22.14 16.74
OH OH
OH
HO
O
O
HO
O
O
N3
OH
NHAc
4
230 220 210 200 190 180 170 160 150 140 130 120 110 100 90 80 70 60 50 40 30 20 10 0 -10
f1 (ppm)

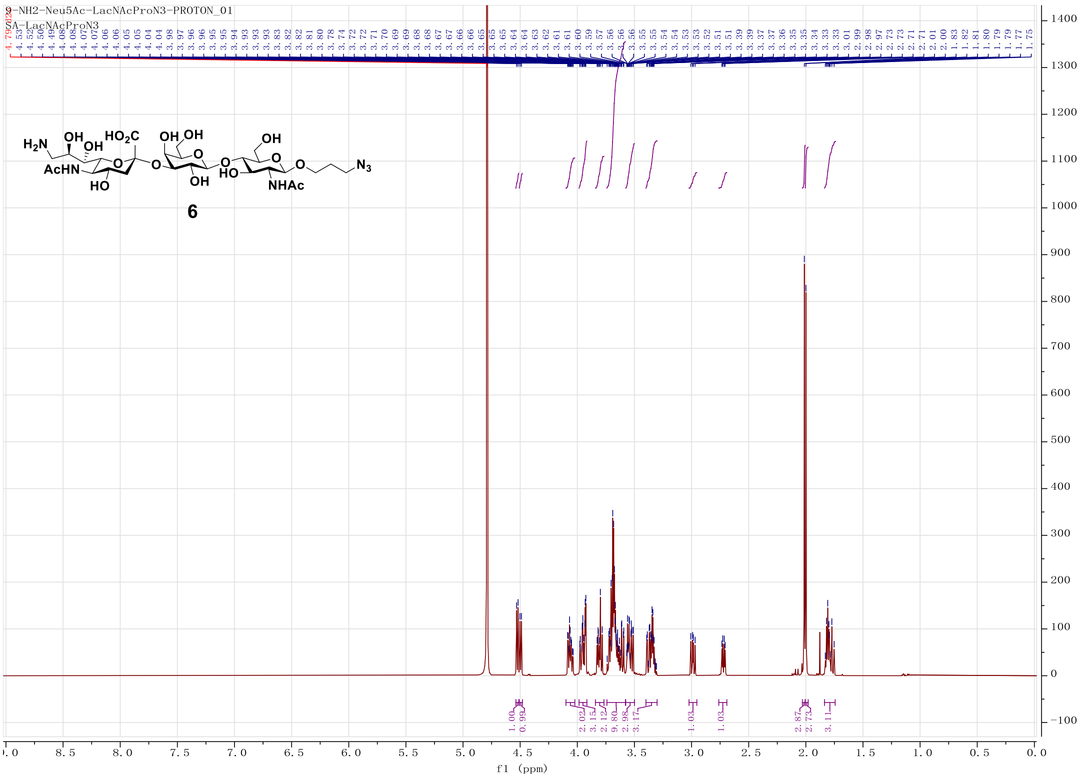
9-NH2-Neu5Ac-LacNAcProN3-PROTON_01
SA-LacNAcProN3
6
f1 (ppm)

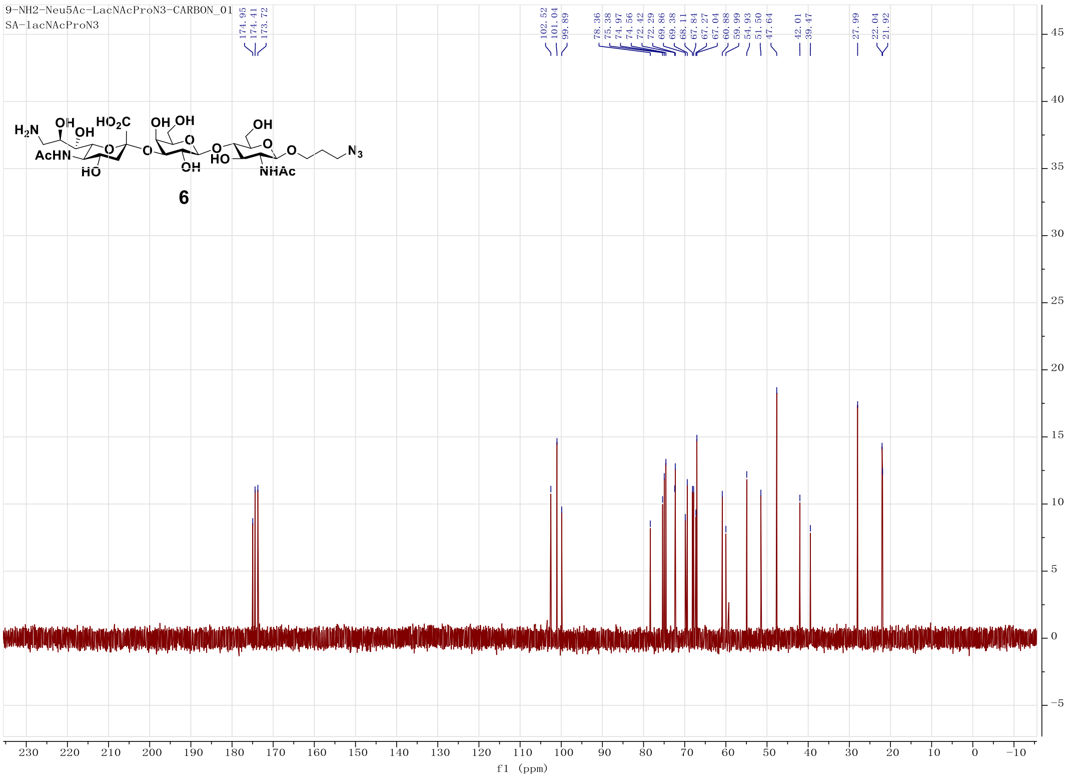
9-NH2-Neu5Ac-LacNAcProN3-CARBON_01
SA-lacNAcProN3
174.95
174.41
173.72
102.52
101.04
99.89
78.36
75.38
74.97
74.56
72.42
72.29
69.86
69.38
68.11
67.84
67.27
67.04
60.88
59.99
54.93
51.50
47.64
42.01
39.47
27.99
22.04
21.92
6
f1 (ppm)

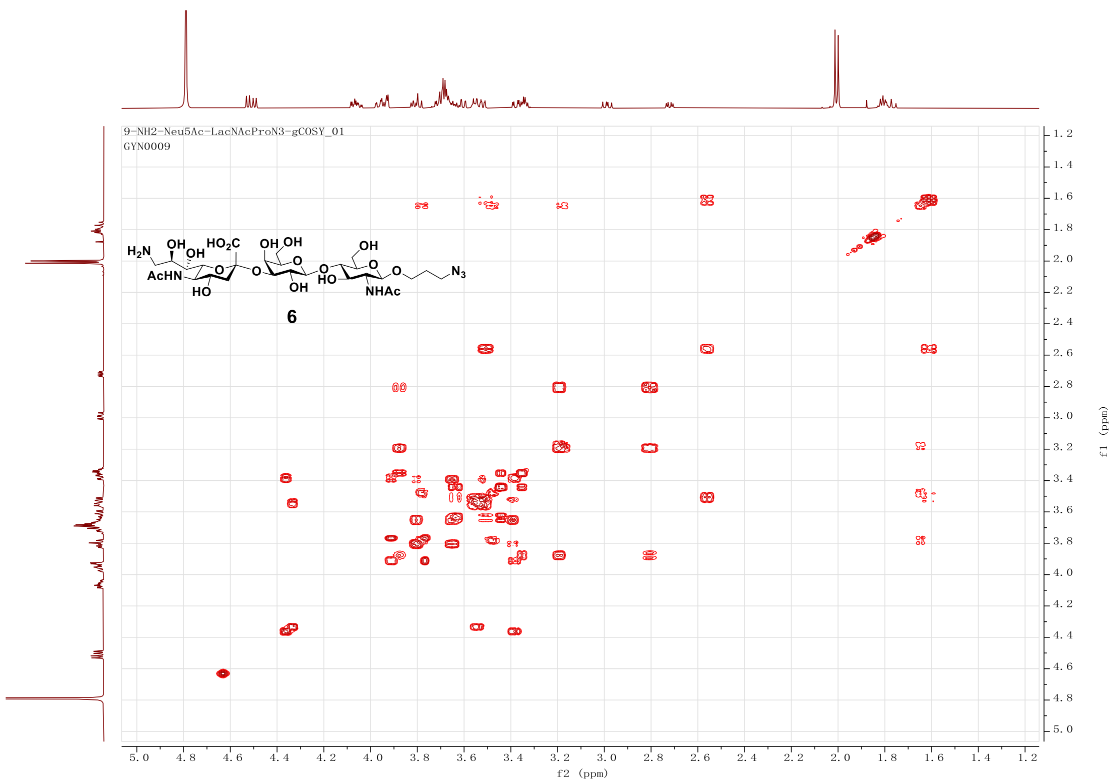
9-NH2-Neu5Ac-LacNAcProN3-gCOSY_01
GYN0009
H2N
OH
OH
HO2C
OH OH
OH
AcHN
HO
O
OH
HO
NHAc
N3
6
f2 (ppm)
f1 (ppm)

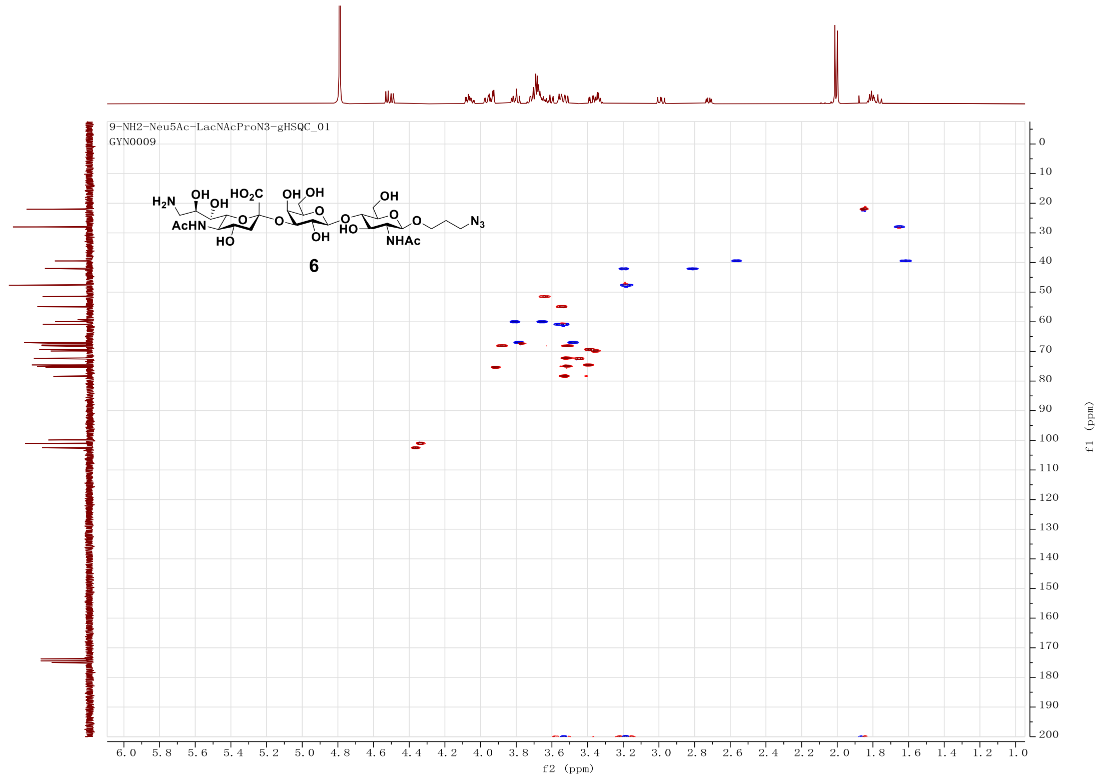
9-NH2-Neu5Ac-LacNAcProN3-gHSQC_01
GYN0009
H2N
OH
OH
HO2C
OH OH
OH
AcHN
HO
O
OH
HO
NHAc
N3
6
f2 (ppm)
f1 (ppm)

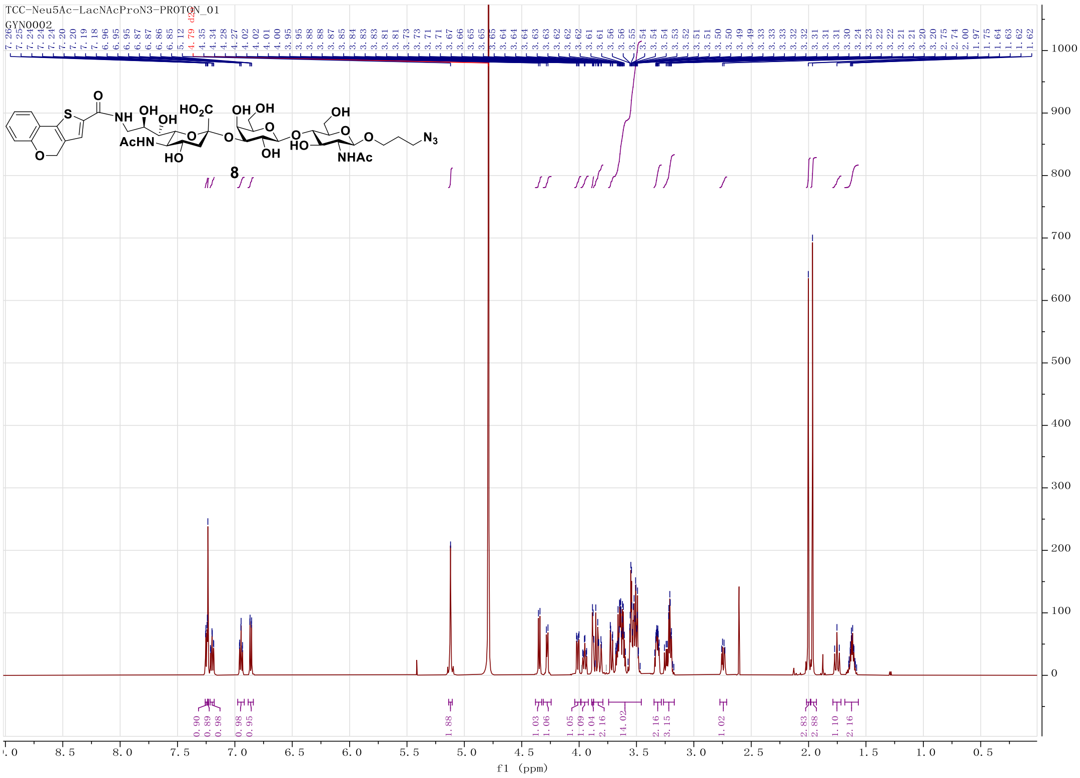
TCC-Neu5Ac-LacNAcProN3-PROTON_01
GYN0002
8
f1 (ppm)

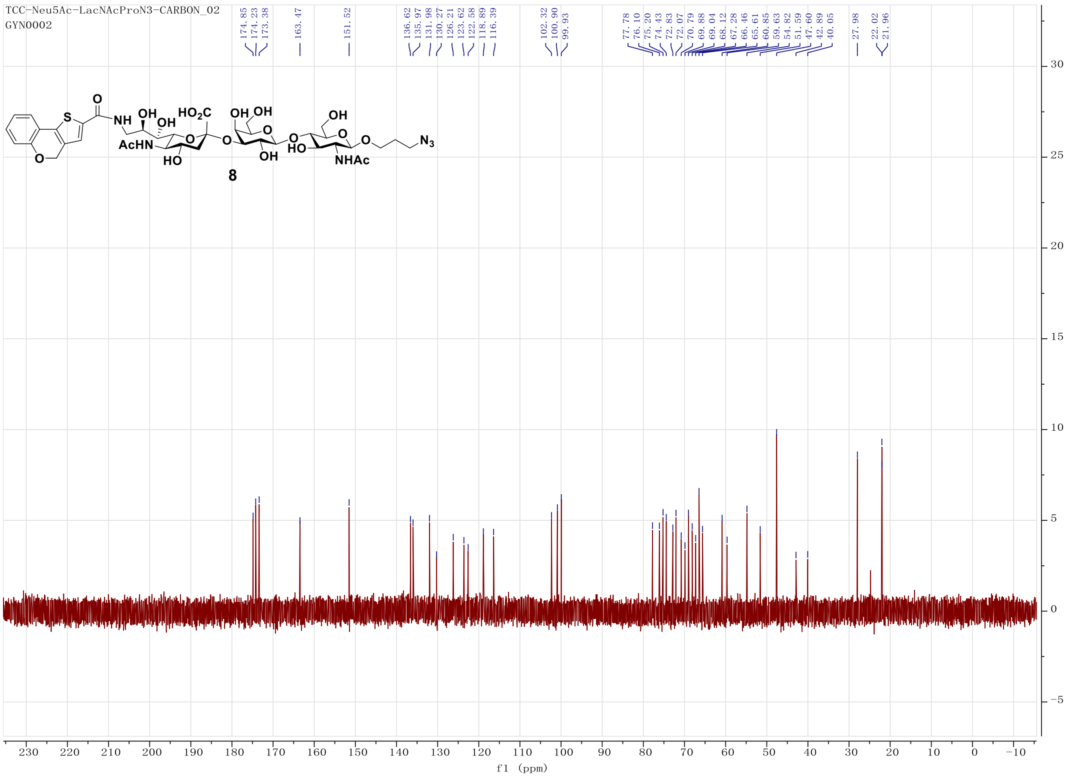
TCC-Neu5Ac-LacNAcProN3-CARBON_02
GYN0002
8
f1 (ppm)

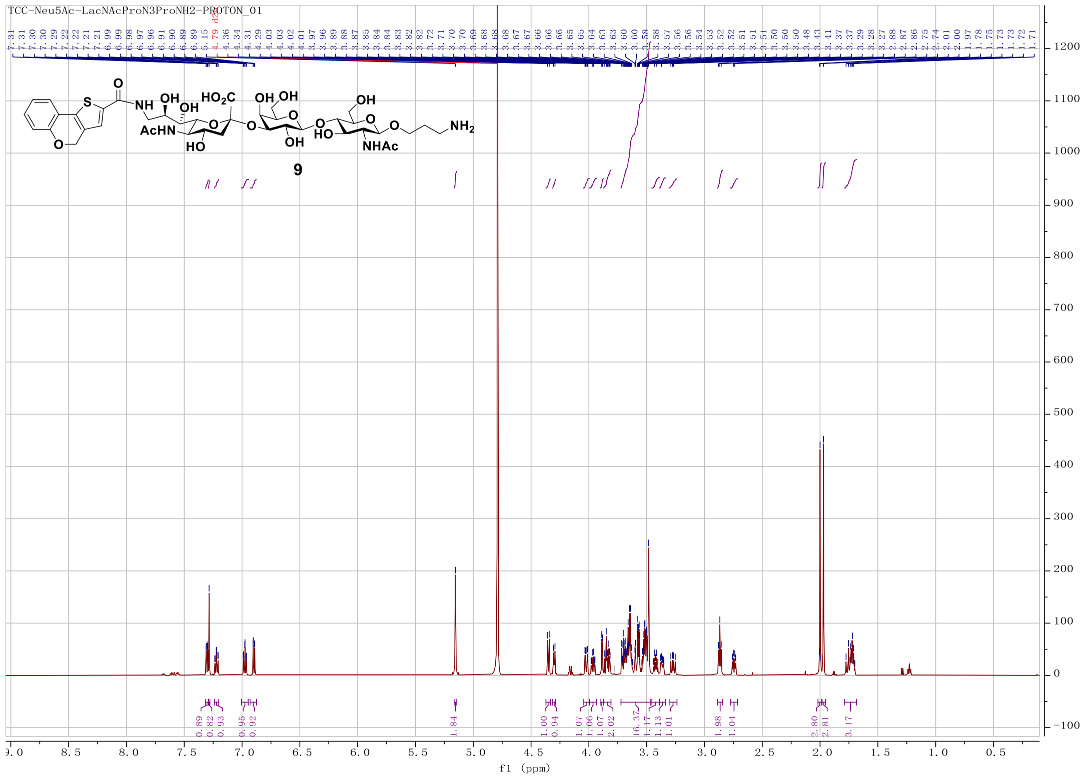

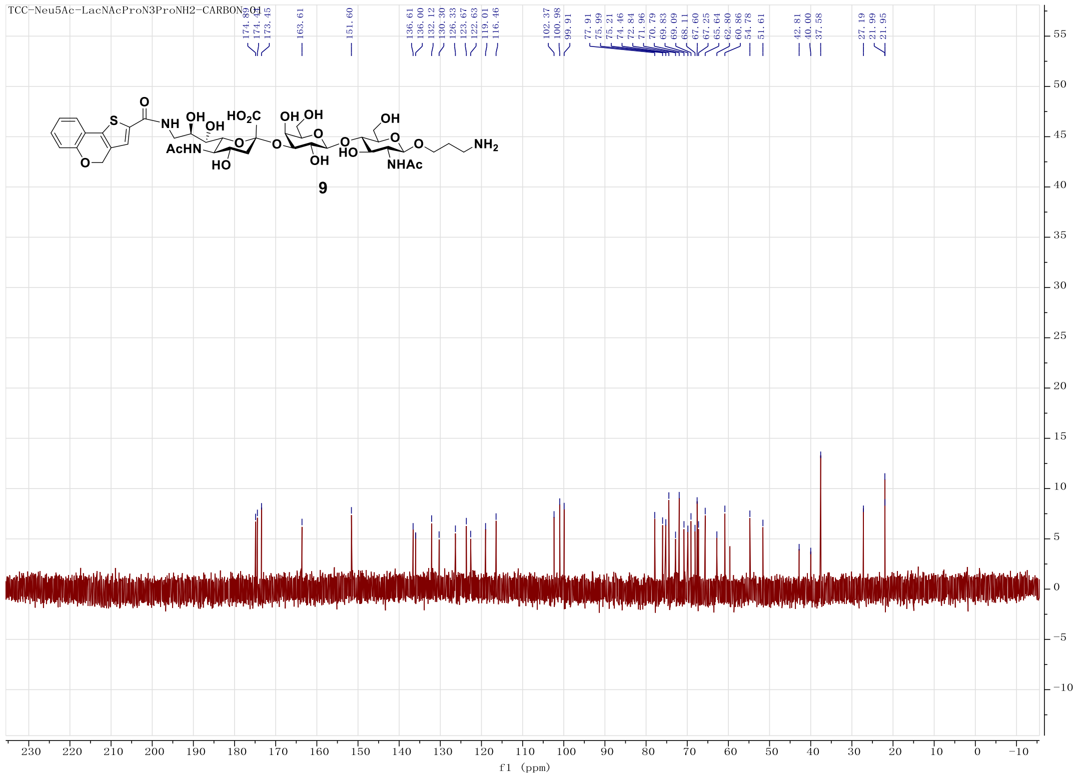

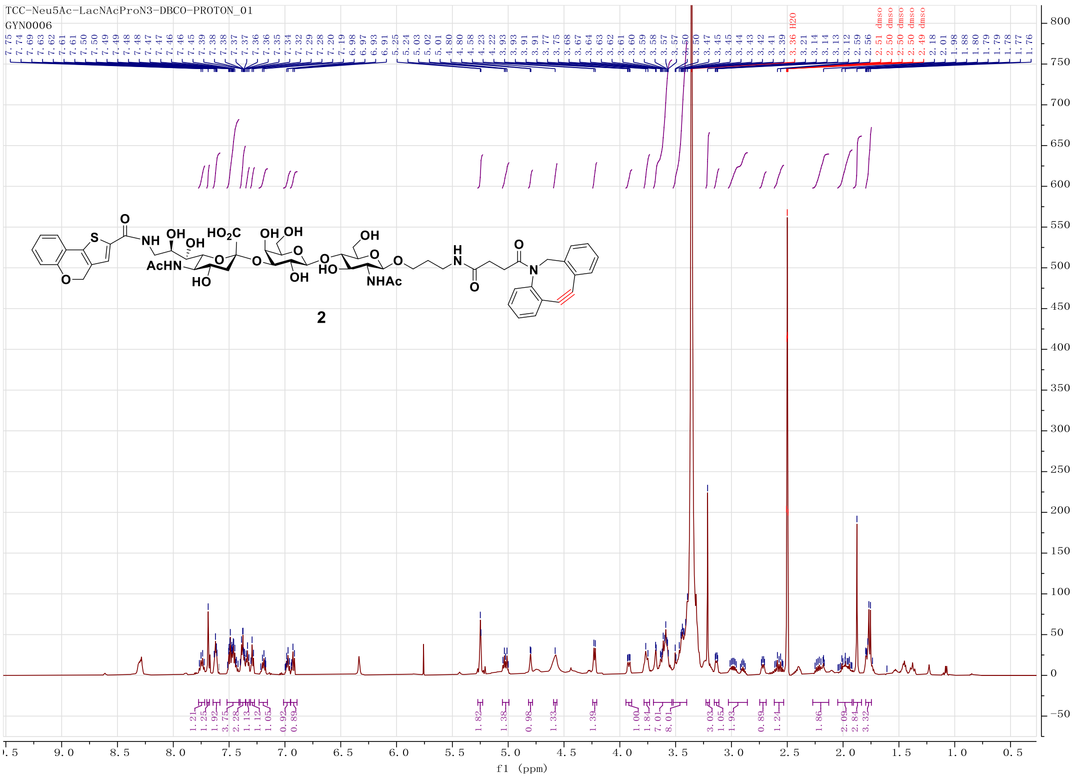
TCC-Neu5Ac-LacNAcProN3-DBCO-PROTON_01
GYN0006
2
f1 (ppm)

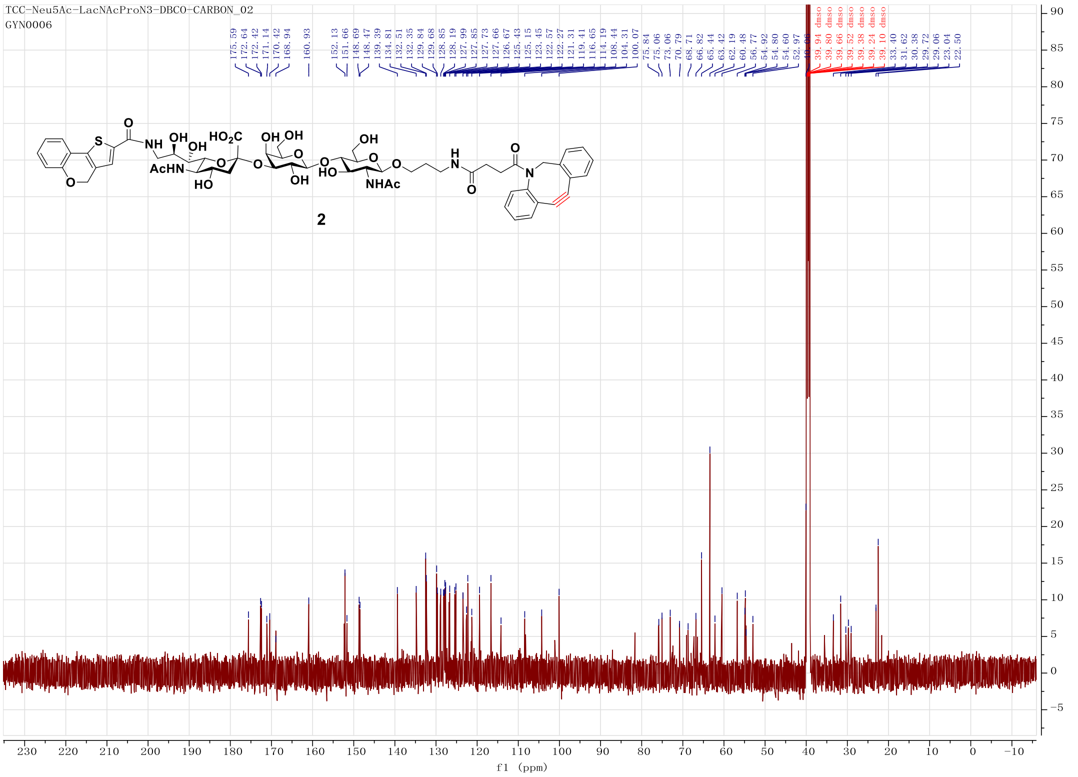
TCC-Neu5Ac-LacNAcProN3-DBCO-CARBON_02
GYN0006
2
f1 (ppm)

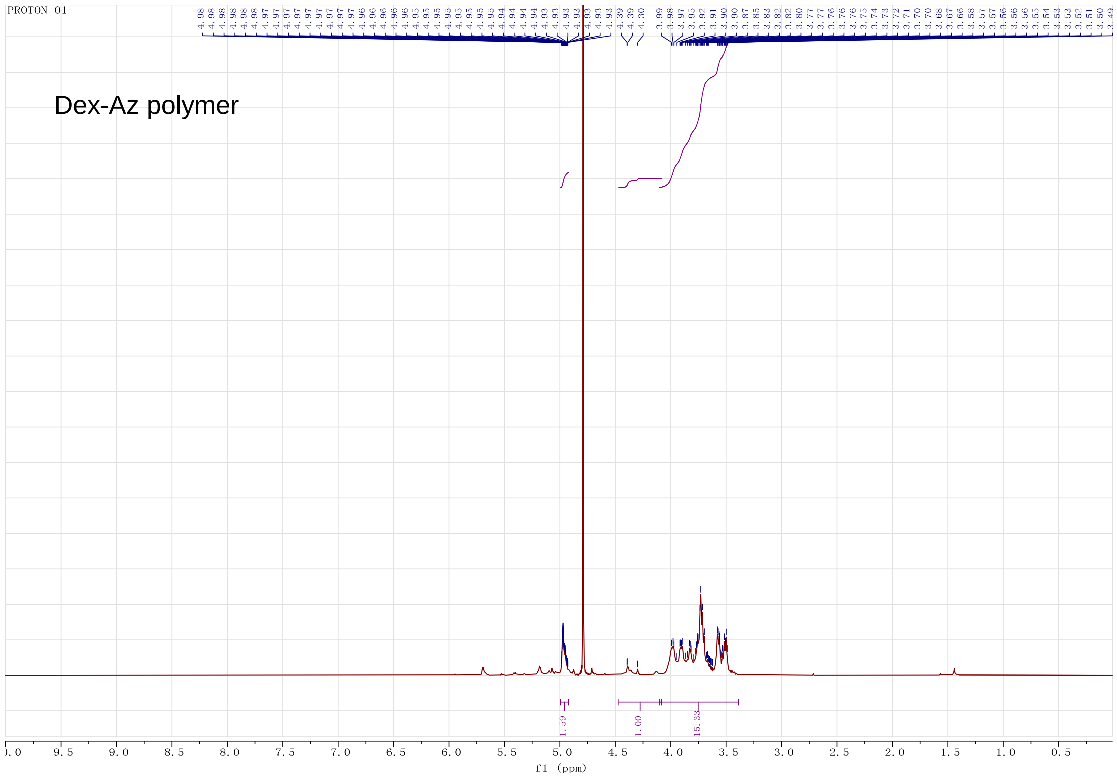
PROTON_01
Dex-Az polymer
f1 (ppm)

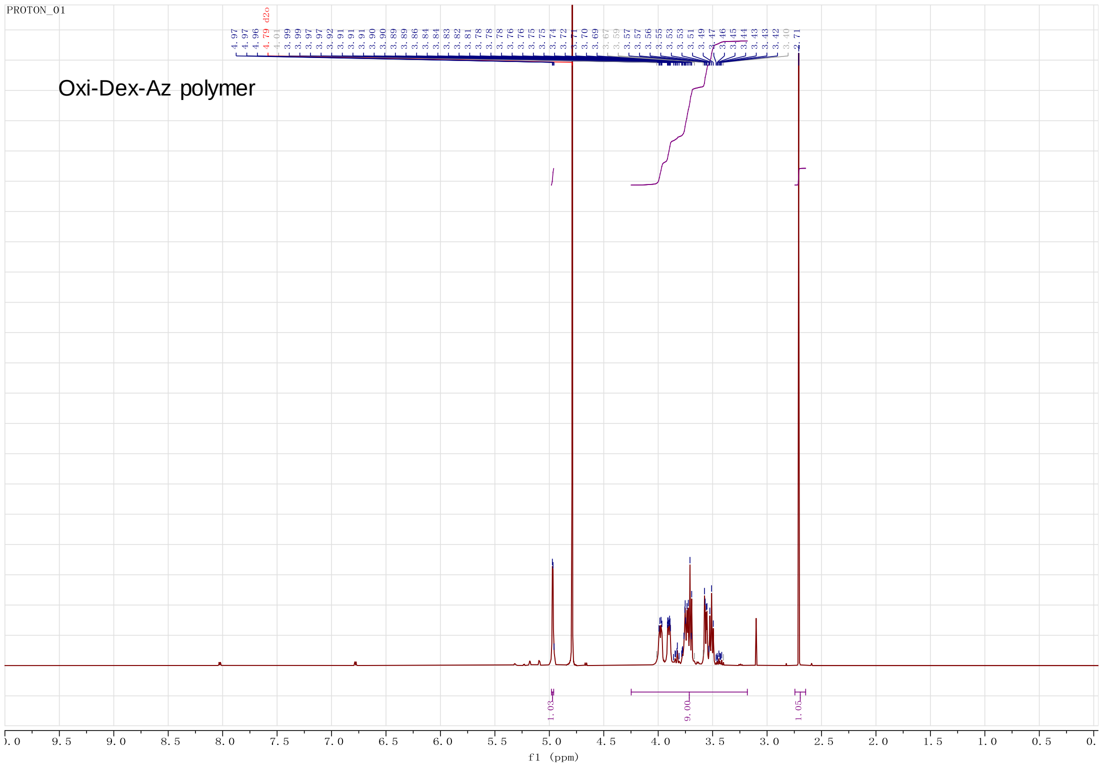
PROTON_01
Oxi-Dex-Az polymer
f1 (ppm)

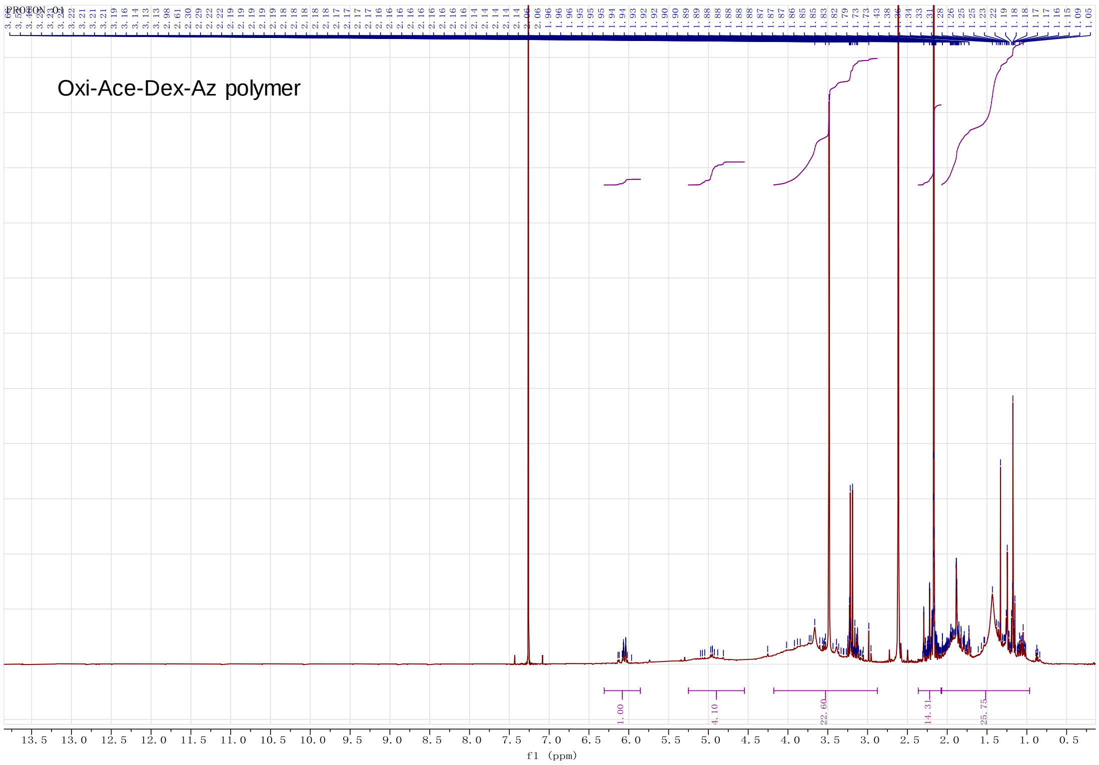

References

[1] a) H. Ma, W. Zeng, X. Meng, X. Huang, Y. Yang, D. Zhao, P. Zhou, X. Wang, C. Zhao, Y. Sun, P. Wang, H. Ou, X. Hu, Y. Xiang, T. Jin, *J. Virol.* **2021**, *95*; b) W. Zeng, G. Liu, H. Ma, D. Zhao, Y. Yang, M. Liu, A. Mohammed, C. Zhao, Y. Yang, J. Xie, C. Ding, X. Ma, J. Weng, Y. Gao, H. He, T. Jin, *Biochem. Biophys. Res. Commun.* **2020**, *527*, 618.

[2] P. Zhou, X. L. Yang, X. G. Wang, B. Hu, L. Zhang, W. Zhang, H. R. Si, Y. Zhu, B. Li, C. L. Huang, H. D. Chen, J. Chen, Y. Luo, H. Guo, R. D. Jiang, M. Q. Liu, Y. Chen, X. R. Shen, X. Wang, X. S. Zheng, K. Zhao, Q. J. Chen, F. Deng, L. L. Liu, B. Yan, F. X. Zhan, Y. Y. Wang, G. F. Xiao, Z. L. Shi, *Nature* **2020**, *579*, 270.

[3] J. C. Paulson, S. Han, B. E. Collins, P. Bengtson, *Nat. Chem. Biol* **2005**, *1*, 93.

[4] Y. Gao, Q. Zhao, M. Xiao, X. Huang, X. Wu, *Biomaterials* **2021**, *273*, 120792.

[5] a) N. van Doremalen, T. Lambe, A. Spencer, S. Belij-Rammerstorfer, J. N. Purushotham, J. R. Port, V. A. Avanzato, T. Bushmaker, A. Flaxman, M. Ulaszewska, F. Feldmann, E. R. Allen, H. Sharpe, J. Schulz, M. Holbrook, A. Okumura, K. Meade-White, L. Pérez-Pérez, N. J. Edwards, D. Wright, C. Bissett, C. Gilbride, B. N. Williamson, R. Rosenke, D. Long, A. Ishwarbhai, R. Kailath, L. Rose, S. Morris, C. Powers, J. Lovaglio, P. W. Hanley, D. Scott, G.

Saturday, E. de Wit, S. C. Gilbert, V. J. Munster, *Nature* **2020**, *586*, 578; b) Z. Wu, Y. Hu, M. Xu, Z. Chen, W. Yang, Z. Jiang, M. Li, H. Jin, G. Cui, P. Chen, L. Wang, G. Zhao, Y. Ding, Y. Zhao, W. Yin, *Lancet Infect. Dis.* **2021**, *21*, 803.

[6] M. M. Tomayko, C. P. Reynolds, *Cancer Chemother. Pharmacol.* **1989**, *24*, 148.

[7] a) H. Schmidt, *Front. Immunol.* **2021**, *12*, 679841; b) A. Sokal, P. Chappert, G. Barba-Spaeth, A. Roeser, S. Fourati, I. Azzaoui, A. Vandenberghe, I. Fernandez, A. Meola, M. Bouvier-Alias, E. Crickx, A. Beldi-Ferchiou, S. Hue, L. Languille, M. Michel, S. Baloul, F. Noizat-Pirenne, M. Luka, J. Mégret, M. Ménager, J.-M. Pawlotsky, S. Fillatreau, F. A. Rey, J.-C. Weill, C.-A. Reynaud, M. Mahévas, *Cell* **2021**, *184*, 1201.

[8] Q. Zhang, H. Zhang, J. Gao, K. Huang, Y. Yang, X. Hui, X. He, C. Li, W. Gong, Y. Zhang, Y. Zhao, C. Peng, X. Gao, H. Chen, Z. Zou, Z.-L. Shi, M. Jin, *Emerg. Microbes Infect.* **2020**, *9*, 2013.
